# The statistics of how natural images drive the responses of neurons

**DOI:** 10.1167/19.13.4

**Published:** 2019-11-05

**Authors:** Arvind Iyer, Johannes Burge

**Affiliations:** arvindiy@sas.upenn.edu; jburge@sas.upenn.edu; Department of Psychology, University of Pennsylvania, Philadelphia, PA, USA; Department of Psychology, University of Pennsylvania, Philadelphia, PA, USA; Neuroscience Graduate Group, University of Pennsylvania, Philadelphia, PA, USA; Bioengineering Graduate Group, University of Pennsylvania, Philadelphia, PA, USA

**Keywords:** *natural-scene statistics*, *natural images*, *simple cell*, *early visual cortex*, *receptive field*, *broadband normalization*, *narrowband normalization*, *encoding noise*, *contrast*, *similarity*, *sensitivity*

## Abstract

To model the responses of neurons in the early visual system, at least three basic components are required: a receptive field, a normalization term, and a specification of encoding noise. Here, we examine how the receptive field, the normalization factor, and the encoding noise affect the drive to model-neuron responses when stimulated with natural images. We show that when these components are modeled appropriately, the response drives elicited by natural stimuli are Gaussian-distributed and scale invariant, and very nearly maximize the sensitivity (*d*′) for natural-image discrimination. We discuss the statistical models of natural stimuli that can account for these response statistics, and we show how some commonly used modeling practices may distort these results. Finally, we show that normalization can equalize important properties of neural response across different stimulus types. Specifically, narrowband (stimulus- and feature-specific) normalization causes model neurons to yield Gaussian response-drive statistics when stimulated with natural stimuli, 1/*f* noise stimuli, and white-noise stimuli. The current work makes recommendations for best practices and lays a foundation, grounded in the response statistics to natural stimuli, upon which to build principled models of more complex visual tasks.

## Introduction

As interest intensifies in understanding natural signals in vision and neuroscience, it becomes increasingly important to develop a clear picture of how neural systems and their constituent components respond to real-world (photographic) images. Characterizing the statistical properties of these responses is vital for building principled models of visual processing, especially given the increasing reliance of vision and visual neuroscience on probability theory (Knill & Richards, 1996). Over the past two decades, there have been many neurophysiological (Baddeley et al., 1997; Baudot et al., 2013; Burkhardt, Fahey, & Sikora, 2006; Butts et al., 2010; Felsen, Touryan, Han, & Dan, 2005; Goris, Simoncelli, & Movshon, 2015; Lesica et al., 2007; Talebi & Baker, 2012; Vinje & Gallant, 2000; Weliky, Fiser, Hunt, & Wagner, 2003) and computational (Brady & Field, 2000; Burge & Geisler, 2014, 2015; Clatworthy, Chirimuuta, Lauritzen, & Tolhurst, 2003; Lyu & Simoncelli, 2008, 2009b; Sebastian, Abrams, & Geisler, 2017; Tadmor & Tolhurst, 2000; Wainwright & Simoncelli, 2000) attempts to address this issue.

We report a large-scale analysis of how natural images drive the responses of neurons. To model neural responses, at least three basic components are required: a receptive field, a normalization factor, and a specification of encoding noise. Each component of this image-encoding model plays a role in shaping the response-drive statistics that natural images elicit. The receptive field specifies the neuron's preferred stimulus feature and indicates how inputs are linearly weighted and summed (i.e., pooled) across space (Hubel & Wiesel, 1962, 1968). The normalization factor determines the impact of gain control (Albrecht & Geisler, 1991; Heeger, 1992). The encoding noise specifies variability given repeated presentations of the same stimulus (Tolhurst, Movshon, & Dean, 1983). The input stimulus, receptive field, normalization factor, and encoding noise together determine the noisy response drive. Response drive then goes through an output nonlinearity to obtain membrane potential or spiking response.

This article focuses on how the components of a standard image-encoding model shape responses to natural stimuli. We examine how methods for modeling the receptive field, the normalization factor, and the encoding noise affect response-drive statistics and the sensitivity for natural-stimulus discrimination. We focus our analysis on model neurons with oriented receptive fields like those in early visual cortex. We show that feature-specific normalization of each stimulus, with an easy-to-compute normalization factor, yields model-neuron response drives that are approximately Gaussian distributed across natural images and with standard deviations that are invariant to the scale of the preferred feature. Response drives with these statistics nearly maximize the sensitivity for stimulus discrimination for multiple common models of encoding noise.

Analysis of response-drive statistics can help enrich our understanding of how and why spiking responses in cortex take the form that they do. Real neurons produce highly non-Gaussian (i.e., heavy-tailed or sparse) spiking responses when stimulated with natural images (Baddeley et al., 1997; Vinje & Gallant, 2000). In the Discussion, we show that when the Gaussian-distributed response drives predicted by our analyses are pushed through nonlinearities like those performed by simple and complex cells in cortex, the results are consistent with, and may help explain, observed distributions of spiking response. Also in the Discussion, we consider the impact of cross-orientation and surround suppression.

This article provides a number of recommendations for how to increase the realism while maintaining the tractability of nonlinear image-encoding models inspired by the early visual system. The computational-vision and systems-neuroscience communities should benefit from these results. Subtle variants of the standard response model—which are used across the vision, image-processing, and computational-neuroscience communities—have notable impacts on the results described. To achieve scientific consensus on basic facts about natural-stimulus processing, we must understand how different image-encoding models affect response statistics.

## Results

How do common receptive-field modeling choices affect response-drive statistics and natural-image discrimination? First, we review the relation between response variability and stimulus discriminability. Second, we describe a common model of neural response in early visual cortex and show how two different forms of normalization impact the response statistics and natural-stimulus discriminability. Third, we discuss the statistical models of natural stimuli that can account for these results.

### Stimulus discriminability from neural response

Consider a model neuron that produces a particular response-drive distribution across tens of thousands of natural stimuli. Any early visual representation must be capable of distinguishing two arbitrary stimuli from one another. How well can a neuron distinguish two arbitrary stimuli from the ensemble of natural stimuli? To assess sensitivity (*d*′) for discrimination given the response drive of a particular neuron, we compute the discriminability of two arbitrary stimuli, randomly sampled from the natural-stimulus ensemble.

The discriminability (i.e., *d*′) of any two stimuli based this neuron's response is given by

\newcommand{\bialpha}{\boldsymbol{\alpha}}\newcommand{\bibeta}{\boldsymbol{\beta}}\newcommand{\bigamma}{\boldsymbol{\gamma}}\newcommand{\bidelta}{\boldsymbol{\delta}}\newcommand{\bivarepsilon}{\boldsymbol{\varepsilon}}\newcommand{\bizeta}{\boldsymbol{\zeta}}\newcommand{\bieta}{\boldsymbol{\eta}}\newcommand{\bitheta}{\boldsymbol{\theta}}\newcommand{\biiota}{\boldsymbol{\iota}}\newcommand{\bikappa}{\boldsymbol{\kappa}}\newcommand{\bilambda}{\boldsymbol{\lambda}}\newcommand{\bimu}{\boldsymbol{\mu}}\newcommand{\binu}{\boldsymbol{\nu}}\newcommand{\bixi}{\boldsymbol{\xi}}\newcommand{\biomicron}{\boldsymbol{\micron}}\newcommand{\bipi}{\boldsymbol{\pi}}\newcommand{\birho}{\boldsymbol{\rho}}\newcommand{\bisigma}{\boldsymbol{\sigma}}\newcommand{\bitau}{\boldsymbol{\tau}}\newcommand{\biupsilon}{\boldsymbol{\upsilon}}\newcommand{\biphi}{\boldsymbol{\phi}}\newcommand{\bichi}{\boldsymbol{\chi}}\newcommand{\bipsi}{\boldsymbol{\psi}}\newcommand{\biomega}{\boldsymbol{\omega}}\begin{equation}\tag{1}{d^{\prime} _{ij}} = {{\left| {{r_{i}} - {r_{j}}} \right|} \over {{\sigma _I}}}\end{equation}

where $\newcommand{\bialpha}{\boldsymbol{\alpha}}\newcommand{\bibeta}{\boldsymbol{\beta}}\newcommand{\bigamma}{\boldsymbol{\gamma}}\newcommand{\bidelta}{\boldsymbol{\delta}}\newcommand{\bivarepsilon}{\boldsymbol{\varepsilon}}\newcommand{\bizeta}{\boldsymbol{\zeta}}\newcommand{\bieta}{\boldsymbol{\eta}}\newcommand{\bitheta}{\boldsymbol{\theta}}\newcommand{\biiota}{\boldsymbol{\iota}}\newcommand{\bikappa}{\boldsymbol{\kappa}}\newcommand{\bilambda}{\boldsymbol{\lambda}}\newcommand{\bimu}{\boldsymbol{\mu}}\newcommand{\binu}{\boldsymbol{\nu}}\newcommand{\bixi}{\boldsymbol{\xi}}\newcommand{\biomicron}{\boldsymbol{\micron}}\newcommand{\bipi}{\boldsymbol{\pi}}\newcommand{\birho}{\boldsymbol{\rho}}\newcommand{\bisigma}{\boldsymbol{\sigma}}\newcommand{\bitau}{\boldsymbol{\tau}}\newcommand{\biupsilon}{\boldsymbol{\upsilon}}\newcommand{\biphi}{\boldsymbol{\phi}}\newcommand{\bichi}{\boldsymbol{\chi}}\newcommand{\bipsi}{\boldsymbol{\psi}}\newcommand{\biomega}{\boldsymbol{\omega}}{r_{i}} = E\left[ {R|{s_i}} \right]$ and $\newcommand{\bialpha}{\boldsymbol{\alpha}}\newcommand{\bibeta}{\boldsymbol{\beta}}\newcommand{\bigamma}{\boldsymbol{\gamma}}\newcommand{\bidelta}{\boldsymbol{\delta}}\newcommand{\bivarepsilon}{\boldsymbol{\varepsilon}}\newcommand{\bizeta}{\boldsymbol{\zeta}}\newcommand{\bieta}{\boldsymbol{\eta}}\newcommand{\bitheta}{\boldsymbol{\theta}}\newcommand{\biiota}{\boldsymbol{\iota}}\newcommand{\bikappa}{\boldsymbol{\kappa}}\newcommand{\bilambda}{\boldsymbol{\lambda}}\newcommand{\bimu}{\boldsymbol{\mu}}\newcommand{\binu}{\boldsymbol{\nu}}\newcommand{\bixi}{\boldsymbol{\xi}}\newcommand{\biomicron}{\boldsymbol{\micron}}\newcommand{\bipi}{\boldsymbol{\pi}}\newcommand{\birho}{\boldsymbol{\rho}}\newcommand{\bisigma}{\boldsymbol{\sigma}}\newcommand{\bitau}{\boldsymbol{\tau}}\newcommand{\biupsilon}{\boldsymbol{\upsilon}}\newcommand{\biphi}{\boldsymbol{\phi}}\newcommand{\bichi}{\boldsymbol{\chi}}\newcommand{\bipsi}{\boldsymbol{\psi}}\newcommand{\biomega}{\boldsymbol{\omega}}{r_{j}} = E\left[ {R|{s_j}} \right]$ represent the expected model-neuron response drives to two randomly sampled stimuli $\newcommand{\bialpha}{\boldsymbol{\alpha}}\newcommand{\bibeta}{\boldsymbol{\beta}}\newcommand{\bigamma}{\boldsymbol{\gamma}}\newcommand{\bidelta}{\boldsymbol{\delta}}\newcommand{\bivarepsilon}{\boldsymbol{\varepsilon}}\newcommand{\bizeta}{\boldsymbol{\zeta}}\newcommand{\bieta}{\boldsymbol{\eta}}\newcommand{\bitheta}{\boldsymbol{\theta}}\newcommand{\biiota}{\boldsymbol{\iota}}\newcommand{\bikappa}{\boldsymbol{\kappa}}\newcommand{\bilambda}{\boldsymbol{\lambda}}\newcommand{\bimu}{\boldsymbol{\mu}}\newcommand{\binu}{\boldsymbol{\nu}}\newcommand{\bixi}{\boldsymbol{\xi}}\newcommand{\biomicron}{\boldsymbol{\micron}}\newcommand{\bipi}{\boldsymbol{\pi}}\newcommand{\birho}{\boldsymbol{\rho}}\newcommand{\bisigma}{\boldsymbol{\sigma}}\newcommand{\bitau}{\boldsymbol{\tau}}\newcommand{\biupsilon}{\boldsymbol{\upsilon}}\newcommand{\biphi}{\boldsymbol{\phi}}\newcommand{\bichi}{\boldsymbol{\chi}}\newcommand{\bipsi}{\boldsymbol{\psi}}\newcommand{\biomega}{\boldsymbol{\omega}}{s_i}$ and $\newcommand{\bialpha}{\boldsymbol{\alpha}}\newcommand{\bibeta}{\boldsymbol{\beta}}\newcommand{\bigamma}{\boldsymbol{\gamma}}\newcommand{\bidelta}{\boldsymbol{\delta}}\newcommand{\bivarepsilon}{\boldsymbol{\varepsilon}}\newcommand{\bizeta}{\boldsymbol{\zeta}}\newcommand{\bieta}{\boldsymbol{\eta}}\newcommand{\bitheta}{\boldsymbol{\theta}}\newcommand{\biiota}{\boldsymbol{\iota}}\newcommand{\bikappa}{\boldsymbol{\kappa}}\newcommand{\bilambda}{\boldsymbol{\lambda}}\newcommand{\bimu}{\boldsymbol{\mu}}\newcommand{\binu}{\boldsymbol{\nu}}\newcommand{\bixi}{\boldsymbol{\xi}}\newcommand{\biomicron}{\boldsymbol{\micron}}\newcommand{\bipi}{\boldsymbol{\pi}}\newcommand{\birho}{\boldsymbol{\rho}}\newcommand{\bisigma}{\boldsymbol{\sigma}}\newcommand{\bitau}{\boldsymbol{\tau}}\newcommand{\biupsilon}{\boldsymbol{\upsilon}}\newcommand{\biphi}{\boldsymbol{\phi}}\newcommand{\bichi}{\boldsymbol{\chi}}\newcommand{\bipsi}{\boldsymbol{\psi}}\newcommand{\biomega}{\boldsymbol{\omega}}{s_j}$, and $\newcommand{\bialpha}{\boldsymbol{\alpha}}\newcommand{\bibeta}{\boldsymbol{\beta}}\newcommand{\bigamma}{\boldsymbol{\gamma}}\newcommand{\bidelta}{\boldsymbol{\delta}}\newcommand{\bivarepsilon}{\boldsymbol{\varepsilon}}\newcommand{\bizeta}{\boldsymbol{\zeta}}\newcommand{\bieta}{\boldsymbol{\eta}}\newcommand{\bitheta}{\boldsymbol{\theta}}\newcommand{\biiota}{\boldsymbol{\iota}}\newcommand{\bikappa}{\boldsymbol{\kappa}}\newcommand{\bilambda}{\boldsymbol{\lambda}}\newcommand{\bimu}{\boldsymbol{\mu}}\newcommand{\binu}{\boldsymbol{\nu}}\newcommand{\bixi}{\boldsymbol{\xi}}\newcommand{\biomicron}{\boldsymbol{\micron}}\newcommand{\bipi}{\boldsymbol{\pi}}\newcommand{\birho}{\boldsymbol{\rho}}\newcommand{\bisigma}{\boldsymbol{\sigma}}\newcommand{\bitau}{\boldsymbol{\tau}}\newcommand{\biupsilon}{\boldsymbol{\upsilon}}\newcommand{\biphi}{\boldsymbol{\phi}}\newcommand{\bichi}{\boldsymbol{\chi}}\newcommand{\bipsi}{\boldsymbol{\psi}}\newcommand{\biomega}{\boldsymbol{\omega}}{\sigma _I}$ represents internal encoding noise (Green & Swets, 1966). (The expression specifying how noisy response drive $\newcommand{\bialpha}{\boldsymbol{\alpha}}\newcommand{\bibeta}{\boldsymbol{\beta}}\newcommand{\bigamma}{\boldsymbol{\gamma}}\newcommand{\bidelta}{\boldsymbol{\delta}}\newcommand{\bivarepsilon}{\boldsymbol{\varepsilon}}\newcommand{\bizeta}{\boldsymbol{\zeta}}\newcommand{\bieta}{\boldsymbol{\eta}}\newcommand{\bitheta}{\boldsymbol{\theta}}\newcommand{\biiota}{\boldsymbol{\iota}}\newcommand{\bikappa}{\boldsymbol{\kappa}}\newcommand{\bilambda}{\boldsymbol{\lambda}}\newcommand{\bimu}{\boldsymbol{\mu}}\newcommand{\binu}{\boldsymbol{\nu}}\newcommand{\bixi}{\boldsymbol{\xi}}\newcommand{\biomicron}{\boldsymbol{\micron}}\newcommand{\bipi}{\boldsymbol{\pi}}\newcommand{\birho}{\boldsymbol{\rho}}\newcommand{\bisigma}{\boldsymbol{\sigma}}\newcommand{\bitau}{\boldsymbol{\tau}}\newcommand{\biupsilon}{\boldsymbol{\upsilon}}\newcommand{\biphi}{\boldsymbol{\phi}}\newcommand{\bichi}{\boldsymbol{\chi}}\newcommand{\bipsi}{\boldsymbol{\psi}}\newcommand{\biomega}{\boldsymbol{\omega}}R$ is computed for a given stimulus is provided in the next section.)

The distribution of expected response drives across natural stimuli $\newcommand{\bialpha}{\boldsymbol{\alpha}}\newcommand{\bibeta}{\boldsymbol{\beta}}\newcommand{\bigamma}{\boldsymbol{\gamma}}\newcommand{\bidelta}{\boldsymbol{\delta}}\newcommand{\bivarepsilon}{\boldsymbol{\varepsilon}}\newcommand{\bizeta}{\boldsymbol{\zeta}}\newcommand{\bieta}{\boldsymbol{\eta}}\newcommand{\bitheta}{\boldsymbol{\theta}}\newcommand{\biiota}{\boldsymbol{\iota}}\newcommand{\bikappa}{\boldsymbol{\kappa}}\newcommand{\bilambda}{\boldsymbol{\lambda}}\newcommand{\bimu}{\boldsymbol{\mu}}\newcommand{\binu}{\boldsymbol{\nu}}\newcommand{\bixi}{\boldsymbol{\xi}}\newcommand{\biomicron}{\boldsymbol{\micron}}\newcommand{\bipi}{\boldsymbol{\pi}}\newcommand{\birho}{\boldsymbol{\rho}}\newcommand{\bisigma}{\boldsymbol{\sigma}}\newcommand{\bitau}{\boldsymbol{\tau}}\newcommand{\biupsilon}{\boldsymbol{\upsilon}}\newcommand{\biphi}{\boldsymbol{\phi}}\newcommand{\bichi}{\boldsymbol{\chi}}\newcommand{\bipsi}{\boldsymbol{\psi}}\newcommand{\biomega}{\boldsymbol{\omega}}p\left( r \right) = \sum\nolimits_u {p\left( {r|{s_u}} \right)p\left( {{s_u}} \right)}$ has a critical impact on discriminability. Assuming that expected response drives to natural stimuli are Gaussian distributed $\newcommand{\bialpha}{\boldsymbol{\alpha}}\newcommand{\bibeta}{\boldsymbol{\beta}}\newcommand{\bigamma}{\boldsymbol{\gamma}}\newcommand{\bidelta}{\boldsymbol{\delta}}\newcommand{\bivarepsilon}{\boldsymbol{\varepsilon}}\newcommand{\bizeta}{\boldsymbol{\zeta}}\newcommand{\bieta}{\boldsymbol{\eta}}\newcommand{\bitheta}{\boldsymbol{\theta}}\newcommand{\biiota}{\boldsymbol{\iota}}\newcommand{\bikappa}{\boldsymbol{\kappa}}\newcommand{\bilambda}{\boldsymbol{\lambda}}\newcommand{\bimu}{\boldsymbol{\mu}}\newcommand{\binu}{\boldsymbol{\nu}}\newcommand{\bixi}{\boldsymbol{\xi}}\newcommand{\biomicron}{\boldsymbol{\micron}}\newcommand{\bipi}{\boldsymbol{\pi}}\newcommand{\birho}{\boldsymbol{\rho}}\newcommand{\bisigma}{\boldsymbol{\sigma}}\newcommand{\bitau}{\boldsymbol{\tau}}\newcommand{\biupsilon}{\boldsymbol{\upsilon}}\newcommand{\biphi}{\boldsymbol{\phi}}\newcommand{\bichi}{\boldsymbol{\chi}}\newcommand{\bipsi}{\boldsymbol{\psi}}\newcommand{\biomega}{\boldsymbol{\omega}}p\left( r \right) = N\left( {0,\sigma _E^2} \right)$, the expected discriminability across all stimuli is given by

**Figure 1:**
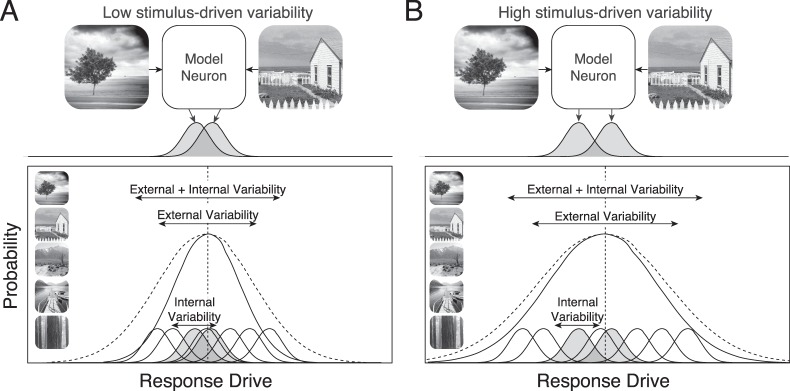
Stimulus-driven variability and the sensitivity for stimulus discrimination. (A) Model neuron yielding low stimulus-driven variance. Two natural images elicit response drives (shaded bell curves) that are hard to discriminate because of encoding noise (top). Low stimulus-driven response variability to the natural-image ensemble is associated with poor sensitivity for stimulus discrimination (bottom). Two random stimuli will tend to be hard to discriminate. (B) Model neuron with high stimulus-driven variance. The same two random stimuli are now easier to discriminate. Two randomly sampled stimuli from the natural-image ensemble will also tend to be easier to discriminate.

\newcommand{\bialpha}{\boldsymbol{\alpha}}\newcommand{\bibeta}{\boldsymbol{\beta}}\newcommand{\bigamma}{\boldsymbol{\gamma}}\newcommand{\bidelta}{\boldsymbol{\delta}}\newcommand{\bivarepsilon}{\boldsymbol{\varepsilon}}\newcommand{\bizeta}{\boldsymbol{\zeta}}\newcommand{\bieta}{\boldsymbol{\eta}}\newcommand{\bitheta}{\boldsymbol{\theta}}\newcommand{\biiota}{\boldsymbol{\iota}}\newcommand{\bikappa}{\boldsymbol{\kappa}}\newcommand{\bilambda}{\boldsymbol{\lambda}}\newcommand{\bimu}{\boldsymbol{\mu}}\newcommand{\binu}{\boldsymbol{\nu}}\newcommand{\bixi}{\boldsymbol{\xi}}\newcommand{\biomicron}{\boldsymbol{\micron}}\newcommand{\bipi}{\boldsymbol{\pi}}\newcommand{\birho}{\boldsymbol{\rho}}\newcommand{\bisigma}{\boldsymbol{\sigma}}\newcommand{\bitau}{\boldsymbol{\tau}}\newcommand{\biupsilon}{\boldsymbol{\upsilon}}\newcommand{\biphi}{\boldsymbol{\phi}}\newcommand{\bichi}{\boldsymbol{\chi}}\newcommand{\bipsi}{\boldsymbol{\psi}}\newcommand{\biomega}{\boldsymbol{\omega}}\begin{equation}\tag{2}E\left[ {d^{\prime} } \right] = {{{\sigma _E}} \over {{\sigma _I}}}{2 \over {\sqrt \pi }}{\rm ,}\end{equation}

where $\newcommand{\bialpha}{\boldsymbol{\alpha}}\newcommand{\bibeta}{\boldsymbol{\beta}}\newcommand{\bigamma}{\boldsymbol{\gamma}}\newcommand{\bidelta}{\boldsymbol{\delta}}\newcommand{\bivarepsilon}{\boldsymbol{\varepsilon}}\newcommand{\bizeta}{\boldsymbol{\zeta}}\newcommand{\bieta}{\boldsymbol{\eta}}\newcommand{\bitheta}{\boldsymbol{\theta}}\newcommand{\biiota}{\boldsymbol{\iota}}\newcommand{\bikappa}{\boldsymbol{\kappa}}\newcommand{\bilambda}{\boldsymbol{\lambda}}\newcommand{\bimu}{\boldsymbol{\mu}}\newcommand{\binu}{\boldsymbol{\nu}}\newcommand{\bixi}{\boldsymbol{\xi}}\newcommand{\biomicron}{\boldsymbol{\micron}}\newcommand{\bipi}{\boldsymbol{\pi}}\newcommand{\birho}{\boldsymbol{\rho}}\newcommand{\bisigma}{\boldsymbol{\sigma}}\newcommand{\bitau}{\boldsymbol{\tau}}\newcommand{\biupsilon}{\boldsymbol{\upsilon}}\newcommand{\biphi}{\boldsymbol{\phi}}\newcommand{\bichi}{\boldsymbol{\chi}}\newcommand{\bipsi}{\boldsymbol{\psi}}\newcommand{\biomega}{\boldsymbol{\omega}}{\sigma _E}$ is the stimulus-driven variation in response drive (e.g., external noise; see Figure 1) and the expectation is taken across all stimulus pairs (see Supplementary File S1). (If responses are Laplace distributed, the expected discriminability is given by $\newcommand{\bialpha}{\boldsymbol{\alpha}}\newcommand{\bibeta}{\boldsymbol{\beta}}\newcommand{\bigamma}{\boldsymbol{\gamma}}\newcommand{\bidelta}{\boldsymbol{\delta}}\newcommand{\bivarepsilon}{\boldsymbol{\varepsilon}}\newcommand{\bizeta}{\boldsymbol{\zeta}}\newcommand{\bieta}{\boldsymbol{\eta}}\newcommand{\bitheta}{\boldsymbol{\theta}}\newcommand{\biiota}{\boldsymbol{\iota}}\newcommand{\bikappa}{\boldsymbol{\kappa}}\newcommand{\bilambda}{\boldsymbol{\lambda}}\newcommand{\bimu}{\boldsymbol{\mu}}\newcommand{\binu}{\boldsymbol{\nu}}\newcommand{\bixi}{\boldsymbol{\xi}}\newcommand{\biomicron}{\boldsymbol{\micron}}\newcommand{\bipi}{\boldsymbol{\pi}}\newcommand{\birho}{\boldsymbol{\rho}}\newcommand{\bisigma}{\boldsymbol{\sigma}}\newcommand{\bitau}{\boldsymbol{\tau}}\newcommand{\biupsilon}{\boldsymbol{\upsilon}}\newcommand{\biphi}{\boldsymbol{\phi}}\newcommand{\bichi}{\boldsymbol{\chi}}\newcommand{\bipsi}{\boldsymbol{\psi}}\newcommand{\biomega}{\boldsymbol{\omega}}E\left[ {d^{\prime} } \right] = {{{\sigma _E}} \over {{\sigma _I}}}{3 \over {2\sqrt 2 }}{\rm {;}}$ see Supplementary File S1). For an arbitrary response distribution, expected sensitivity can be computed using numerical methods. The fact that the stimulus-driven standard deviation is in the numerator of Equation 2 indicates that greater stimulus-driven response variation yields better stimulus discriminability.

Increased variance is usually associated with poorer stimulus discriminability (Ernst & Banks, 2002), so Equation 2 deserves further explanation. The source of the response variance is critical for determining whether it helps or hurts discrimination. Figure 1A shows a model neuron with low stimulus-driven variance. Figure 1B shows a model neuron with high stimulus-driven variance. The top row shows how encoding noise (shaded bell curves) limits discriminability for two natural stimuli. The bottom row shows the impact of stimulus-driven variability across the image ensemble. High stimulus-driven variability will tend to yield larger differences between the expected response drives to two random stimuli. Zero stimulus-driven variability, which might occur if the sensory afferents to a neuron were severed, would make stimulus discrimination impossible. Thus, discriminability improves when the source of the response variability is external and stimulus driven, and discriminability deteriorates when the source of the response variability is internal and due to noise. The design of the visual system is surely driven by tasks more sophisticated than stimulus discrimination, but it is a useful task around which to organize discussion.

### Model-neuron responses

**Figure 2:**
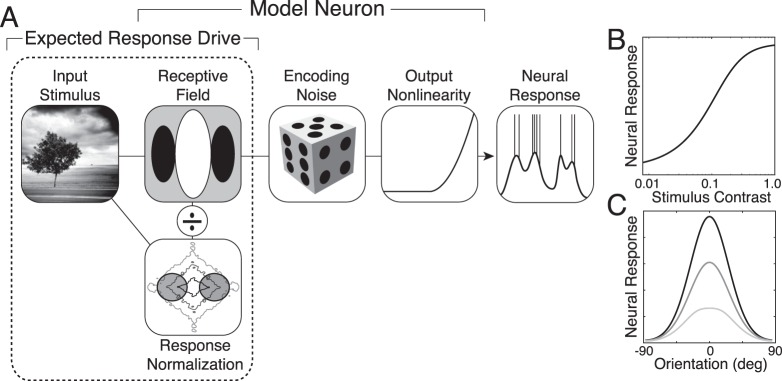
Model neuron. (A) Response model: linear filtering, response normalization, and encoding noise. The stimulus is encoded by a linear filter, normalized by a portion of the stimulus contrast, corrupted by encoding noise, and then pushed through an output nonlinearity. The resulting response provides a prediction of intracellular voltage or spike rate. This article focuses on response-drive statistics. (B) Contrast response function for the preferred stimulus: a vertical Gabor. (C) Orientation tuning function for three different spatial frequencies: Stimulus frequency equals the preferred spatial frequency (black), 2× the preferred spatial frequency (dark gray), and 3× the preferred spatial frequency (light gray).

Responses of neurons in early visual cortex are commonly modeled as arising from a series of processing stages (Figure 2A). First, a linear receptive field filters the stimulus to yield the linear response. Next, the linear response is normalized by a factor that is determined by local properties of the stimulus or by the responses of other neurons in a local pool (Albrecht & Geisler, 1991; Heeger, 1992). The normalized response is called the *response drive*. Then the response drive is corrupted by encoding noise. Many models of neural response also incorporate a static output nonlinearity. In the Discussion, we consider how output nonlinearities convert response drive into response rate, but our primary analysis is focused on the response-drive statistics.

More specifically, the noisy response drive $\newcommand{\bialpha}{\boldsymbol{\alpha}}\newcommand{\bibeta}{\boldsymbol{\beta}}\newcommand{\bigamma}{\boldsymbol{\gamma}}\newcommand{\bidelta}{\boldsymbol{\delta}}\newcommand{\bivarepsilon}{\boldsymbol{\varepsilon}}\newcommand{\bizeta}{\boldsymbol{\zeta}}\newcommand{\bieta}{\boldsymbol{\eta}}\newcommand{\bitheta}{\boldsymbol{\theta}}\newcommand{\biiota}{\boldsymbol{\iota}}\newcommand{\bikappa}{\boldsymbol{\kappa}}\newcommand{\bilambda}{\boldsymbol{\lambda}}\newcommand{\bimu}{\boldsymbol{\mu}}\newcommand{\binu}{\boldsymbol{\nu}}\newcommand{\bixi}{\boldsymbol{\xi}}\newcommand{\biomicron}{\boldsymbol{\micron}}\newcommand{\bipi}{\boldsymbol{\pi}}\newcommand{\birho}{\boldsymbol{\rho}}\newcommand{\bisigma}{\boldsymbol{\sigma}}\newcommand{\bitau}{\boldsymbol{\tau}}\newcommand{\biupsilon}{\boldsymbol{\upsilon}}\newcommand{\biphi}{\boldsymbol{\phi}}\newcommand{\bichi}{\boldsymbol{\chi}}\newcommand{\bipsi}{\boldsymbol{\psi}}\newcommand{\biomega}{\boldsymbol{\omega}}R$ to a particular stimulus is given by

\newcommand{\bialpha}{\boldsymbol{\alpha}}\newcommand{\bibeta}{\boldsymbol{\beta}}\newcommand{\bigamma}{\boldsymbol{\gamma}}\newcommand{\bidelta}{\boldsymbol{\delta}}\newcommand{\bivarepsilon}{\boldsymbol{\varepsilon}}\newcommand{\bizeta}{\boldsymbol{\zeta}}\newcommand{\bieta}{\boldsymbol{\eta}}\newcommand{\bitheta}{\boldsymbol{\theta}}\newcommand{\biiota}{\boldsymbol{\iota}}\newcommand{\bikappa}{\boldsymbol{\kappa}}\newcommand{\bilambda}{\boldsymbol{\lambda}}\newcommand{\bimu}{\boldsymbol{\mu}}\newcommand{\binu}{\boldsymbol{\nu}}\newcommand{\bixi}{\boldsymbol{\xi}}\newcommand{\biomicron}{\boldsymbol{\micron}}\newcommand{\bipi}{\boldsymbol{\pi}}\newcommand{\birho}{\boldsymbol{\rho}}\newcommand{\bisigma}{\boldsymbol{\sigma}}\newcommand{\bitau}{\boldsymbol{\tau}}\newcommand{\biupsilon}{\boldsymbol{\upsilon}}\newcommand{\biphi}{\boldsymbol{\phi}}\newcommand{\bichi}{\boldsymbol{\chi}}\newcommand{\bipsi}{\boldsymbol{\psi}}\newcommand{\biomega}{\boldsymbol{\omega}}\begin{equation}\tag{3}R = \overbrace {{r_{\max }}\left[ {{{{{\bf{f}}^{T}}{\bf{c}}} \mathord{\left/ {\vphantom {{{{\bf{f}}^{T}}{\bf{c}}} N}} \right. \kern-1.2pt} N}} \right]}^{\scriptstyle {\rm{expected}} \atop \scriptstyle {\rm{response\ drive}}} + {\rm{\ }}\varepsilon {\rm ,}\end{equation}

where $\newcommand{\bialpha}{\boldsymbol{\alpha}}\newcommand{\bibeta}{\boldsymbol{\beta}}\newcommand{\bigamma}{\boldsymbol{\gamma}}\newcommand{\bidelta}{\boldsymbol{\delta}}\newcommand{\bivarepsilon}{\boldsymbol{\varepsilon}}\newcommand{\bizeta}{\boldsymbol{\zeta}}\newcommand{\bieta}{\boldsymbol{\eta}}\newcommand{\bitheta}{\boldsymbol{\theta}}\newcommand{\biiota}{\boldsymbol{\iota}}\newcommand{\bikappa}{\boldsymbol{\kappa}}\newcommand{\bilambda}{\boldsymbol{\lambda}}\newcommand{\bimu}{\boldsymbol{\mu}}\newcommand{\binu}{\boldsymbol{\nu}}\newcommand{\bixi}{\boldsymbol{\xi}}\newcommand{\biomicron}{\boldsymbol{\micron}}\newcommand{\bipi}{\boldsymbol{\pi}}\newcommand{\birho}{\boldsymbol{\rho}}\newcommand{\bisigma}{\boldsymbol{\sigma}}\newcommand{\bitau}{\boldsymbol{\tau}}\newcommand{\biupsilon}{\boldsymbol{\upsilon}}\newcommand{\biphi}{\boldsymbol{\phi}}\newcommand{\bichi}{\boldsymbol{\chi}}\newcommand{\bipsi}{\boldsymbol{\psi}}\newcommand{\biomega}{\boldsymbol{\omega}}{r_{\max }}$ is the neuron's maximum response, $\newcommand{\bialpha}{\boldsymbol{\alpha}}\newcommand{\bibeta}{\boldsymbol{\beta}}\newcommand{\bigamma}{\boldsymbol{\gamma}}\newcommand{\bidelta}{\boldsymbol{\delta}}\newcommand{\bivarepsilon}{\boldsymbol{\varepsilon}}\newcommand{\bizeta}{\boldsymbol{\zeta}}\newcommand{\bieta}{\boldsymbol{\eta}}\newcommand{\bitheta}{\boldsymbol{\theta}}\newcommand{\biiota}{\boldsymbol{\iota}}\newcommand{\bikappa}{\boldsymbol{\kappa}}\newcommand{\bilambda}{\boldsymbol{\lambda}}\newcommand{\bimu}{\boldsymbol{\mu}}\newcommand{\binu}{\boldsymbol{\nu}}\newcommand{\bixi}{\boldsymbol{\xi}}\newcommand{\biomicron}{\boldsymbol{\micron}}\newcommand{\bipi}{\boldsymbol{\pi}}\newcommand{\birho}{\boldsymbol{\rho}}\newcommand{\bisigma}{\boldsymbol{\sigma}}\newcommand{\bitau}{\boldsymbol{\tau}}\newcommand{\biupsilon}{\boldsymbol{\upsilon}}\newcommand{\biphi}{\boldsymbol{\phi}}\newcommand{\bichi}{\boldsymbol{\chi}}\newcommand{\bipsi}{\boldsymbol{\psi}}\newcommand{\biomega}{\boldsymbol{\omega}}{\bf{f}}$ is the receptive field, $\newcommand{\bialpha}{\boldsymbol{\alpha}}\newcommand{\bibeta}{\boldsymbol{\beta}}\newcommand{\bigamma}{\boldsymbol{\gamma}}\newcommand{\bidelta}{\boldsymbol{\delta}}\newcommand{\bivarepsilon}{\boldsymbol{\varepsilon}}\newcommand{\bizeta}{\boldsymbol{\zeta}}\newcommand{\bieta}{\boldsymbol{\eta}}\newcommand{\bitheta}{\boldsymbol{\theta}}\newcommand{\biiota}{\boldsymbol{\iota}}\newcommand{\bikappa}{\boldsymbol{\kappa}}\newcommand{\bilambda}{\boldsymbol{\lambda}}\newcommand{\bimu}{\boldsymbol{\mu}}\newcommand{\binu}{\boldsymbol{\nu}}\newcommand{\bixi}{\boldsymbol{\xi}}\newcommand{\biomicron}{\boldsymbol{\micron}}\newcommand{\bipi}{\boldsymbol{\pi}}\newcommand{\birho}{\boldsymbol{\rho}}\newcommand{\bisigma}{\boldsymbol{\sigma}}\newcommand{\bitau}{\boldsymbol{\tau}}\newcommand{\biupsilon}{\boldsymbol{\upsilon}}\newcommand{\biphi}{\boldsymbol{\phi}}\newcommand{\bichi}{\boldsymbol{\chi}}\newcommand{\bipsi}{\boldsymbol{\psi}}\newcommand{\biomega}{\boldsymbol{\omega}}{\bf{c}}$ is a contrast stimulus (possibly corrupted by input noise), $\newcommand{\bialpha}{\boldsymbol{\alpha}}\newcommand{\bibeta}{\boldsymbol{\beta}}\newcommand{\bigamma}{\boldsymbol{\gamma}}\newcommand{\bidelta}{\boldsymbol{\delta}}\newcommand{\bivarepsilon}{\boldsymbol{\varepsilon}}\newcommand{\bizeta}{\boldsymbol{\zeta}}\newcommand{\bieta}{\boldsymbol{\eta}}\newcommand{\bitheta}{\boldsymbol{\theta}}\newcommand{\biiota}{\boldsymbol{\iota}}\newcommand{\bikappa}{\boldsymbol{\kappa}}\newcommand{\bilambda}{\boldsymbol{\lambda}}\newcommand{\bimu}{\boldsymbol{\mu}}\newcommand{\binu}{\boldsymbol{\nu}}\newcommand{\bixi}{\boldsymbol{\xi}}\newcommand{\biomicron}{\boldsymbol{\micron}}\newcommand{\bipi}{\boldsymbol{\pi}}\newcommand{\birho}{\boldsymbol{\rho}}\newcommand{\bisigma}{\boldsymbol{\sigma}}\newcommand{\bitau}{\boldsymbol{\tau}}\newcommand{\biupsilon}{\boldsymbol{\upsilon}}\newcommand{\biphi}{\boldsymbol{\phi}}\newcommand{\bichi}{\boldsymbol{\chi}}\newcommand{\bipsi}{\boldsymbol{\psi}}\newcommand{\biomega}{\boldsymbol{\omega}}N$ is the normalization factor, and $\newcommand{\bialpha}{\boldsymbol{\alpha}}\newcommand{\bibeta}{\boldsymbol{\beta}}\newcommand{\bigamma}{\boldsymbol{\gamma}}\newcommand{\bidelta}{\boldsymbol{\delta}}\newcommand{\bivarepsilon}{\boldsymbol{\varepsilon}}\newcommand{\bizeta}{\boldsymbol{\zeta}}\newcommand{\bieta}{\boldsymbol{\eta}}\newcommand{\bitheta}{\boldsymbol{\theta}}\newcommand{\biiota}{\boldsymbol{\iota}}\newcommand{\bikappa}{\boldsymbol{\kappa}}\newcommand{\bilambda}{\boldsymbol{\lambda}}\newcommand{\bimu}{\boldsymbol{\mu}}\newcommand{\binu}{\boldsymbol{\nu}}\newcommand{\bixi}{\boldsymbol{\xi}}\newcommand{\biomicron}{\boldsymbol{\micron}}\newcommand{\bipi}{\boldsymbol{\pi}}\newcommand{\birho}{\boldsymbol{\rho}}\newcommand{\bisigma}{\boldsymbol{\sigma}}\newcommand{\bitau}{\boldsymbol{\tau}}\newcommand{\biupsilon}{\boldsymbol{\upsilon}}\newcommand{\biphi}{\boldsymbol{\phi}}\newcommand{\bichi}{\boldsymbol{\chi}}\newcommand{\bipsi}{\boldsymbol{\psi}}\newcommand{\biomega}{\boldsymbol{\omega}}\varepsilon \sim N\left( {0,\sigma _I^2} \right)$ is encoding noise. The standard deviation $\newcommand{\bialpha}{\boldsymbol{\alpha}}\newcommand{\bibeta}{\boldsymbol{\beta}}\newcommand{\bigamma}{\boldsymbol{\gamma}}\newcommand{\bidelta}{\boldsymbol{\delta}}\newcommand{\bivarepsilon}{\boldsymbol{\varepsilon}}\newcommand{\bizeta}{\boldsymbol{\zeta}}\newcommand{\bieta}{\boldsymbol{\eta}}\newcommand{\bitheta}{\boldsymbol{\theta}}\newcommand{\biiota}{\boldsymbol{\iota}}\newcommand{\bikappa}{\boldsymbol{\kappa}}\newcommand{\bilambda}{\boldsymbol{\lambda}}\newcommand{\bimu}{\boldsymbol{\mu}}\newcommand{\binu}{\boldsymbol{\nu}}\newcommand{\bixi}{\boldsymbol{\xi}}\newcommand{\biomicron}{\boldsymbol{\micron}}\newcommand{\bipi}{\boldsymbol{\pi}}\newcommand{\birho}{\boldsymbol{\rho}}\newcommand{\bisigma}{\boldsymbol{\sigma}}\newcommand{\bitau}{\boldsymbol{\tau}}\newcommand{\biupsilon}{\boldsymbol{\upsilon}}\newcommand{\biphi}{\boldsymbol{\phi}}\newcommand{\bichi}{\boldsymbol{\chi}}\newcommand{\bipsi}{\boldsymbol{\psi}}\newcommand{\biomega}{\boldsymbol{\omega}}{\sigma _I}$ of the internal encoding noise can be constant, can scale in proportion to the mean response (i.e., Poisson-like), or can take other forms. Note that a Gaussian with variance equal to the mean provides a good approximation to the Poisson distribution for all but exceedingly low mean rates. The receptive field is assumed to have a vector magnitude (i.e., L2 norm) of 1.0.

The maximum response is set to a constant for all model neurons. In individual simple cells, the maximum firing rate is thought to be independent of preferred spatial frequency, orientation, and other stimulus preferences. It has been observed that the overall firing rate in cortex tends to decrease as spatial frequency increases. But this decrease in overall firing rate is likely to be a population effect due to a nonuniform distribution of spatial-frequency preferences in cortex, to sampling bias for low spatial frequencies in neuroscience studies, or both (De Valois, Albrecht, & Thorell, 1982; Foster, Gaska, Nagler, & Pollen, 1985; Victor, Purpura, Katz, & Mao, 1994). Thus, in the current article, and without loss of generality, for all receptive fields we assume $\newcommand{\bialpha}{\boldsymbol{\alpha}}\newcommand{\bibeta}{\boldsymbol{\beta}}\newcommand{\bigamma}{\boldsymbol{\gamma}}\newcommand{\bidelta}{\boldsymbol{\delta}}\newcommand{\bivarepsilon}{\boldsymbol{\varepsilon}}\newcommand{\bizeta}{\boldsymbol{\zeta}}\newcommand{\bieta}{\boldsymbol{\eta}}\newcommand{\bitheta}{\boldsymbol{\theta}}\newcommand{\biiota}{\boldsymbol{\iota}}\newcommand{\bikappa}{\boldsymbol{\kappa}}\newcommand{\bilambda}{\boldsymbol{\lambda}}\newcommand{\bimu}{\boldsymbol{\mu}}\newcommand{\binu}{\boldsymbol{\nu}}\newcommand{\bixi}{\boldsymbol{\xi}}\newcommand{\biomicron}{\boldsymbol{\micron}}\newcommand{\bipi}{\boldsymbol{\pi}}\newcommand{\birho}{\boldsymbol{\rho}}\newcommand{\bisigma}{\boldsymbol{\sigma}}\newcommand{\bitau}{\boldsymbol{\tau}}\newcommand{\biupsilon}{\boldsymbol{\upsilon}}\newcommand{\biphi}{\boldsymbol{\phi}}\newcommand{\bichi}{\boldsymbol{\chi}}\newcommand{\bipsi}{\boldsymbol{\psi}}\newcommand{\biomega}{\boldsymbol{\omega}}{r_{\max }}$ equals 1.0.

The response model in Equation 3 enforces the limited dynamic range of neurons in cortex and helps describe the shape of the contrast response functions of neurons in cortex (Figure 2B). The response model also accounts for the invariance of the shapes of orientation tuning curves to grating stimuli having different spatial frequencies (Figure 2C).

### Receptive field

**Figure 3:**
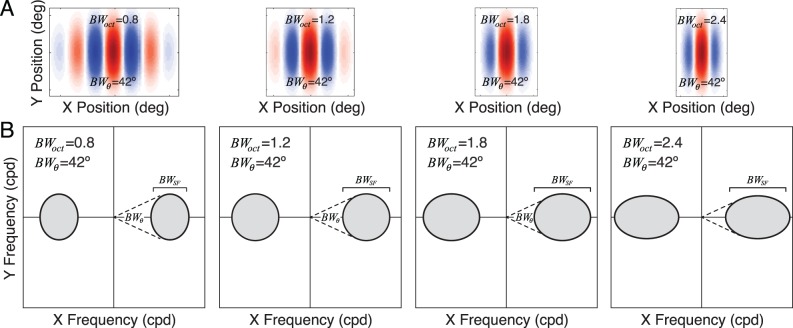
Gabor receptive fields and amplitude spectra. (A) Gabor receptive fields with octave bandwidths of 0.8, 1.2, 1.8, and 2.4, and orientation bandwidths of 42°. Different octave bandwidths correspond to preferred features with different aspect ratios (see Methods). (B) Amplitude spectra of Gabor receptive fields. Orientation bandwidth BW_θ_ is the polar angle spanned by the amplitude spectrum at half height. Spatial-frequency bandwidth BW_SF_ = f_hi_ − f_lo_ is the range of frequencies spanned by the spectrum, where f_hi_ and f_lo_ are the high and low frequencies at half height. Octave bandwidth BW_oct_ = log_2_(f_hi_/f_lo_) is the log-base-2 ratio of the frequencies.

A receptive field is a function that weights and sums inputs across space and time to determine a neuron's response. Responses increase when receptive-field locations having positive weights are stimulated with input increments and decrease when stimulated with input decrements. The opposite happens with locations having negative weights. In early visual cortex, simple-cell receptive fields are often modeled as having the shape of a Gabor—a cosine wave windowed by a Gaussian envelope (Jones & Palmer, 1987a, 1987b). Gabor receptive fields are orientation and spatial-frequency selective; the selectivity is commonly quantified by the bandwidth. The orientation bandwidth specifies the range of input orientations that can elicit a response. The median orientation bandwidth in cortex is 42° (De Valois, Yund, & Hepler, 1982). The spatial-frequency bandwidth specifies the range of input spatial frequencies that can elicit a response. The distribution of simple-cell bandwidths in cortex ranges between 0.8 and 2.4 octaves at half-height, with a median bandwidth of 1.5 octaves (De Valois, Albrecht, & Thorell, 1982; Ringach, 2002). We will characterize the response statistics of model neurons with vertically oriented Gabor receptive fields having the median orientation bandwidth of 42° and spatial-frequency bandwidths that span the same range as simple-cell receptive fields in cortex (octave bandwidths = 0.8, 1.2, 1.8, and 2.4; Figure 3A, 3B). We examine model-neuron responses having receptive fields with preferred spatial frequencies between 2 and 8 c/°.

Early models of neural response proposed that response drive is a linear function of the input stimulus (Campbell, Cleland, Cooper, & Enroth-Cugell, 1968; Hubel & Wiesel, 1962, 1968). The linear receptive-field responses $\newcommand{\bialpha}{\boldsymbol{\alpha}}\newcommand{\bibeta}{\boldsymbol{\beta}}\newcommand{\bigamma}{\boldsymbol{\gamma}}\newcommand{\bidelta}{\boldsymbol{\delta}}\newcommand{\bivarepsilon}{\boldsymbol{\varepsilon}}\newcommand{\bizeta}{\boldsymbol{\zeta}}\newcommand{\bieta}{\boldsymbol{\eta}}\newcommand{\bitheta}{\boldsymbol{\theta}}\newcommand{\biiota}{\boldsymbol{\iota}}\newcommand{\bikappa}{\boldsymbol{\kappa}}\newcommand{\bilambda}{\boldsymbol{\lambda}}\newcommand{\bimu}{\boldsymbol{\mu}}\newcommand{\binu}{\boldsymbol{\nu}}\newcommand{\bixi}{\boldsymbol{\xi}}\newcommand{\biomicron}{\boldsymbol{\micron}}\newcommand{\bipi}{\boldsymbol{\pi}}\newcommand{\birho}{\boldsymbol{\rho}}\newcommand{\bisigma}{\boldsymbol{\sigma}}\newcommand{\bitau}{\boldsymbol{\tau}}\newcommand{\biupsilon}{\boldsymbol{\upsilon}}\newcommand{\biphi}{\boldsymbol{\phi}}\newcommand{\bichi}{\boldsymbol{\chi}}\newcommand{\bipsi}{\boldsymbol{\psi}}\newcommand{\biomega}{\boldsymbol{\omega}}{R_{{{lin}}}} = {{\bf{f}}^{T}}{\bf{c}}$ to natural stimuli are nicely approximated by a generalized Gaussian with tails heavier than a Laplace distribution (Supplementary Figure S1). Previous analyses of linear responses have reported similar findings (Wainwright & Simoncelli, 2000). The linear model of response drive has been adopted by many computational communities; sparse coding is perhaps the best known among them (Olshausen & Field, 1996). We examine but do not focus on the linear responses, because real neurons include response normalization.

### Response normalization

The linear model explains neural responses in some regimes, but it is insufficiently rich to capture response properties over a wide range of stimulus conditions. Response normalization was originally proposed to account for the limited dynamic range of neurons in early visual cortex (Albrecht & Geisler, 1991; Heeger, 1992). Evidence for response normalization has been observed in primate retina, lateral geniculate nucleus, and early visual cortex (Albrecht & Geisler, 1991; Benardete, Kaplan, & Knight, 1992; Carandini, Heeger, & Movshon, 1997; Chander & Chichilnisky, 2001; Heeger, 1992; Mante, Bonin, & Carandini, 2008; Mante, Frazor, Bonin, Geisler, & Carandini, 2005; Nishimoto, Ishida, & Ohzawa, 2006; Shapley & Victor, 1978; Solomon, Peirce, Dhruv, & Lennie, 2004). In more recent years, normalization has been proposed to occur in higher cortical areas and to be associated with computations underlying diverse behavioral phenomena (Carandini & Heeger, 2012). We focus on how two types of response normalization—broadband and narrowband—impact response drives caused by natural stimuli.

Broadband normalization is stimulus specific but feature independent. With broadband normalization, the model-neuron responses are normalized by all the stimulus contrast in a local image region at the receptive-field location, regardless of its preferred feature (Carandini et al., 1997); all orientations and spatial frequencies normalize the linear response. The broadband normalization factor is

**Figure 4:**
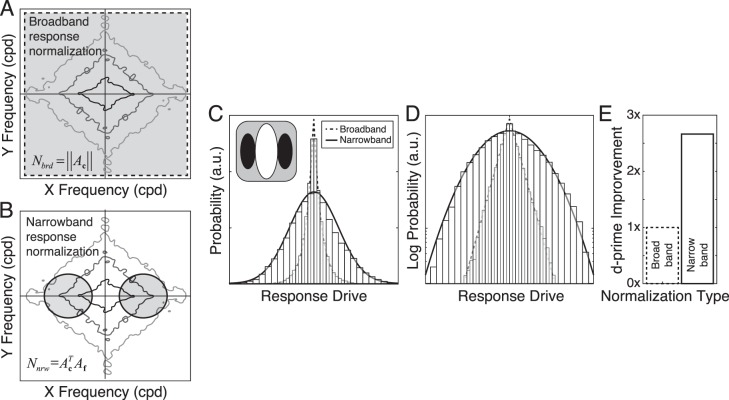
Broadband versus narrowband normalization with a Gabor-shaped receptive field. (A) Broadband normalization uses all the stimulus contrast (gray area) to normalize the linear receptive-field response, regardless of orientation and spatial frequency. The diamond-shaped contours represent the amplitude spectrum of an individual natural-image patch. (B) Narrowband normalization uses only the stimulus contrast in the passband of the receptive field (gray area) to normalize the linear receptive-field response. (C) Probability of model-neuron responses. With broadband normalization, receptive-field responses to natural stimuli are highly non-Gaussian, and are nicely approximated by a Laplace distribution (dashed curve). With narrowband normalization, the same receptive field yields responses to natural stimuli that are well described by a Gaussian (solid curve). (D) Same responses as in (C), but with the y-axis showing log-probability over three orders of magnitude. (E) Factor improvement in sensitivity (d′) for stimulus discriminability with narrowband versus broadband normalization. Results are shown for a vertically oriented Gabor with an orientation bandwidth of 42° and an octave bandwidth of 1.2. Similar results are obtained for other receptive fields.

\newcommand{\bialpha}{\boldsymbol{\alpha}}\newcommand{\bibeta}{\boldsymbol{\beta}}\newcommand{\bigamma}{\boldsymbol{\gamma}}\newcommand{\bidelta}{\boldsymbol{\delta}}\newcommand{\bivarepsilon}{\boldsymbol{\varepsilon}}\newcommand{\bizeta}{\boldsymbol{\zeta}}\newcommand{\bieta}{\boldsymbol{\eta}}\newcommand{\bitheta}{\boldsymbol{\theta}}\newcommand{\biiota}{\boldsymbol{\iota}}\newcommand{\bikappa}{\boldsymbol{\kappa}}\newcommand{\bilambda}{\boldsymbol{\lambda}}\newcommand{\bimu}{\boldsymbol{\mu}}\newcommand{\binu}{\boldsymbol{\nu}}\newcommand{\bixi}{\boldsymbol{\xi}}\newcommand{\biomicron}{\boldsymbol{\micron}}\newcommand{\bipi}{\boldsymbol{\pi}}\newcommand{\birho}{\boldsymbol{\rho}}\newcommand{\bisigma}{\boldsymbol{\sigma}}\newcommand{\bitau}{\boldsymbol{\tau}}\newcommand{\biupsilon}{\boldsymbol{\upsilon}}\newcommand{\biphi}{\boldsymbol{\phi}}\newcommand{\bichi}{\boldsymbol{\chi}}\newcommand{\bipsi}{\boldsymbol{\psi}}\newcommand{\biomega}{\boldsymbol{\omega}}\begin{equation}\tag{4}\eqalign{ {N_{{{brd}}}} = \left\| {{\bf{c}}} \right\| = \sqrt {\overbrace {\sum {{\bf{c}}_i^2} }^{\scriptstyle {\rm{stimulus}} \atop \scriptstyle {\rm{contrast\ energy}}} } \\ = \left\| {{A_{\bf{c}}}} \right\| = \sqrt {\overbrace {\sum {A_{{{\bf{c}}_i}}^2} }^{\scriptstyle {\rm{stimulus}} \atop \scriptstyle {\rm{contrast\ power}}} } \cr} {\rm ,}\end{equation}

where $\newcommand{\bialpha}{\boldsymbol{\alpha}}\newcommand{\bibeta}{\boldsymbol{\beta}}\newcommand{\bigamma}{\boldsymbol{\gamma}}\newcommand{\bidelta}{\boldsymbol{\delta}}\newcommand{\bivarepsilon}{\boldsymbol{\varepsilon}}\newcommand{\bizeta}{\boldsymbol{\zeta}}\newcommand{\bieta}{\boldsymbol{\eta}}\newcommand{\bitheta}{\boldsymbol{\theta}}\newcommand{\biiota}{\boldsymbol{\iota}}\newcommand{\bikappa}{\boldsymbol{\kappa}}\newcommand{\bilambda}{\boldsymbol{\lambda}}\newcommand{\bimu}{\boldsymbol{\mu}}\newcommand{\binu}{\boldsymbol{\nu}}\newcommand{\bixi}{\boldsymbol{\xi}}\newcommand{\biomicron}{\boldsymbol{\micron}}\newcommand{\bipi}{\boldsymbol{\pi}}\newcommand{\birho}{\boldsymbol{\rho}}\newcommand{\bisigma}{\boldsymbol{\sigma}}\newcommand{\bitau}{\boldsymbol{\tau}}\newcommand{\biupsilon}{\boldsymbol{\upsilon}}\newcommand{\biphi}{\boldsymbol{\phi}}\newcommand{\bichi}{\boldsymbol{\chi}}\newcommand{\bipsi}{\boldsymbol{\psi}}\newcommand{\biomega}{\boldsymbol{\omega}}{\bf{c}}$ is a (possibly noisy) Weber contrast stimulus, $\newcommand{\bialpha}{\boldsymbol{\alpha}}\newcommand{\bibeta}{\boldsymbol{\beta}}\newcommand{\bigamma}{\boldsymbol{\gamma}}\newcommand{\bidelta}{\boldsymbol{\delta}}\newcommand{\bivarepsilon}{\boldsymbol{\varepsilon}}\newcommand{\bizeta}{\boldsymbol{\zeta}}\newcommand{\bieta}{\boldsymbol{\eta}}\newcommand{\bitheta}{\boldsymbol{\theta}}\newcommand{\biiota}{\boldsymbol{\iota}}\newcommand{\bikappa}{\boldsymbol{\kappa}}\newcommand{\bilambda}{\boldsymbol{\lambda}}\newcommand{\bimu}{\boldsymbol{\mu}}\newcommand{\binu}{\boldsymbol{\nu}}\newcommand{\bixi}{\boldsymbol{\xi}}\newcommand{\biomicron}{\boldsymbol{\micron}}\newcommand{\bipi}{\boldsymbol{\pi}}\newcommand{\birho}{\boldsymbol{\rho}}\newcommand{\bisigma}{\boldsymbol{\sigma}}\newcommand{\bitau}{\boldsymbol{\tau}}\newcommand{\biupsilon}{\boldsymbol{\upsilon}}\newcommand{\biphi}{\boldsymbol{\phi}}\newcommand{\bichi}{\boldsymbol{\chi}}\newcommand{\bipsi}{\boldsymbol{\psi}}\newcommand{\biomega}{\boldsymbol{\omega}}{A_{\bf{c}}}$ is the amplitude spectrum of the contrast stimulus, and the L2 norm operator $\newcommand{\bialpha}{\boldsymbol{\alpha}}\newcommand{\bibeta}{\boldsymbol{\beta}}\newcommand{\bigamma}{\boldsymbol{\gamma}}\newcommand{\bidelta}{\boldsymbol{\delta}}\newcommand{\bivarepsilon}{\boldsymbol{\varepsilon}}\newcommand{\bizeta}{\boldsymbol{\zeta}}\newcommand{\bieta}{\boldsymbol{\eta}}\newcommand{\bitheta}{\boldsymbol{\theta}}\newcommand{\biiota}{\boldsymbol{\iota}}\newcommand{\bikappa}{\boldsymbol{\kappa}}\newcommand{\bilambda}{\boldsymbol{\lambda}}\newcommand{\bimu}{\boldsymbol{\mu}}\newcommand{\binu}{\boldsymbol{\nu}}\newcommand{\bixi}{\boldsymbol{\xi}}\newcommand{\biomicron}{\boldsymbol{\micron}}\newcommand{\bipi}{\boldsymbol{\pi}}\newcommand{\birho}{\boldsymbol{\rho}}\newcommand{\bisigma}{\boldsymbol{\sigma}}\newcommand{\bitau}{\boldsymbol{\tau}}\newcommand{\biupsilon}{\boldsymbol{\upsilon}}\newcommand{\biphi}{\boldsymbol{\phi}}\newcommand{\bichi}{\boldsymbol{\chi}}\newcommand{\bipsi}{\boldsymbol{\psi}}\newcommand{\biomega}{\boldsymbol{\omega}}\left\| { \cdot } \right\|$ gives the square root of the sum of squares. Parseval's theorem guarantees that the total energy of the contrast stimulus equals the total power of its amplitude spectrum (Figure 4A). Note that if the contrast stimulus is noisy (e.g., corrupted by pixel noise), the broadband normalization factor equals $\newcommand{\bialpha}{\boldsymbol{\alpha}}\newcommand{\bibeta}{\boldsymbol{\beta}}\newcommand{\bigamma}{\boldsymbol{\gamma}}\newcommand{\bidelta}{\boldsymbol{\delta}}\newcommand{\bivarepsilon}{\boldsymbol{\varepsilon}}\newcommand{\bizeta}{\boldsymbol{\zeta}}\newcommand{\bieta}{\boldsymbol{\eta}}\newcommand{\bitheta}{\boldsymbol{\theta}}\newcommand{\biiota}{\boldsymbol{\iota}}\newcommand{\bikappa}{\boldsymbol{\kappa}}\newcommand{\bilambda}{\boldsymbol{\lambda}}\newcommand{\bimu}{\boldsymbol{\mu}}\newcommand{\binu}{\boldsymbol{\nu}}\newcommand{\bixi}{\boldsymbol{\xi}}\newcommand{\biomicron}{\boldsymbol{\micron}}\newcommand{\bipi}{\boldsymbol{\pi}}\newcommand{\birho}{\boldsymbol{\rho}}\newcommand{\bisigma}{\boldsymbol{\sigma}}\newcommand{\bitau}{\boldsymbol{\tau}}\newcommand{\biupsilon}{\boldsymbol{\upsilon}}\newcommand{\biphi}{\boldsymbol{\phi}}\newcommand{\bichi}{\boldsymbol{\chi}}\newcommand{\bipsi}{\boldsymbol{\psi}}\newcommand{\biomega}{\boldsymbol{\omega}}{N_{{{brd}}}} = \sqrt {\sum {{\bf{c}}_i^2 + \sigma _i^2} }$ to a very close approximation, where $\newcommand{\bialpha}{\boldsymbol{\alpha}}\newcommand{\bibeta}{\boldsymbol{\beta}}\newcommand{\bigamma}{\boldsymbol{\gamma}}\newcommand{\bidelta}{\boldsymbol{\delta}}\newcommand{\bivarepsilon}{\boldsymbol{\varepsilon}}\newcommand{\bizeta}{\boldsymbol{\zeta}}\newcommand{\bieta}{\boldsymbol{\eta}}\newcommand{\bitheta}{\boldsymbol{\theta}}\newcommand{\biiota}{\boldsymbol{\iota}}\newcommand{\bikappa}{\boldsymbol{\kappa}}\newcommand{\bilambda}{\boldsymbol{\lambda}}\newcommand{\bimu}{\boldsymbol{\mu}}\newcommand{\binu}{\boldsymbol{\nu}}\newcommand{\bixi}{\boldsymbol{\xi}}\newcommand{\biomicron}{\boldsymbol{\micron}}\newcommand{\bipi}{\boldsymbol{\pi}}\newcommand{\birho}{\boldsymbol{\rho}}\newcommand{\bisigma}{\boldsymbol{\sigma}}\newcommand{\bitau}{\boldsymbol{\tau}}\newcommand{\biupsilon}{\boldsymbol{\upsilon}}\newcommand{\biphi}{\boldsymbol{\phi}}\newcommand{\bichi}{\boldsymbol{\chi}}\newcommand{\bipsi}{\boldsymbol{\psi}}\newcommand{\biomega}{\boldsymbol{\omega}}\sigma$ is the standard deviation of the input noise (Burge & Geisler, 2014).

Narrowband normalization is stimulus specific and feature dependent. With narrowband normalization, the model-neuron responses are normalized by the stimulus contrast in the passband of the receptive field, which means that the stimulus features that contribute most prominently are those that approximately match the preferred feature (Figure 4B). For example, if the receptive field's preferred stimulus is a vertically oriented Gabor with a carrier frequency of 4 c/°, the responses are normalized primarily by features that are near vertical and near to 4 c/° (Ruff et al., 2016). The narrowband normalization factor is given by

\newcommand{\bialpha}{\boldsymbol{\alpha}}\newcommand{\bibeta}{\boldsymbol{\beta}}\newcommand{\bigamma}{\boldsymbol{\gamma}}\newcommand{\bidelta}{\boldsymbol{\delta}}\newcommand{\bivarepsilon}{\boldsymbol{\varepsilon}}\newcommand{\bizeta}{\boldsymbol{\zeta}}\newcommand{\bieta}{\boldsymbol{\eta}}\newcommand{\bitheta}{\boldsymbol{\theta}}\newcommand{\biiota}{\boldsymbol{\iota}}\newcommand{\bikappa}{\boldsymbol{\kappa}}\newcommand{\bilambda}{\boldsymbol{\lambda}}\newcommand{\bimu}{\boldsymbol{\mu}}\newcommand{\binu}{\boldsymbol{\nu}}\newcommand{\bixi}{\boldsymbol{\xi}}\newcommand{\biomicron}{\boldsymbol{\micron}}\newcommand{\bipi}{\boldsymbol{\pi}}\newcommand{\birho}{\boldsymbol{\rho}}\newcommand{\bisigma}{\boldsymbol{\sigma}}\newcommand{\bitau}{\boldsymbol{\tau}}\newcommand{\biupsilon}{\boldsymbol{\upsilon}}\newcommand{\biphi}{\boldsymbol{\phi}}\newcommand{\bichi}{\boldsymbol{\chi}}\newcommand{\bipsi}{\boldsymbol{\psi}}\newcommand{\biomega}{\boldsymbol{\omega}}\begin{equation}\tag{5}{ {N_{{{nrw}}}} = {N_{{{brd}}}}S \\ = A_{\bf{c}}^{T}A_{\bf{f}}^{} } {\rm ,}\end{equation}

where $\newcommand{\bialpha}{\boldsymbol{\alpha}}\newcommand{\bibeta}{\boldsymbol{\beta}}\newcommand{\bigamma}{\boldsymbol{\gamma}}\newcommand{\bidelta}{\boldsymbol{\delta}}\newcommand{\bivarepsilon}{\boldsymbol{\varepsilon}}\newcommand{\bizeta}{\boldsymbol{\zeta}}\newcommand{\bieta}{\boldsymbol{\eta}}\newcommand{\bitheta}{\boldsymbol{\theta}}\newcommand{\biiota}{\boldsymbol{\iota}}\newcommand{\bikappa}{\boldsymbol{\kappa}}\newcommand{\bilambda}{\boldsymbol{\lambda}}\newcommand{\bimu}{\boldsymbol{\mu}}\newcommand{\binu}{\boldsymbol{\nu}}\newcommand{\bixi}{\boldsymbol{\xi}}\newcommand{\biomicron}{\boldsymbol{\micron}}\newcommand{\bipi}{\boldsymbol{\pi}}\newcommand{\birho}{\boldsymbol{\rho}}\newcommand{\bisigma}{\boldsymbol{\sigma}}\newcommand{\bitau}{\boldsymbol{\tau}}\newcommand{\biupsilon}{\boldsymbol{\upsilon}}\newcommand{\biphi}{\boldsymbol{\phi}}\newcommand{\bichi}{\boldsymbol{\chi}}\newcommand{\bipsi}{\boldsymbol{\psi}}\newcommand{\biomega}{\boldsymbol{\omega}}S = {{{A_{\bf{c}}^{T}}{A_{\bf{f}}}} \over {\left\| {{A_{\bf{c}}}} \right\|\left\| {{A_{\bf{f}}}} \right\|}}$ is the phase-invariant similarity, the cosine similarity between the stimulus and receptive-field amplitude spectra (Sebastian et al., 2017). (The amplitude spectrum of the receptive field $\newcommand{\bialpha}{\boldsymbol{\alpha}}\newcommand{\bibeta}{\boldsymbol{\beta}}\newcommand{\bigamma}{\boldsymbol{\gamma}}\newcommand{\bidelta}{\boldsymbol{\delta}}\newcommand{\bivarepsilon}{\boldsymbol{\varepsilon}}\newcommand{\bizeta}{\boldsymbol{\zeta}}\newcommand{\bieta}{\boldsymbol{\eta}}\newcommand{\bitheta}{\boldsymbol{\theta}}\newcommand{\biiota}{\boldsymbol{\iota}}\newcommand{\bikappa}{\boldsymbol{\kappa}}\newcommand{\bilambda}{\boldsymbol{\lambda}}\newcommand{\bimu}{\boldsymbol{\mu}}\newcommand{\binu}{\boldsymbol{\nu}}\newcommand{\bixi}{\boldsymbol{\xi}}\newcommand{\biomicron}{\boldsymbol{\micron}}\newcommand{\bipi}{\boldsymbol{\pi}}\newcommand{\birho}{\boldsymbol{\rho}}\newcommand{\bisigma}{\boldsymbol{\sigma}}\newcommand{\bitau}{\boldsymbol{\tau}}\newcommand{\biupsilon}{\boldsymbol{\upsilon}}\newcommand{\biphi}{\boldsymbol{\phi}}\newcommand{\bichi}{\boldsymbol{\chi}}\newcommand{\bipsi}{\boldsymbol{\psi}}\newcommand{\biomega}{\boldsymbol{\omega}}{A_{\bf{f}}}$ is assumed to have an L2 norm of 1.0.) Similarity is thus constrained to take a value between 0 and 1, which means that the narrowband normalization factor is always less than or equal to the broadband factor.

Broadband-normalized response drives $\newcommand{\bialpha}{\boldsymbol{\alpha}}\newcommand{\bibeta}{\boldsymbol{\beta}}\newcommand{\bigamma}{\boldsymbol{\gamma}}\newcommand{\bidelta}{\boldsymbol{\delta}}\newcommand{\bivarepsilon}{\boldsymbol{\varepsilon}}\newcommand{\bizeta}{\boldsymbol{\zeta}}\newcommand{\bieta}{\boldsymbol{\eta}}\newcommand{\bitheta}{\boldsymbol{\theta}}\newcommand{\biiota}{\boldsymbol{\iota}}\newcommand{\bikappa}{\boldsymbol{\kappa}}\newcommand{\bilambda}{\boldsymbol{\lambda}}\newcommand{\bimu}{\boldsymbol{\mu}}\newcommand{\binu}{\boldsymbol{\nu}}\newcommand{\bixi}{\boldsymbol{\xi}}\newcommand{\biomicron}{\boldsymbol{\micron}}\newcommand{\bipi}{\boldsymbol{\pi}}\newcommand{\birho}{\boldsymbol{\rho}}\newcommand{\bisigma}{\boldsymbol{\sigma}}\newcommand{\bitau}{\boldsymbol{\tau}}\newcommand{\biupsilon}{\boldsymbol{\upsilon}}\newcommand{\biphi}{\boldsymbol{\phi}}\newcommand{\bichi}{\boldsymbol{\chi}}\newcommand{\bipsi}{\boldsymbol{\psi}}\newcommand{\biomega}{\boldsymbol{\omega}}{R_{{{brd}}}} \propto {{{{\bf{f}}^{T}}{\bf{c}}} \mathord{\left/ {\vphantom {{{{\bf{f}}^{T}}{\bf{c}}} {{N_{{{brd}}}}}}} \right. \kern-1.2pt} {{N_{{{brd}}}}}}$ to natural stimuli are highly non-Gaussian. The Laplace distribution provides an excellent fit to the broadband responses for all preferred spatial frequencies and octave bandwidths (Figure 4C, 4D; Supplementary Figure S2). Narrowband-normalized response drives $\newcommand{\bialpha}{\boldsymbol{\alpha}}\newcommand{\bibeta}{\boldsymbol{\beta}}\newcommand{\bigamma}{\boldsymbol{\gamma}}\newcommand{\bidelta}{\boldsymbol{\delta}}\newcommand{\bivarepsilon}{\boldsymbol{\varepsilon}}\newcommand{\bizeta}{\boldsymbol{\zeta}}\newcommand{\bieta}{\boldsymbol{\eta}}\newcommand{\bitheta}{\boldsymbol{\theta}}\newcommand{\biiota}{\boldsymbol{\iota}}\newcommand{\bikappa}{\boldsymbol{\kappa}}\newcommand{\bilambda}{\boldsymbol{\lambda}}\newcommand{\bimu}{\boldsymbol{\mu}}\newcommand{\binu}{\boldsymbol{\nu}}\newcommand{\bixi}{\boldsymbol{\xi}}\newcommand{\biomicron}{\boldsymbol{\micron}}\newcommand{\bipi}{\boldsymbol{\pi}}\newcommand{\birho}{\boldsymbol{\rho}}\newcommand{\bisigma}{\boldsymbol{\sigma}}\newcommand{\bitau}{\boldsymbol{\tau}}\newcommand{\biupsilon}{\boldsymbol{\upsilon}}\newcommand{\biphi}{\boldsymbol{\phi}}\newcommand{\bichi}{\boldsymbol{\chi}}\newcommand{\bipsi}{\boldsymbol{\psi}}\newcommand{\biomega}{\boldsymbol{\omega}}{R_{{{nrw}}}} \propto {{{{\bf{f}}^{T}}{\bf{c}}} \mathord{\left/ {\vphantom {{{{\bf{f}}^{T}}{\bf{c}}} {{N_{{\rm{nrw}}}}}}} \right. \kern-1.2pt} {{N_{{{nrw}}}}}}$ differ in two important respects. First, the standard deviation of the natural-stimulus drive $\newcommand{\bialpha}{\boldsymbol{\alpha}}\newcommand{\bibeta}{\boldsymbol{\beta}}\newcommand{\bigamma}{\boldsymbol{\gamma}}\newcommand{\bidelta}{\boldsymbol{\delta}}\newcommand{\bivarepsilon}{\boldsymbol{\varepsilon}}\newcommand{\bizeta}{\boldsymbol{\zeta}}\newcommand{\bieta}{\boldsymbol{\eta}}\newcommand{\bitheta}{\boldsymbol{\theta}}\newcommand{\biiota}{\boldsymbol{\iota}}\newcommand{\bikappa}{\boldsymbol{\kappa}}\newcommand{\bilambda}{\boldsymbol{\lambda}}\newcommand{\bimu}{\boldsymbol{\mu}}\newcommand{\binu}{\boldsymbol{\nu}}\newcommand{\bixi}{\boldsymbol{\xi}}\newcommand{\biomicron}{\boldsymbol{\micron}}\newcommand{\bipi}{\boldsymbol{\pi}}\newcommand{\birho}{\boldsymbol{\rho}}\newcommand{\bisigma}{\boldsymbol{\sigma}}\newcommand{\bitau}{\boldsymbol{\tau}}\newcommand{\biupsilon}{\boldsymbol{\upsilon}}\newcommand{\biphi}{\boldsymbol{\phi}}\newcommand{\bichi}{\boldsymbol{\chi}}\newcommand{\bipsi}{\boldsymbol{\psi}}\newcommand{\biomega}{\boldsymbol{\omega}}{\sigma _E}$ is approximately two and a half times higher for narrowband than broadband responses. Second, narrowband normalization yields distributions of response drive that are approximately Gaussian (Figure 4C, 4D; Supplementary Figure S3). Related findings have been reported by other groups (Burge & Geisler, 2014, 2015; Jaini & Burge, 2017; Lyu & Simoncelli, 2008, 2009a; Sebastian et al., 2017; Wainwright & Simoncelli, 2000).

Relative to broadband normalization, narrowband normalization improves sensitivity for stimulus discrimination by nearly three times, assuming constant encoding noise (Figure 4E). The improvement in sensitivity is mediated both by increased Gaussianity (Supplementary Figure S4A) and by the increased stimulus-driven response variability (Equation 2). Poisson-like or modulated Poisson-like encoding noise, which is more like response noise in cortex (Goris, Ziemba, Movshon, & Simoncelli, 2018; Tolhurst, Movshon, & Dean, 1983), yields similar results (Supplementary Figure S4B through S4D).

**Figure 5:**
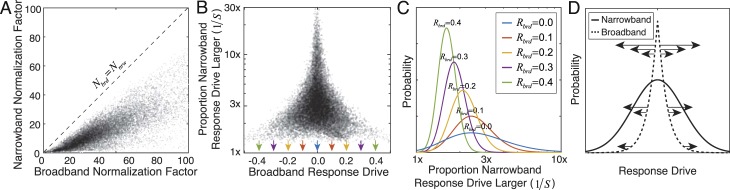
Broadband versus narrowband normalization. (A) The narrowband normalization factor is smaller than the broadband normalization factor for each stimulus. (B) The proportion by which the narrowband response is larger than the broadband response R_nrw_/R_brd_ as a function of the broadband response. Large proportions occur only for small broadband responses, accounting for why narrowband normalization increases the Gaussianity of the model-neuron responses. (C) Distribution of the proportional increase in the narrowband response relative to the broadband response, conditioned on different absolute values of the broadband response (colors), as fitted by inverse gamma distributions (Supplementary Figure S5). The proportion is equivalent to inverse similarity. Arrows in (B) mark the absolute values of broadband response upon which the proportions are conditioned. (D) Schematic showing why the relationship between narrowband and broadband response drives contributes to the increased Gaussianity of the model-neuron responses. The data in (A–C) are for a vertically oriented cosine-phase Gabor receptive field with an orientation bandwidth of 42°, an octave bandwidth of 1.2, and a preferred frequency of 2 c/°. Results are similar for all receptive fields.

Why does narrowband normalization increase stimulus-driven variance relative to broadband normalization? Because the narrowband normalization factor is always less than or equal to the broadband normalization factor (Figure 5A; Equations 4 and 5). Therefore, across many stimuli, the distribution of response drive will tend to have larger variance with narrowband normalization. How does narrowband normalization cause more Gaussian distributions of response drive than broadband normalization? Narrowband normalization amplifies small broadband response drives, and leaves large broadband response drives relatively unperturbed (Figure 5B). For example, if the stimulus is a poor match to the receptive field (i.e., the broadband response approaches 0.0), it is likely that only a small proportion of the stimulus contrast is in the pass band of the receptive field. This, in turn, means that the narrowband normalization factor will be quite small compared to the broadband factor, which will increase the proportion by which the narrowband response drive is amplified (Equation 5; Figure 5B, 5C). On the other extreme, if the stimulus is a perfect match to the receptive field, the broadband response drive equals $\newcommand{\bialpha}{\boldsymbol{\alpha}}\newcommand{\bibeta}{\boldsymbol{\beta}}\newcommand{\bigamma}{\boldsymbol{\gamma}}\newcommand{\bidelta}{\boldsymbol{\delta}}\newcommand{\bivarepsilon}{\boldsymbol{\varepsilon}}\newcommand{\bizeta}{\boldsymbol{\zeta}}\newcommand{\bieta}{\boldsymbol{\eta}}\newcommand{\bitheta}{\boldsymbol{\theta}}\newcommand{\biiota}{\boldsymbol{\iota}}\newcommand{\bikappa}{\boldsymbol{\kappa}}\newcommand{\bilambda}{\boldsymbol{\lambda}}\newcommand{\bimu}{\boldsymbol{\mu}}\newcommand{\binu}{\boldsymbol{\nu}}\newcommand{\bixi}{\boldsymbol{\xi}}\newcommand{\biomicron}{\boldsymbol{\micron}}\newcommand{\bipi}{\boldsymbol{\pi}}\newcommand{\birho}{\boldsymbol{\rho}}\newcommand{\bisigma}{\boldsymbol{\sigma}}\newcommand{\bitau}{\boldsymbol{\tau}}\newcommand{\biupsilon}{\boldsymbol{\upsilon}}\newcommand{\biphi}{\boldsymbol{\phi}}\newcommand{\bichi}{\boldsymbol{\chi}}\newcommand{\bipsi}{\boldsymbol{\psi}}\newcommand{\biomega}{\boldsymbol{\omega}}{r_{\max }}$, and all the stimulus contrast must be in the pass band of the receptive field. In this situation, the narrowband and broadband normalization factors will be identical, and the narrowband response will equal the broadband response (Figure 5C). These effects mediate the differences in the shapes of the broadband and narrowband response distributions (Figure 5D).

The proportional increase $\newcommand{\bialpha}{\boldsymbol{\alpha}}\newcommand{\bibeta}{\boldsymbol{\beta}}\newcommand{\bigamma}{\boldsymbol{\gamma}}\newcommand{\bidelta}{\boldsymbol{\delta}}\newcommand{\bivarepsilon}{\boldsymbol{\varepsilon}}\newcommand{\bizeta}{\boldsymbol{\zeta}}\newcommand{\bieta}{\boldsymbol{\eta}}\newcommand{\bitheta}{\boldsymbol{\theta}}\newcommand{\biiota}{\boldsymbol{\iota}}\newcommand{\bikappa}{\boldsymbol{\kappa}}\newcommand{\bilambda}{\boldsymbol{\lambda}}\newcommand{\bimu}{\boldsymbol{\mu}}\newcommand{\binu}{\boldsymbol{\nu}}\newcommand{\bixi}{\boldsymbol{\xi}}\newcommand{\biomicron}{\boldsymbol{\micron}}\newcommand{\bipi}{\boldsymbol{\pi}}\newcommand{\birho}{\boldsymbol{\rho}}\newcommand{\bisigma}{\boldsymbol{\sigma}}\newcommand{\bitau}{\boldsymbol{\tau}}\newcommand{\biupsilon}{\boldsymbol{\upsilon}}\newcommand{\biphi}{\boldsymbol{\phi}}\newcommand{\bichi}{\boldsymbol{\chi}}\newcommand{\bipsi}{\boldsymbol{\psi}}\newcommand{\biomega}{\boldsymbol{\omega}}{{{R_{{{nrw}}}}} \mathord{\left/ {\vphantom {{{R_{{{nrw}}}}} {{R_{{{brd}}}}}}} \right. \kern-1.2pt} {{R_{{{brd}}}}}}$ of the narrowband versus the broadband response drive depends strongly on the broadband response (Figure 5B). Figure 5C shows conditional distributions of proportional increase for five different absolute values of the broadband responses, as fitted by inverse gamma distributions (Supplementary Figure S5). When stimuli are narrowband normalized, small broadband responses are amplified more than large ones.

There is an additional point worth making. The results presented in Figure 4C and 4D suggest that the broadband response drives can be represented as a Gaussian scale mixture of random variables. In considering natural images, the input contrast image $\newcommand{\bialpha}{\boldsymbol{\alpha}}\newcommand{\bibeta}{\boldsymbol{\beta}}\newcommand{\bigamma}{\boldsymbol{\gamma}}\newcommand{\bidelta}{\boldsymbol{\delta}}\newcommand{\bivarepsilon}{\boldsymbol{\varepsilon}}\newcommand{\bizeta}{\boldsymbol{\zeta}}\newcommand{\bieta}{\boldsymbol{\eta}}\newcommand{\bitheta}{\boldsymbol{\theta}}\newcommand{\biiota}{\boldsymbol{\iota}}\newcommand{\bikappa}{\boldsymbol{\kappa}}\newcommand{\bilambda}{\boldsymbol{\lambda}}\newcommand{\bimu}{\boldsymbol{\mu}}\newcommand{\binu}{\boldsymbol{\nu}}\newcommand{\bixi}{\boldsymbol{\xi}}\newcommand{\biomicron}{\boldsymbol{\micron}}\newcommand{\bipi}{\boldsymbol{\pi}}\newcommand{\birho}{\boldsymbol{\rho}}\newcommand{\bisigma}{\boldsymbol{\sigma}}\newcommand{\bitau}{\boldsymbol{\tau}}\newcommand{\biupsilon}{\boldsymbol{\upsilon}}\newcommand{\biphi}{\boldsymbol{\phi}}\newcommand{\bichi}{\boldsymbol{\chi}}\newcommand{\bipsi}{\boldsymbol{\psi}}\newcommand{\biomega}{\boldsymbol{\omega}}{\bf{c}}$ is a random variable. It follows that the broadband response drive $\newcommand{\bialpha}{\boldsymbol{\alpha}}\newcommand{\bibeta}{\boldsymbol{\beta}}\newcommand{\bigamma}{\boldsymbol{\gamma}}\newcommand{\bidelta}{\boldsymbol{\delta}}\newcommand{\bivarepsilon}{\boldsymbol{\varepsilon}}\newcommand{\bizeta}{\boldsymbol{\zeta}}\newcommand{\bieta}{\boldsymbol{\eta}}\newcommand{\bitheta}{\boldsymbol{\theta}}\newcommand{\biiota}{\boldsymbol{\iota}}\newcommand{\bikappa}{\boldsymbol{\kappa}}\newcommand{\bilambda}{\boldsymbol{\lambda}}\newcommand{\bimu}{\boldsymbol{\mu}}\newcommand{\binu}{\boldsymbol{\nu}}\newcommand{\bixi}{\boldsymbol{\xi}}\newcommand{\biomicron}{\boldsymbol{\micron}}\newcommand{\bipi}{\boldsymbol{\pi}}\newcommand{\birho}{\boldsymbol{\rho}}\newcommand{\bisigma}{\boldsymbol{\sigma}}\newcommand{\bitau}{\boldsymbol{\tau}}\newcommand{\biupsilon}{\boldsymbol{\upsilon}}\newcommand{\biphi}{\boldsymbol{\phi}}\newcommand{\bichi}{\boldsymbol{\chi}}\newcommand{\bipsi}{\boldsymbol{\psi}}\newcommand{\biomega}{\boldsymbol{\omega}}{R_{{{brd}}}}$, the narrowband response $\newcommand{\bialpha}{\boldsymbol{\alpha}}\newcommand{\bibeta}{\boldsymbol{\beta}}\newcommand{\bigamma}{\boldsymbol{\gamma}}\newcommand{\bidelta}{\boldsymbol{\delta}}\newcommand{\bivarepsilon}{\boldsymbol{\varepsilon}}\newcommand{\bizeta}{\boldsymbol{\zeta}}\newcommand{\bieta}{\boldsymbol{\eta}}\newcommand{\bitheta}{\boldsymbol{\theta}}\newcommand{\biiota}{\boldsymbol{\iota}}\newcommand{\bikappa}{\boldsymbol{\kappa}}\newcommand{\bilambda}{\boldsymbol{\lambda}}\newcommand{\bimu}{\boldsymbol{\mu}}\newcommand{\binu}{\boldsymbol{\nu}}\newcommand{\bixi}{\boldsymbol{\xi}}\newcommand{\biomicron}{\boldsymbol{\micron}}\newcommand{\bipi}{\boldsymbol{\pi}}\newcommand{\birho}{\boldsymbol{\rho}}\newcommand{\bisigma}{\boldsymbol{\sigma}}\newcommand{\bitau}{\boldsymbol{\tau}}\newcommand{\biupsilon}{\boldsymbol{\upsilon}}\newcommand{\biphi}{\boldsymbol{\phi}}\newcommand{\bichi}{\boldsymbol{\chi}}\newcommand{\bipsi}{\boldsymbol{\psi}}\newcommand{\biomega}{\boldsymbol{\omega}}{R_{{{nrw}}}}$, and the phase-invariant similarity $\newcommand{\bialpha}{\boldsymbol{\alpha}}\newcommand{\bibeta}{\boldsymbol{\beta}}\newcommand{\bigamma}{\boldsymbol{\gamma}}\newcommand{\bidelta}{\boldsymbol{\delta}}\newcommand{\bivarepsilon}{\boldsymbol{\varepsilon}}\newcommand{\bizeta}{\boldsymbol{\zeta}}\newcommand{\bieta}{\boldsymbol{\eta}}\newcommand{\bitheta}{\boldsymbol{\theta}}\newcommand{\biiota}{\boldsymbol{\iota}}\newcommand{\bikappa}{\boldsymbol{\kappa}}\newcommand{\bilambda}{\boldsymbol{\lambda}}\newcommand{\bimu}{\boldsymbol{\mu}}\newcommand{\binu}{\boldsymbol{\nu}}\newcommand{\bixi}{\boldsymbol{\xi}}\newcommand{\biomicron}{\boldsymbol{\micron}}\newcommand{\bipi}{\boldsymbol{\pi}}\newcommand{\birho}{\boldsymbol{\rho}}\newcommand{\bisigma}{\boldsymbol{\sigma}}\newcommand{\bitau}{\boldsymbol{\tau}}\newcommand{\biupsilon}{\boldsymbol{\upsilon}}\newcommand{\biphi}{\boldsymbol{\phi}}\newcommand{\bichi}{\boldsymbol{\chi}}\newcommand{\bipsi}{\boldsymbol{\psi}}\newcommand{\biomega}{\boldsymbol{\omega}}S$ are all random variables. By combining Equations 4 and 5, it is easy to show that these variables have the following relationships:

\newcommand{\bialpha}{\boldsymbol{\alpha}}\newcommand{\bibeta}{\boldsymbol{\beta}}\newcommand{\bigamma}{\boldsymbol{\gamma}}\newcommand{\bidelta}{\boldsymbol{\delta}}\newcommand{\bivarepsilon}{\boldsymbol{\varepsilon}}\newcommand{\bizeta}{\boldsymbol{\zeta}}\newcommand{\bieta}{\boldsymbol{\eta}}\newcommand{\bitheta}{\boldsymbol{\theta}}\newcommand{\biiota}{\boldsymbol{\iota}}\newcommand{\bikappa}{\boldsymbol{\kappa}}\newcommand{\bilambda}{\boldsymbol{\lambda}}\newcommand{\bimu}{\boldsymbol{\mu}}\newcommand{\binu}{\boldsymbol{\nu}}\newcommand{\bixi}{\boldsymbol{\xi}}\newcommand{\biomicron}{\boldsymbol{\micron}}\newcommand{\bipi}{\boldsymbol{\pi}}\newcommand{\birho}{\boldsymbol{\rho}}\newcommand{\bisigma}{\boldsymbol{\sigma}}\newcommand{\bitau}{\boldsymbol{\tau}}\newcommand{\biupsilon}{\boldsymbol{\upsilon}}\newcommand{\biphi}{\boldsymbol{\phi}}\newcommand{\bichi}{\boldsymbol{\chi}}\newcommand{\bipsi}{\boldsymbol{\psi}}\newcommand{\biomega}{\boldsymbol{\omega}}\begin{equation}\tag{6A}R_{{brd}} = {{\bf f}^T {\bf c} \over N_{{brd}}} = {{\bf f}^T {\bf c} \over N_{{nrw}}}S = {R_{{{nrw}}}}S \end{equation}

\newcommand{\bialpha}{\boldsymbol{\alpha}}\newcommand{\bibeta}{\boldsymbol{\beta}}\newcommand{\bigamma}{\boldsymbol{\gamma}}\newcommand{\bidelta}{\boldsymbol{\delta}}\newcommand{\bivarepsilon}{\boldsymbol{\varepsilon}}\newcommand{\bizeta}{\boldsymbol{\zeta}}\newcommand{\bieta}{\boldsymbol{\eta}}\newcommand{\bitheta}{\boldsymbol{\theta}}\newcommand{\biiota}{\boldsymbol{\iota}}\newcommand{\bikappa}{\boldsymbol{\kappa}}\newcommand{\bilambda}{\boldsymbol{\lambda}}\newcommand{\bimu}{\boldsymbol{\mu}}\newcommand{\binu}{\boldsymbol{\nu}}\newcommand{\bixi}{\boldsymbol{\xi}}\newcommand{\biomicron}{\boldsymbol{\micron}}\newcommand{\bipi}{\boldsymbol{\pi}}\newcommand{\birho}{\boldsymbol{\rho}}\newcommand{\bisigma}{\boldsymbol{\sigma}}\newcommand{\bitau}{\boldsymbol{\tau}}\newcommand{\biupsilon}{\boldsymbol{\upsilon}}\newcommand{\biphi}{\boldsymbol{\phi}}\newcommand{\bichi}{\boldsymbol{\chi}}\newcommand{\bipsi}{\boldsymbol{\psi}}\newcommand{\biomega}{\boldsymbol{\omega}}\begin{equation}\tag{6B}{R_{{{brd}}}} = {R_{{{nrw}}}}\sqrt {{S^2}} \end{equation}

**Figure 6:**
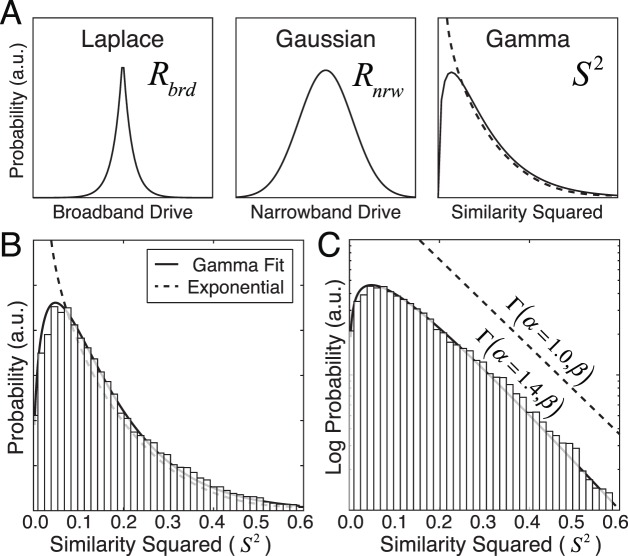
Broadband response drive represented as a Gaussian scale mixture. (A) Laplace-distributed broadband response drives can be expressed as a scale mixture of Gaussian narrowband response drives with a gamma-distributed (i.e., exponential) mixing variable. (B) Squared similarity across all natural stimuli (bars) and a gamma distribution fitted via maximum likelihood (solid curve); an exponential distribution (a gamma distribution with a shape parameter of 1.0) is shown for reference (dashed curve). (C) Same data as (B) on a log-probability axis spanning two orders of magnitude with gamma and exponential fits. The exponential distribution is shifted vertically to reduce clutter. These data are for a vertically oriented cosine-phase Gabor receptive field with an orientation bandwidth of 42°, an octave bandwidth of 1.2, and a preferred frequency of 2 c/°. Results are similar for all receptive fields.

Equation 6B implies that the broadband-normalized responses are distributed as $\newcommand{\bialpha}{\boldsymbol{\alpha}}\newcommand{\bibeta}{\boldsymbol{\beta}}\newcommand{\bigamma}{\boldsymbol{\gamma}}\newcommand{\bidelta}{\boldsymbol{\delta}}\newcommand{\bivarepsilon}{\boldsymbol{\varepsilon}}\newcommand{\bizeta}{\boldsymbol{\zeta}}\newcommand{\bieta}{\boldsymbol{\eta}}\newcommand{\bitheta}{\boldsymbol{\theta}}\newcommand{\biiota}{\boldsymbol{\iota}}\newcommand{\bikappa}{\boldsymbol{\kappa}}\newcommand{\bilambda}{\boldsymbol{\lambda}}\newcommand{\bimu}{\boldsymbol{\mu}}\newcommand{\binu}{\boldsymbol{\nu}}\newcommand{\bixi}{\boldsymbol{\xi}}\newcommand{\biomicron}{\boldsymbol{\micron}}\newcommand{\bipi}{\boldsymbol{\pi}}\newcommand{\birho}{\boldsymbol{\rho}}\newcommand{\bisigma}{\boldsymbol{\sigma}}\newcommand{\bitau}{\boldsymbol{\tau}}\newcommand{\biupsilon}{\boldsymbol{\upsilon}}\newcommand{\biphi}{\boldsymbol{\phi}}\newcommand{\bichi}{\boldsymbol{\chi}}\newcommand{\bipsi}{\boldsymbol{\psi}}\newcommand{\biomega}{\boldsymbol{\omega}}{R_{{{brd}}}}\sim N\left( {0,{S^2}} \right)$ because the narrowband-normalized responses are approximately zero-mean Gaussian (see Figure 4C, 4D). Furthermore, given that the broadband responses are approximately Laplace distributed (see Figure 4C, 4D), Equation 6B also implies that the square of the phase-invariant similarity should be approximately gamma distributed. This is because the Laplace distribution can be represented as a Gaussian scale mixture when the mixing distribution (i.e., the variance of the Gaussian) is gamma distributed with a shape parameter of 1.0—that is, an exponential distribution (Figure 6A) $\newcommand{\bialpha}{\boldsymbol{\alpha}}\newcommand{\bibeta}{\boldsymbol{\beta}}\newcommand{\bigamma}{\boldsymbol{\gamma}}\newcommand{\bidelta}{\boldsymbol{\delta}}\newcommand{\bivarepsilon}{\boldsymbol{\varepsilon}}\newcommand{\bizeta}{\boldsymbol{\zeta}}\newcommand{\bieta}{\boldsymbol{\eta}}\newcommand{\bitheta}{\boldsymbol{\theta}}\newcommand{\biiota}{\boldsymbol{\iota}}\newcommand{\bikappa}{\boldsymbol{\kappa}}\newcommand{\bilambda}{\boldsymbol{\lambda}}\newcommand{\bimu}{\boldsymbol{\mu}}\newcommand{\binu}{\boldsymbol{\nu}}\newcommand{\bixi}{\boldsymbol{\xi}}\newcommand{\biomicron}{\boldsymbol{\micron}}\newcommand{\bipi}{\boldsymbol{\pi}}\newcommand{\birho}{\boldsymbol{\rho}}\newcommand{\bisigma}{\boldsymbol{\sigma}}\newcommand{\bitau}{\boldsymbol{\tau}}\newcommand{\biupsilon}{\boldsymbol{\upsilon}}\newcommand{\biphi}{\boldsymbol{\phi}}\newcommand{\bichi}{\boldsymbol{\chi}}\newcommand{\bipsi}{\boldsymbol{\psi}}\newcommand{\biomega}{\boldsymbol{\omega}}{S^2}\sim \Gamma \left( {\alpha = 1,\beta } \right) = {\rm{Exp}}\left( \beta \right)$ (Ding & Blitzstein, 2018). Figure 6B and 6C shows that $\newcommand{\bialpha}{\boldsymbol{\alpha}}\newcommand{\bibeta}{\boldsymbol{\beta}}\newcommand{\bigamma}{\boldsymbol{\gamma}}\newcommand{\bidelta}{\boldsymbol{\delta}}\newcommand{\bivarepsilon}{\boldsymbol{\varepsilon}}\newcommand{\bizeta}{\boldsymbol{\zeta}}\newcommand{\bieta}{\boldsymbol{\eta}}\newcommand{\bitheta}{\boldsymbol{\theta}}\newcommand{\biiota}{\boldsymbol{\iota}}\newcommand{\bikappa}{\boldsymbol{\kappa}}\newcommand{\bilambda}{\boldsymbol{\lambda}}\newcommand{\bimu}{\boldsymbol{\mu}}\newcommand{\binu}{\boldsymbol{\nu}}\newcommand{\bixi}{\boldsymbol{\xi}}\newcommand{\biomicron}{\boldsymbol{\micron}}\newcommand{\bipi}{\boldsymbol{\pi}}\newcommand{\birho}{\boldsymbol{\rho}}\newcommand{\bisigma}{\boldsymbol{\sigma}}\newcommand{\bitau}{\boldsymbol{\tau}}\newcommand{\biupsilon}{\boldsymbol{\upsilon}}\newcommand{\biphi}{\boldsymbol{\phi}}\newcommand{\bichi}{\boldsymbol{\chi}}\newcommand{\bipsi}{\boldsymbol{\psi}}\newcommand{\biomega}{\boldsymbol{\omega}}{S^2}$ is indeed nicely approximated by a gamma distribution with a shape parameter of 1.4, which is exponential to close approximation. Thus, normalizing the broadband responses by the similarity yields Gaussian-distributed narrowband responses.

Lyu and Simoncelli (2008) also modeled linear filter responses to natural images as a Gaussian scale mixture. Specifically, they modeled the linear (nonnormalized) filter responses as a Gaussian scale mixture. They estimated the value of the mixing random variable from the joint responses of a large bank of multiscale filters. One potential advantage of the work presented here is that the narrowband normalization factor (i.e., the value of the mixing random variable) can be computed directly from the amplitude spectra of the stimulus and the receptive field (Equation 5). Being able to compute the normalization factor directly from the image may make stimulus- and feature-specific normalization easier to implement for some computational investigations. One potential disadvantage is that the approach may be more difficult to adapt for modeling higher cortical areas, where it is more suitable to model inputs as innervation from other neurons rather than as images.

### Normalization pooling region

The receptive field specifies how inputs are weighted and pooled across space to determine the stimulus drive (i.e., $\newcommand{\bialpha}{\boldsymbol{\alpha}}\newcommand{\bibeta}{\boldsymbol{\beta}}\newcommand{\bigamma}{\boldsymbol{\gamma}}\newcommand{\bidelta}{\boldsymbol{\delta}}\newcommand{\bivarepsilon}{\boldsymbol{\varepsilon}}\newcommand{\bizeta}{\boldsymbol{\zeta}}\newcommand{\bieta}{\boldsymbol{\eta}}\newcommand{\bitheta}{\boldsymbol{\theta}}\newcommand{\biiota}{\boldsymbol{\iota}}\newcommand{\bikappa}{\boldsymbol{\kappa}}\newcommand{\bilambda}{\boldsymbol{\lambda}}\newcommand{\bimu}{\boldsymbol{\mu}}\newcommand{\binu}{\boldsymbol{\nu}}\newcommand{\bixi}{\boldsymbol{\xi}}\newcommand{\biomicron}{\boldsymbol{\micron}}\newcommand{\bipi}{\boldsymbol{\pi}}\newcommand{\birho}{\boldsymbol{\rho}}\newcommand{\bisigma}{\boldsymbol{\sigma}}\newcommand{\bitau}{\boldsymbol{\tau}}\newcommand{\biupsilon}{\boldsymbol{\upsilon}}\newcommand{\biphi}{\boldsymbol{\phi}}\newcommand{\bichi}{\boldsymbol{\chi}}\newcommand{\bipsi}{\boldsymbol{\psi}}\newcommand{\biomega}{\boldsymbol{\omega}}{{\bf{f}}^{T}}{\bf{c}}$) to neural response. Receptive fields are typically modeled by a matrix of positive and negative weights that determine how the value of each stimulus pixel contributes to the response (see Figure 3). Under our model, the visual angle spanned by the receptive-field weight matrix impacts the response-drive statistics because it determines the stimulus region from which the normalization factor is computed (Equations 4 and 5; see also later). If the matrix is larger than the preferred feature, it spans image regions outside the classical receptive field, in the so-called surround. When surround regions contribute to the normalization factor, their influence is known as surround suppression (Cavanaugh, Bair, & Movshon, 2002).

**Figure 7:**
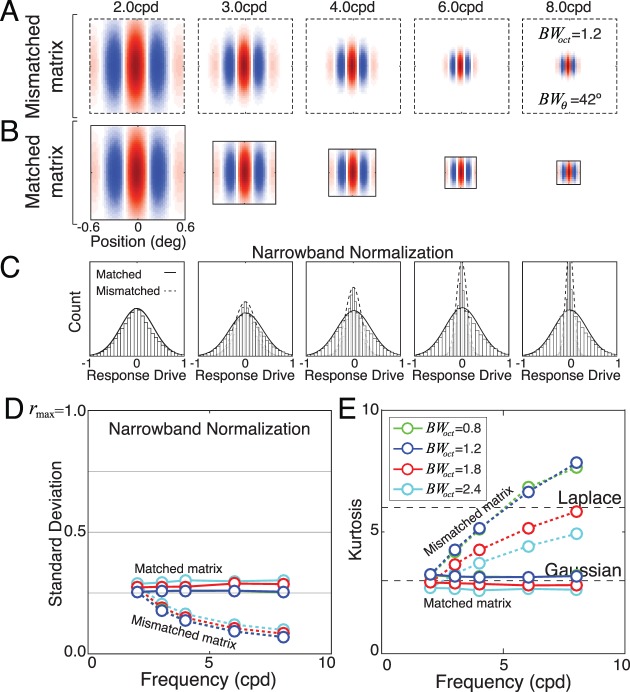
Narrowband response statistics with receptive-field weight matrices that are (A) mismatched and (B) matched to the preferred feature: a vertically oriented Gabor with 1.2-octave bandwidth and 42° orientation bandwidth. (C) Response distributions from matched and mismatched matrices. Matched weight matrices (solid curves) yield response distributions that are invariant to the scale of the preferred feature. Mismatched weight matrices (dashed curves) yield response distributions that change shape and variance with the magnitude of the mismatch. (D) Response standard deviation as a function of preferred spatial frequency for octave bandwidth (colors). Stimulus-driven response variance is constant with preferred frequency when the matrix is matched to the preferred feature. When the matrix is mismatched, response variance decreases with the magnitude of the mismatch. (E) Response kurtosis is the same as a Gaussian with matched weight matrices, but increases with the amount of mismatch.

Consider two sets of neurons employing narrowband contrast normalization having receptive fields with Gabor-shaped preferred features. In the first set, the visual angle spanned by the receptive-field weight matrix becomes increasingly mismatched to its preferred feature with increases in preferred spatial frequency, and includes progressively more stimulus surround relative to the preferred feature (Figure 7A). In the second set, the visual angle spanned by the receptive-field weight matrix is matched to the preferred feature regardless of its spatial frequency (Figure 7B). These two sets of model neurons produce very different sets of response distributions to natural stimuli. When the weight matrix and preferred feature are matched (i.e., span the same visual angle), the normalization factor is computed from the same image region that drives the linear receptive-field response, and the response distributions have constant variance and are approximately Gaussian for all preferred spatial frequencies (Figure 7C, solid curves). When the weight matrix and preferred feature are mismatched, the normalization factor is computed from an image region larger than the preferred feature, the response variance decreases with the inverse frequency (1/*f*) of the preferred feature, and the response drives become less Gaussian (Figure 7C, dashed curves). For the largest mismatch considered here, the narrowband response drives are well approximated by a Laplace distribution. Response drive is strongly suppressed when all the stimulus contrast in a large region surrounding the preferred feature (see Figure 4A) contributes to the normalization factor. Thus, for large mismatches the benefits of narrowband compared to broadband normalization are surrendered (Supplementary Figure S6). On the other hand, when the weight matrix and preferred feature are matched, response drives are Gaussian, zero mean, and invariant to the scale of the preferred feature (Figure 7C through 7E). With matched weight matrices, neural response is thus equally reliable regardless of the preferred spatial frequency. For neural populations preferring a variety of spatial frequencies and scales, this invariance would be computationally convenient for downstream processing.

These results may seem to imply that surround suppression, which occurs in cortex, would prohibit Gaussian response-drive statistics. However, the surround suppression considered thus far is broadband in both orientation and spatial frequency. In cortex, surround suppression is broadband in orientation but passband in spatial frequency (Cavanaugh et al., 2002). With more realistic surround suppression, Gaussian response-drive statistics are preserved (see Discussion).

**Figure 8:**
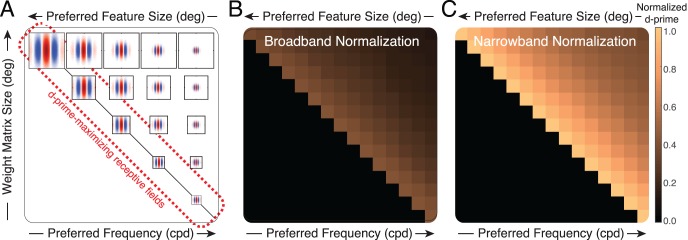
The impact of different receptive-field weight matrices on sensitivity (d′) for stimulus discrimination. (A) Model receptive fields maximize sensitivity for stimulus discrimination when the visual angle subtended by the receptive-field weight matrix matches the visual angle of the preferred feature (on-diagonal receptive fields). When the visual angles are mismatched, sensitivity is lower. (B) Normalized sensitivity with broadband normalization. (C) Normalized sensitivity with narrowband normalization as a function of preferred spatial frequency and the visual angle spanned by the weight matrix.

To summarize the impact of matching the weight matrix to the preferred feature, we plot the sensitivity for stimulus discrimination for a range of preferred features and weight-matrix sizes (Figure 8A). There is a substantial advantage for (a) narrowband over broadband normalization, and (b) matching the visual angles of the receptive-field weight matrix and the preferred stimulus feature (Figure 8B, 8C). Thus, to maximize the sensitivity for stimulus discrimination and to achieve scale-invariant response statistics to natural stimuli, one should perform narrowband normalization with weight matrices that match the receptive field's preferred feature. In other words, the normalization factor should be determined from the same image region that is selected for by the preferred feature.

Matched and mismatched matrices produce different results under the model because of differences in how the normalization factor is computed. To understand why, consider two nearly identical model neurons that prefer the same feature and do *not* employ response normalization (i.e., their response drives equal $\newcommand{\bialpha}{\boldsymbol{\alpha}}\newcommand{\bibeta}{\boldsymbol{\beta}}\newcommand{\bigamma}{\boldsymbol{\gamma}}\newcommand{\bidelta}{\boldsymbol{\delta}}\newcommand{\bivarepsilon}{\boldsymbol{\varepsilon}}\newcommand{\bizeta}{\boldsymbol{\zeta}}\newcommand{\bieta}{\boldsymbol{\eta}}\newcommand{\bitheta}{\boldsymbol{\theta}}\newcommand{\biiota}{\boldsymbol{\iota}}\newcommand{\bikappa}{\boldsymbol{\kappa}}\newcommand{\bilambda}{\boldsymbol{\lambda}}\newcommand{\bimu}{\boldsymbol{\mu}}\newcommand{\binu}{\boldsymbol{\nu}}\newcommand{\bixi}{\boldsymbol{\xi}}\newcommand{\biomicron}{\boldsymbol{\micron}}\newcommand{\bipi}{\boldsymbol{\pi}}\newcommand{\birho}{\boldsymbol{\rho}}\newcommand{\bisigma}{\boldsymbol{\sigma}}\newcommand{\bitau}{\boldsymbol{\tau}}\newcommand{\biupsilon}{\boldsymbol{\upsilon}}\newcommand{\biphi}{\boldsymbol{\phi}}\newcommand{\bichi}{\boldsymbol{\chi}}\newcommand{\bipsi}{\boldsymbol{\psi}}\newcommand{\biomega}{\boldsymbol{\omega}}{{\bf{f}}^{T}}{\bf{c}}$ instead of $\newcommand{\bialpha}{\boldsymbol{\alpha}}\newcommand{\bibeta}{\boldsymbol{\beta}}\newcommand{\bigamma}{\boldsymbol{\gamma}}\newcommand{\bidelta}{\boldsymbol{\delta}}\newcommand{\bivarepsilon}{\boldsymbol{\varepsilon}}\newcommand{\bizeta}{\boldsymbol{\zeta}}\newcommand{\bieta}{\boldsymbol{\eta}}\newcommand{\bitheta}{\boldsymbol{\theta}}\newcommand{\biiota}{\boldsymbol{\iota}}\newcommand{\bikappa}{\boldsymbol{\kappa}}\newcommand{\bilambda}{\boldsymbol{\lambda}}\newcommand{\bimu}{\boldsymbol{\mu}}\newcommand{\binu}{\boldsymbol{\nu}}\newcommand{\bixi}{\boldsymbol{\xi}}\newcommand{\biomicron}{\boldsymbol{\micron}}\newcommand{\bipi}{\boldsymbol{\pi}}\newcommand{\birho}{\boldsymbol{\rho}}\newcommand{\bisigma}{\boldsymbol{\sigma}}\newcommand{\bitau}{\boldsymbol{\tau}}\newcommand{\biupsilon}{\boldsymbol{\upsilon}}\newcommand{\biphi}{\boldsymbol{\phi}}\newcommand{\bichi}{\boldsymbol{\chi}}\newcommand{\bipsi}{\boldsymbol{\psi}}\newcommand{\biomega}{\boldsymbol{\omega}}{{{{\bf{f}}^{T}}{\bf{c}}} \mathord{\left/ {\vphantom {{{{\bf{f}}^{T}}{\bf{c}}} N}} \right. \kern-1.2pt} N}$). The neurons differ only because one has a matched and the other has a mismatched weight matrix; the mismatched matrix is identical to the matched matrix except that it is padded with zero-valued coefficients. Multiplying inputs with zero-valued coefficients does not change the linear response. Thus, both neurons will yield identical linear responses. The fact that model neurons employing response normalization yield different results with matched and mismatched matrices must therefore be due to the size of the image region determining the normalization factor, relative to the size of the preferred feature of the receptive field.

The visual angle spanned by the weight matrix determines the image region from which the normalization factor is computed, under the model. With mismatched matrices, the normalization factor is determined from the stimulus contrast in an image region larger than the preferred feature, which will likely contain spatial frequencies lower than the preferred frequency. Natural images have 1/*f* amplitude spectra (D. J. Field, 1987); contrast energy at frequencies lower than the preferred frequency is likely to dominate and substantially increase the value of the normalization factor, thereby decreasing the normalized response. As the mismatch increases, the decrease in the normalized response becomes more pronounced, reducing the stimulus-driven response variability associated with high-frequency features.

### Downsampling

**Figure 9:**
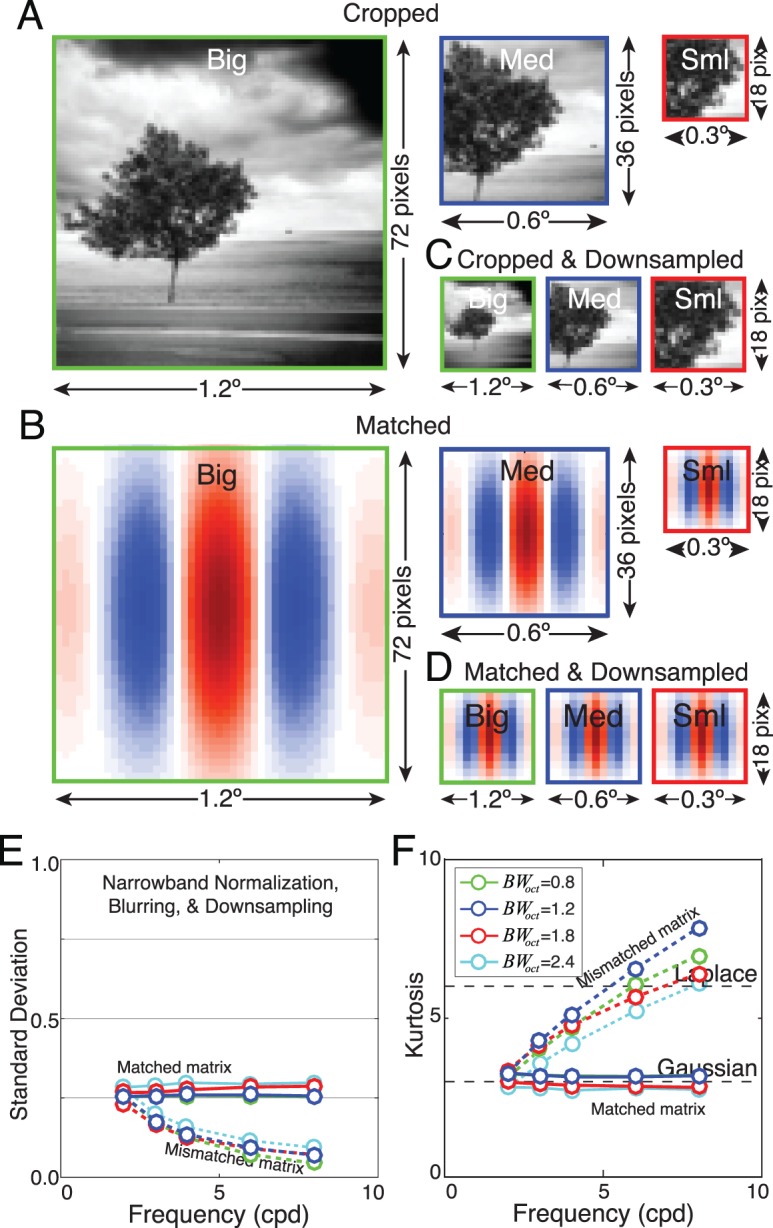
Downsampling cropped images for matched receptive-field weight matrices. (A) Cropped images: big, medium, and small. Boxes indicate three different scales at which image data are processed. The cropped images span different visual angles but have a fixed sampling rate. Each cropped image therefore has a different number of pixels. (B) Matched receptive-field weight matrices. The visual angle spanned by the weight matrix matches the visual angle spanned by the preferred feature. Each matrix also has a different number of pixels. (C) Cropped and downsampled images. Cropped and downsampled images span different visual angles, but have the same number of pixels. (D) Matched and downsampled receptive fields. All weight matrices have the same number of pixels, regardless of the spanned visual angle. (E–F) The impact of downsampling on response statistics: (E) Standard deviation of stimulus-driven response with narrowband normalization, blurring, and downsampling. (F) Kurtosis of stimulus-driven response with blurring and downsampling. The response statistics are essentially identical with and without downsampling.

The sampling resolution within the pooling region is another property of the response-normalization model that may impact response-drive statistics. The model-neuron receptive fields that we have considered thus far have had identical sampling resolution, so the number of pixels representing a preferred feature scales with the visual angle spanned by the receptive field (Figure 9A, 9B). In the primate visual system, at a given eccentricity, simple cells with larger receptive fields pool over more relay-cell inputs from the lateral geniculate nucleus than those with smaller receptive fields (Taylor, Sedigh-Sarvestani, Vigeland, Palmer, & Contreras, 2018). Similarly, parasol ganglion cells in the retina pool over more cone receptors than midget ganglion cells (G. D. Field et al., 2010). In other stages of processing, the inputs pooled by large receptive fields have lower sampling resolution than their smaller counterparts. For example, large retinal ganglion cells in the retinal periphery pool inputs from large low-resolution cone photoreceptors, whereas small near-foveal retinal ganglion cells of the same type pool over small high-resolution photoreceptors (Croner & Kaplan, 1995; Rossi & Roorda, 2010). The processing motif employed by the peripheral retina is roughly equivalent to downsampling, a common preprocessing method in the computer-vision, image-processing, and deep-learning communities (Burt & Adelson, 1983). In general, downsampling reduces the number of pixels (i.e., the sampling resolution) representing a particular image patch, and hence the computational requirements for processing that patch.

We asked how downsampling the input stimuli impacts model-neuron response-drive statistics. First we generated a new set of receptive-field weight matrices where the sampling resolution was yoked to the visual angle spanned by the preferred feature. The result was a set of receptive fields defined by weight matrices that all had an identical number of pixels. Specifically, all weight matrices had 18 × 18 pixels, the same number as the corresponding original matrix for the smallest preferred feature (0.3°, 8 c/°). Then we downsampled the image patches (after blurring to prevent aliasing) to match the sampling resolution of the receptive fields (Figure 9C, 9D; see Methods). Receptive fields with matched downsampled weight matrices yield constant-variance, Gaussian-distributed response drives (Figure 9E, 9F). The response statistics (i.e., standard deviation and kurtosis) are within 1% of those without downsampling (see Figure 7D, 7E). There is no disadvantage (or advantage) to downsampling in terms of the sensitivity for signal discrimination. Thus, at least in terms of sensitivity for stimulus discrimination, there is no pressure on the visual system to avoid downsampling. This result may be useful for approaches that seek to learn receptive fields via nonparametric methods, where every additional pixel in a receptive-field weight matrix incurs considerable computational cost (see Discussion).

## Methods

### Natural stimuli

Natural-image patches were sampled from two recently published photographic databases of natural scenes (Burge & Geisler, 2011; Burge, McCann, & Geisler, 2016). Scenes were photographed on and around the campus of the University of Texas at Austin and contained grass, shrubs, trees, streets, cars, and buildings. The images were calibrated such that the intensity values were linear with luminance. The data represent 30,888 unique 1.2° image patches (72 × 72 pixels); 312 nonoverlapping patches were randomly selected from each of 99 calibrated natural images. For receptive fields that were rectangular rather than square, the sizes of the image patches were adjusted accordingly.

### Local contrast

The intensity patches were converted to Weber-contrast images by luminance normalization. The contrast image was obtained by subtracting off and dividing by the local mean intensity

\newcommand{\bialpha}{\boldsymbol{\alpha}}\newcommand{\bibeta}{\boldsymbol{\beta}}\newcommand{\bigamma}{\boldsymbol{\gamma}}\newcommand{\bidelta}{\boldsymbol{\delta}}\newcommand{\bivarepsilon}{\boldsymbol{\varepsilon}}\newcommand{\bizeta}{\boldsymbol{\zeta}}\newcommand{\bieta}{\boldsymbol{\eta}}\newcommand{\bitheta}{\boldsymbol{\theta}}\newcommand{\biiota}{\boldsymbol{\iota}}\newcommand{\bikappa}{\boldsymbol{\kappa}}\newcommand{\bilambda}{\boldsymbol{\lambda}}\newcommand{\bimu}{\boldsymbol{\mu}}\newcommand{\binu}{\boldsymbol{\nu}}\newcommand{\bixi}{\boldsymbol{\xi}}\newcommand{\biomicron}{\boldsymbol{\micron}}\newcommand{\bipi}{\boldsymbol{\pi}}\newcommand{\birho}{\boldsymbol{\rho}}\newcommand{\bisigma}{\boldsymbol{\sigma}}\newcommand{\bitau}{\boldsymbol{\tau}}\newcommand{\biupsilon}{\boldsymbol{\upsilon}}\newcommand{\biphi}{\boldsymbol{\phi}}\newcommand{\bichi}{\boldsymbol{\chi}}\newcommand{\bipsi}{\boldsymbol{\psi}}\newcommand{\biomega}{\boldsymbol{\omega}}\begin{equation}\tag{7}{\bf{c}}\left( {\bf{x}} \right) = \sum\limits_{{\bf{x}} \in A} {\left[ {\left( {{{I\left( {\bf{x}} \right) - \bar I} \over {\bar I}}} \right)} \right]} {\rm ,}\end{equation}

where $\newcommand{\bialpha}{\boldsymbol{\alpha}}\newcommand{\bibeta}{\boldsymbol{\beta}}\newcommand{\bigamma}{\boldsymbol{\gamma}}\newcommand{\bidelta}{\boldsymbol{\delta}}\newcommand{\bivarepsilon}{\boldsymbol{\varepsilon}}\newcommand{\bizeta}{\boldsymbol{\zeta}}\newcommand{\bieta}{\boldsymbol{\eta}}\newcommand{\bitheta}{\boldsymbol{\theta}}\newcommand{\biiota}{\boldsymbol{\iota}}\newcommand{\bikappa}{\boldsymbol{\kappa}}\newcommand{\bilambda}{\boldsymbol{\lambda}}\newcommand{\bimu}{\boldsymbol{\mu}}\newcommand{\binu}{\boldsymbol{\nu}}\newcommand{\bixi}{\boldsymbol{\xi}}\newcommand{\biomicron}{\boldsymbol{\micron}}\newcommand{\bipi}{\boldsymbol{\pi}}\newcommand{\birho}{\boldsymbol{\rho}}\newcommand{\bisigma}{\boldsymbol{\sigma}}\newcommand{\bitau}{\boldsymbol{\tau}}\newcommand{\biupsilon}{\boldsymbol{\upsilon}}\newcommand{\biphi}{\boldsymbol{\phi}}\newcommand{\bichi}{\boldsymbol{\chi}}\newcommand{\bipsi}{\boldsymbol{\psi}}\newcommand{\biomega}{\boldsymbol{\omega}}{\bf{c}}\left( {\bf{x}} \right)$ is the local-contrast image patch, $\newcommand{\bialpha}{\boldsymbol{\alpha}}\newcommand{\bibeta}{\boldsymbol{\beta}}\newcommand{\bigamma}{\boldsymbol{\gamma}}\newcommand{\bidelta}{\boldsymbol{\delta}}\newcommand{\bivarepsilon}{\boldsymbol{\varepsilon}}\newcommand{\bizeta}{\boldsymbol{\zeta}}\newcommand{\bieta}{\boldsymbol{\eta}}\newcommand{\bitheta}{\boldsymbol{\theta}}\newcommand{\biiota}{\boldsymbol{\iota}}\newcommand{\bikappa}{\boldsymbol{\kappa}}\newcommand{\bilambda}{\boldsymbol{\lambda}}\newcommand{\bimu}{\boldsymbol{\mu}}\newcommand{\binu}{\boldsymbol{\nu}}\newcommand{\bixi}{\boldsymbol{\xi}}\newcommand{\biomicron}{\boldsymbol{\micron}}\newcommand{\bipi}{\boldsymbol{\pi}}\newcommand{\birho}{\boldsymbol{\rho}}\newcommand{\bisigma}{\boldsymbol{\sigma}}\newcommand{\bitau}{\boldsymbol{\tau}}\newcommand{\biupsilon}{\boldsymbol{\upsilon}}\newcommand{\biphi}{\boldsymbol{\phi}}\newcommand{\bichi}{\boldsymbol{\chi}}\newcommand{\bipsi}{\boldsymbol{\psi}}\newcommand{\biomega}{\boldsymbol{\omega}}I\left( {\bf{x}} \right)$ is the local-intensity image patch, $\newcommand{\bialpha}{\boldsymbol{\alpha}}\newcommand{\bibeta}{\boldsymbol{\beta}}\newcommand{\bigamma}{\boldsymbol{\gamma}}\newcommand{\bidelta}{\boldsymbol{\delta}}\newcommand{\bivarepsilon}{\boldsymbol{\varepsilon}}\newcommand{\bizeta}{\boldsymbol{\zeta}}\newcommand{\bieta}{\boldsymbol{\eta}}\newcommand{\bitheta}{\boldsymbol{\theta}}\newcommand{\biiota}{\boldsymbol{\iota}}\newcommand{\bikappa}{\boldsymbol{\kappa}}\newcommand{\bilambda}{\boldsymbol{\lambda}}\newcommand{\bimu}{\boldsymbol{\mu}}\newcommand{\binu}{\boldsymbol{\nu}}\newcommand{\bixi}{\boldsymbol{\xi}}\newcommand{\biomicron}{\boldsymbol{\micron}}\newcommand{\bipi}{\boldsymbol{\pi}}\newcommand{\birho}{\boldsymbol{\rho}}\newcommand{\bisigma}{\boldsymbol{\sigma}}\newcommand{\bitau}{\boldsymbol{\tau}}\newcommand{\biupsilon}{\boldsymbol{\upsilon}}\newcommand{\biphi}{\boldsymbol{\phi}}\newcommand{\bichi}{\boldsymbol{\chi}}\newcommand{\bipsi}{\boldsymbol{\psi}}\newcommand{\biomega}{\boldsymbol{\omega}}\bar I$ is the local mean intensity, and $\newcommand{\bialpha}{\boldsymbol{\alpha}}\newcommand{\bibeta}{\boldsymbol{\beta}}\newcommand{\bigamma}{\boldsymbol{\gamma}}\newcommand{\bidelta}{\boldsymbol{\delta}}\newcommand{\bivarepsilon}{\boldsymbol{\varepsilon}}\newcommand{\bizeta}{\boldsymbol{\zeta}}\newcommand{\bieta}{\boldsymbol{\eta}}\newcommand{\bitheta}{\boldsymbol{\theta}}\newcommand{\biiota}{\boldsymbol{\iota}}\newcommand{\bikappa}{\boldsymbol{\kappa}}\newcommand{\bilambda}{\boldsymbol{\lambda}}\newcommand{\bimu}{\boldsymbol{\mu}}\newcommand{\binu}{\boldsymbol{\nu}}\newcommand{\bixi}{\boldsymbol{\xi}}\newcommand{\biomicron}{\boldsymbol{\micron}}\newcommand{\bipi}{\boldsymbol{\pi}}\newcommand{\birho}{\boldsymbol{\rho}}\newcommand{\bisigma}{\boldsymbol{\sigma}}\newcommand{\bitau}{\boldsymbol{\tau}}\newcommand{\biupsilon}{\boldsymbol{\upsilon}}\newcommand{\biphi}{\boldsymbol{\phi}}\newcommand{\bichi}{\boldsymbol{\chi}}\newcommand{\bipsi}{\boldsymbol{\psi}}\newcommand{\biomega}{\boldsymbol{\omega}}{\bf{x}} = \left\{ {x,y} \right\}$ indexes spatial position in the area $\newcommand{\bialpha}{\boldsymbol{\alpha}}\newcommand{\bibeta}{\boldsymbol{\beta}}\newcommand{\bigamma}{\boldsymbol{\gamma}}\newcommand{\bidelta}{\boldsymbol{\delta}}\newcommand{\bivarepsilon}{\boldsymbol{\varepsilon}}\newcommand{\bizeta}{\boldsymbol{\zeta}}\newcommand{\bieta}{\boldsymbol{\eta}}\newcommand{\bitheta}{\boldsymbol{\theta}}\newcommand{\biiota}{\boldsymbol{\iota}}\newcommand{\bikappa}{\boldsymbol{\kappa}}\newcommand{\bilambda}{\boldsymbol{\lambda}}\newcommand{\bimu}{\boldsymbol{\mu}}\newcommand{\binu}{\boldsymbol{\nu}}\newcommand{\bixi}{\boldsymbol{\xi}}\newcommand{\biomicron}{\boldsymbol{\micron}}\newcommand{\bipi}{\boldsymbol{\pi}}\newcommand{\birho}{\boldsymbol{\rho}}\newcommand{\bisigma}{\boldsymbol{\sigma}}\newcommand{\bitau}{\boldsymbol{\tau}}\newcommand{\biupsilon}{\boldsymbol{\upsilon}}\newcommand{\biphi}{\boldsymbol{\phi}}\newcommand{\bichi}{\boldsymbol{\chi}}\newcommand{\bipsi}{\boldsymbol{\psi}}\newcommand{\biomega}{\boldsymbol{\omega}}A$ spanned by the receptive-field weight matrix. The local mean intensity is given by $\newcommand{\bialpha}{\boldsymbol{\alpha}}\newcommand{\bibeta}{\boldsymbol{\beta}}\newcommand{\bigamma}{\boldsymbol{\gamma}}\newcommand{\bidelta}{\boldsymbol{\delta}}\newcommand{\bivarepsilon}{\boldsymbol{\varepsilon}}\newcommand{\bizeta}{\boldsymbol{\zeta}}\newcommand{\bieta}{\boldsymbol{\eta}}\newcommand{\bitheta}{\boldsymbol{\theta}}\newcommand{\biiota}{\boldsymbol{\iota}}\newcommand{\bikappa}{\boldsymbol{\kappa}}\newcommand{\bilambda}{\boldsymbol{\lambda}}\newcommand{\bimu}{\boldsymbol{\mu}}\newcommand{\binu}{\boldsymbol{\nu}}\newcommand{\bixi}{\boldsymbol{\xi}}\newcommand{\biomicron}{\boldsymbol{\micron}}\newcommand{\bipi}{\boldsymbol{\pi}}\newcommand{\birho}{\boldsymbol{\rho}}\newcommand{\bisigma}{\boldsymbol{\sigma}}\newcommand{\bitau}{\boldsymbol{\tau}}\newcommand{\biupsilon}{\boldsymbol{\upsilon}}\newcommand{\biphi}{\boldsymbol{\phi}}\newcommand{\bichi}{\boldsymbol{\chi}}\newcommand{\bipsi}{\boldsymbol{\psi}}\newcommand{\biomega}{\boldsymbol{\omega}}\bar I = \sum\limits_{{\bf{x}} \in A} {\left[ {I\left( {\bf{x}} \right)} \right]}$.

### Receptive fields

The receptive field of each model neuron was modeled with a weight matrix. The receptive-field weight matrix was determined by the preferred feature, the visual angle spanned by the weight matrix, and the spatial sampling rate. The preferred feature of each model neuron was modeled as a Gabor. A Gabor is a cosine wave multiplied by a Gaussian envelope:

\newcommand{\bialpha}{\boldsymbol{\alpha}}\newcommand{\bibeta}{\boldsymbol{\beta}}\newcommand{\bigamma}{\boldsymbol{\gamma}}\newcommand{\bidelta}{\boldsymbol{\delta}}\newcommand{\bivarepsilon}{\boldsymbol{\varepsilon}}\newcommand{\bizeta}{\boldsymbol{\zeta}}\newcommand{\bieta}{\boldsymbol{\eta}}\newcommand{\bitheta}{\boldsymbol{\theta}}\newcommand{\biiota}{\boldsymbol{\iota}}\newcommand{\bikappa}{\boldsymbol{\kappa}}\newcommand{\bilambda}{\boldsymbol{\lambda}}\newcommand{\bimu}{\boldsymbol{\mu}}\newcommand{\binu}{\boldsymbol{\nu}}\newcommand{\bixi}{\boldsymbol{\xi}}\newcommand{\biomicron}{\boldsymbol{\micron}}\newcommand{\bipi}{\boldsymbol{\pi}}\newcommand{\birho}{\boldsymbol{\rho}}\newcommand{\bisigma}{\boldsymbol{\sigma}}\newcommand{\bitau}{\boldsymbol{\tau}}\newcommand{\biupsilon}{\boldsymbol{\upsilon}}\newcommand{\biphi}{\boldsymbol{\phi}}\newcommand{\bichi}{\boldsymbol{\chi}}\newcommand{\bipsi}{\boldsymbol{\psi}}\newcommand{\biomega}{\boldsymbol{\omega}}\begin{equation}\tag{8}{\bf{f}}\left( {\bf{x}} \right) = {\rm{gauss}}\left( {x^{\prime} ,y^{\prime} ;{x_0},{y_0},{\theta _{0,}}{\sigma _b},{\sigma _l}} \right)\cos \left( {2\pi {f_0}x^{\prime} + {\phi _0}} \right){\rm ,}\end{equation}

where $\newcommand{\bialpha}{\boldsymbol{\alpha}}\newcommand{\bibeta}{\boldsymbol{\beta}}\newcommand{\bigamma}{\boldsymbol{\gamma}}\newcommand{\bidelta}{\boldsymbol{\delta}}\newcommand{\bivarepsilon}{\boldsymbol{\varepsilon}}\newcommand{\bizeta}{\boldsymbol{\zeta}}\newcommand{\bieta}{\boldsymbol{\eta}}\newcommand{\bitheta}{\boldsymbol{\theta}}\newcommand{\biiota}{\boldsymbol{\iota}}\newcommand{\bikappa}{\boldsymbol{\kappa}}\newcommand{\bilambda}{\boldsymbol{\lambda}}\newcommand{\bimu}{\boldsymbol{\mu}}\newcommand{\binu}{\boldsymbol{\nu}}\newcommand{\bixi}{\boldsymbol{\xi}}\newcommand{\biomicron}{\boldsymbol{\micron}}\newcommand{\bipi}{\boldsymbol{\pi}}\newcommand{\birho}{\boldsymbol{\rho}}\newcommand{\bisigma}{\boldsymbol{\sigma}}\newcommand{\bitau}{\boldsymbol{\tau}}\newcommand{\biupsilon}{\boldsymbol{\upsilon}}\newcommand{\biphi}{\boldsymbol{\phi}}\newcommand{\bichi}{\boldsymbol{\chi}}\newcommand{\bipsi}{\boldsymbol{\psi}}\newcommand{\biomega}{\boldsymbol{\omega}}{x_0}$ and $\newcommand{\bialpha}{\boldsymbol{\alpha}}\newcommand{\bibeta}{\boldsymbol{\beta}}\newcommand{\bigamma}{\boldsymbol{\gamma}}\newcommand{\bidelta}{\boldsymbol{\delta}}\newcommand{\bivarepsilon}{\boldsymbol{\varepsilon}}\newcommand{\bizeta}{\boldsymbol{\zeta}}\newcommand{\bieta}{\boldsymbol{\eta}}\newcommand{\bitheta}{\boldsymbol{\theta}}\newcommand{\biiota}{\boldsymbol{\iota}}\newcommand{\bikappa}{\boldsymbol{\kappa}}\newcommand{\bilambda}{\boldsymbol{\lambda}}\newcommand{\bimu}{\boldsymbol{\mu}}\newcommand{\binu}{\boldsymbol{\nu}}\newcommand{\bixi}{\boldsymbol{\xi}}\newcommand{\biomicron}{\boldsymbol{\micron}}\newcommand{\bipi}{\boldsymbol{\pi}}\newcommand{\birho}{\boldsymbol{\rho}}\newcommand{\bisigma}{\boldsymbol{\sigma}}\newcommand{\bitau}{\boldsymbol{\tau}}\newcommand{\biupsilon}{\boldsymbol{\upsilon}}\newcommand{\biphi}{\boldsymbol{\phi}}\newcommand{\bichi}{\boldsymbol{\chi}}\newcommand{\bipsi}{\boldsymbol{\psi}}\newcommand{\biomega}{\boldsymbol{\omega}}{y_0}$ specify the position of the Gaussian envelope, $\newcommand{\bialpha}{\boldsymbol{\alpha}}\newcommand{\bibeta}{\boldsymbol{\beta}}\newcommand{\bigamma}{\boldsymbol{\gamma}}\newcommand{\bidelta}{\boldsymbol{\delta}}\newcommand{\bivarepsilon}{\boldsymbol{\varepsilon}}\newcommand{\bizeta}{\boldsymbol{\zeta}}\newcommand{\bieta}{\boldsymbol{\eta}}\newcommand{\bitheta}{\boldsymbol{\theta}}\newcommand{\biiota}{\boldsymbol{\iota}}\newcommand{\bikappa}{\boldsymbol{\kappa}}\newcommand{\bilambda}{\boldsymbol{\lambda}}\newcommand{\bimu}{\boldsymbol{\mu}}\newcommand{\binu}{\boldsymbol{\nu}}\newcommand{\bixi}{\boldsymbol{\xi}}\newcommand{\biomicron}{\boldsymbol{\micron}}\newcommand{\bipi}{\boldsymbol{\pi}}\newcommand{\birho}{\boldsymbol{\rho}}\newcommand{\bisigma}{\boldsymbol{\sigma}}\newcommand{\bitau}{\boldsymbol{\tau}}\newcommand{\biupsilon}{\boldsymbol{\upsilon}}\newcommand{\biphi}{\boldsymbol{\phi}}\newcommand{\bichi}{\boldsymbol{\chi}}\newcommand{\bipsi}{\boldsymbol{\psi}}\newcommand{\biomega}{\boldsymbol{\omega}}{\theta _0}$ is the preferred orientation, $\newcommand{\bialpha}{\boldsymbol{\alpha}}\newcommand{\bibeta}{\boldsymbol{\beta}}\newcommand{\bigamma}{\boldsymbol{\gamma}}\newcommand{\bidelta}{\boldsymbol{\delta}}\newcommand{\bivarepsilon}{\boldsymbol{\varepsilon}}\newcommand{\bizeta}{\boldsymbol{\zeta}}\newcommand{\bieta}{\boldsymbol{\eta}}\newcommand{\bitheta}{\boldsymbol{\theta}}\newcommand{\biiota}{\boldsymbol{\iota}}\newcommand{\bikappa}{\boldsymbol{\kappa}}\newcommand{\bilambda}{\boldsymbol{\lambda}}\newcommand{\bimu}{\boldsymbol{\mu}}\newcommand{\binu}{\boldsymbol{\nu}}\newcommand{\bixi}{\boldsymbol{\xi}}\newcommand{\biomicron}{\boldsymbol{\micron}}\newcommand{\bipi}{\boldsymbol{\pi}}\newcommand{\birho}{\boldsymbol{\rho}}\newcommand{\bisigma}{\boldsymbol{\sigma}}\newcommand{\bitau}{\boldsymbol{\tau}}\newcommand{\biupsilon}{\boldsymbol{\upsilon}}\newcommand{\biphi}{\boldsymbol{\phi}}\newcommand{\bichi}{\boldsymbol{\chi}}\newcommand{\bipsi}{\boldsymbol{\psi}}\newcommand{\biomega}{\boldsymbol{\omega}}{\sigma _b}$ is the standard deviation of the envelope in the band-pass direction (orthogonal to the grating orientation), $\newcommand{\bialpha}{\boldsymbol{\alpha}}\newcommand{\bibeta}{\boldsymbol{\beta}}\newcommand{\bigamma}{\boldsymbol{\gamma}}\newcommand{\bidelta}{\boldsymbol{\delta}}\newcommand{\bivarepsilon}{\boldsymbol{\varepsilon}}\newcommand{\bizeta}{\boldsymbol{\zeta}}\newcommand{\bieta}{\boldsymbol{\eta}}\newcommand{\bitheta}{\boldsymbol{\theta}}\newcommand{\biiota}{\boldsymbol{\iota}}\newcommand{\bikappa}{\boldsymbol{\kappa}}\newcommand{\bilambda}{\boldsymbol{\lambda}}\newcommand{\bimu}{\boldsymbol{\mu}}\newcommand{\binu}{\boldsymbol{\nu}}\newcommand{\bixi}{\boldsymbol{\xi}}\newcommand{\biomicron}{\boldsymbol{\micron}}\newcommand{\bipi}{\boldsymbol{\pi}}\newcommand{\birho}{\boldsymbol{\rho}}\newcommand{\bisigma}{\boldsymbol{\sigma}}\newcommand{\bitau}{\boldsymbol{\tau}}\newcommand{\biupsilon}{\boldsymbol{\upsilon}}\newcommand{\biphi}{\boldsymbol{\phi}}\newcommand{\bichi}{\boldsymbol{\chi}}\newcommand{\bipsi}{\boldsymbol{\psi}}\newcommand{\biomega}{\boldsymbol{\omega}}{\sigma _l}$ is the standard deviation of the envelope in the low-pass direction (parallel to the grating orientation), $\newcommand{\bialpha}{\boldsymbol{\alpha}}\newcommand{\bibeta}{\boldsymbol{\beta}}\newcommand{\bigamma}{\boldsymbol{\gamma}}\newcommand{\bidelta}{\boldsymbol{\delta}}\newcommand{\bivarepsilon}{\boldsymbol{\varepsilon}}\newcommand{\bizeta}{\boldsymbol{\zeta}}\newcommand{\bieta}{\boldsymbol{\eta}}\newcommand{\bitheta}{\boldsymbol{\theta}}\newcommand{\biiota}{\boldsymbol{\iota}}\newcommand{\bikappa}{\boldsymbol{\kappa}}\newcommand{\bilambda}{\boldsymbol{\lambda}}\newcommand{\bimu}{\boldsymbol{\mu}}\newcommand{\binu}{\boldsymbol{\nu}}\newcommand{\bixi}{\boldsymbol{\xi}}\newcommand{\biomicron}{\boldsymbol{\micron}}\newcommand{\bipi}{\boldsymbol{\pi}}\newcommand{\birho}{\boldsymbol{\rho}}\newcommand{\bisigma}{\boldsymbol{\sigma}}\newcommand{\bitau}{\boldsymbol{\tau}}\newcommand{\biupsilon}{\boldsymbol{\upsilon}}\newcommand{\biphi}{\boldsymbol{\phi}}\newcommand{\bichi}{\boldsymbol{\chi}}\newcommand{\bipsi}{\boldsymbol{\psi}}\newcommand{\biomega}{\boldsymbol{\omega}}{f_0}$ is the preferred spatial frequency, $\newcommand{\bialpha}{\boldsymbol{\alpha}}\newcommand{\bibeta}{\boldsymbol{\beta}}\newcommand{\bigamma}{\boldsymbol{\gamma}}\newcommand{\bidelta}{\boldsymbol{\delta}}\newcommand{\bivarepsilon}{\boldsymbol{\varepsilon}}\newcommand{\bizeta}{\boldsymbol{\zeta}}\newcommand{\bieta}{\boldsymbol{\eta}}\newcommand{\bitheta}{\boldsymbol{\theta}}\newcommand{\biiota}{\boldsymbol{\iota}}\newcommand{\bikappa}{\boldsymbol{\kappa}}\newcommand{\bilambda}{\boldsymbol{\lambda}}\newcommand{\bimu}{\boldsymbol{\mu}}\newcommand{\binu}{\boldsymbol{\nu}}\newcommand{\bixi}{\boldsymbol{\xi}}\newcommand{\biomicron}{\boldsymbol{\micron}}\newcommand{\bipi}{\boldsymbol{\pi}}\newcommand{\birho}{\boldsymbol{\rho}}\newcommand{\bisigma}{\boldsymbol{\sigma}}\newcommand{\bitau}{\boldsymbol{\tau}}\newcommand{\biupsilon}{\boldsymbol{\upsilon}}\newcommand{\biphi}{\boldsymbol{\phi}}\newcommand{\bichi}{\boldsymbol{\chi}}\newcommand{\bipsi}{\boldsymbol{\psi}}\newcommand{\biomega}{\boldsymbol{\omega}}{\phi _0}$ is the preferred phase, and $\newcommand{\bialpha}{\boldsymbol{\alpha}}\newcommand{\bibeta}{\boldsymbol{\beta}}\newcommand{\bigamma}{\boldsymbol{\gamma}}\newcommand{\bidelta}{\boldsymbol{\delta}}\newcommand{\bivarepsilon}{\boldsymbol{\varepsilon}}\newcommand{\bizeta}{\boldsymbol{\zeta}}\newcommand{\bieta}{\boldsymbol{\eta}}\newcommand{\bitheta}{\boldsymbol{\theta}}\newcommand{\biiota}{\boldsymbol{\iota}}\newcommand{\bikappa}{\boldsymbol{\kappa}}\newcommand{\bilambda}{\boldsymbol{\lambda}}\newcommand{\bimu}{\boldsymbol{\mu}}\newcommand{\binu}{\boldsymbol{\nu}}\newcommand{\bixi}{\boldsymbol{\xi}}\newcommand{\biomicron}{\boldsymbol{\micron}}\newcommand{\bipi}{\boldsymbol{\pi}}\newcommand{\birho}{\boldsymbol{\rho}}\newcommand{\bisigma}{\boldsymbol{\sigma}}\newcommand{\bitau}{\boldsymbol{\tau}}\newcommand{\biupsilon}{\boldsymbol{\upsilon}}\newcommand{\biphi}{\boldsymbol{\phi}}\newcommand{\bichi}{\boldsymbol{\chi}}\newcommand{\bipsi}{\boldsymbol{\psi}}\newcommand{\biomega}{\boldsymbol{\omega}}\left\{ {x^{\prime} ,y^{\prime} } \right\}$ are transformed coordinates due to the preferred orientation

\newcommand{\bialpha}{\boldsymbol{\alpha}}\newcommand{\bibeta}{\boldsymbol{\beta}}\newcommand{\bigamma}{\boldsymbol{\gamma}}\newcommand{\bidelta}{\boldsymbol{\delta}}\newcommand{\bivarepsilon}{\boldsymbol{\varepsilon}}\newcommand{\bizeta}{\boldsymbol{\zeta}}\newcommand{\bieta}{\boldsymbol{\eta}}\newcommand{\bitheta}{\boldsymbol{\theta}}\newcommand{\biiota}{\boldsymbol{\iota}}\newcommand{\bikappa}{\boldsymbol{\kappa}}\newcommand{\bilambda}{\boldsymbol{\lambda}}\newcommand{\bimu}{\boldsymbol{\mu}}\newcommand{\binu}{\boldsymbol{\nu}}\newcommand{\bixi}{\boldsymbol{\xi}}\newcommand{\biomicron}{\boldsymbol{\micron}}\newcommand{\bipi}{\boldsymbol{\pi}}\newcommand{\birho}{\boldsymbol{\rho}}\newcommand{\bisigma}{\boldsymbol{\sigma}}\newcommand{\bitau}{\boldsymbol{\tau}}\newcommand{\biupsilon}{\boldsymbol{\upsilon}}\newcommand{\biphi}{\boldsymbol{\phi}}\newcommand{\bichi}{\boldsymbol{\chi}}\newcommand{\bipsi}{\boldsymbol{\psi}}\newcommand{\biomega}{\boldsymbol{\omega}}\begin{equation}\tag{9}\left[ {\matrix{ {x^{\prime} } \cr {y^{\prime} } \cr } } \right] = \left[ {\left( {\matrix{ {{\rm{\ \ }}\cos {\theta _0}}&{\sin {\theta _0}} \cr { - \sin {\theta _0}}&{\cos {\theta _0}} \cr } } \right)} \right]\left[ {\matrix{ {x - {x_0}} \cr {y - {y_0}} \cr } } \right] = \left[ {\matrix{ {{\rm{\ \ }}\left( {x - {x_0}} \right)\cos {\theta _0} + \left( {y - {y_0}} \right)\sin {\theta _0}} \cr { - \left( {x - {x_0}} \right)\sin {\theta _0} + \left( {y - {y_0}} \right)\cos {\theta _0}} \cr } } \right]{\rm .}\end{equation}

The coefficients of the receptive-field weight matrix are normalized such that the L2 norm of the receptive-field weight coefficients $\newcommand{\bialpha}{\boldsymbol{\alpha}}\newcommand{\bibeta}{\boldsymbol{\beta}}\newcommand{\bigamma}{\boldsymbol{\gamma}}\newcommand{\bidelta}{\boldsymbol{\delta}}\newcommand{\bivarepsilon}{\boldsymbol{\varepsilon}}\newcommand{\bizeta}{\boldsymbol{\zeta}}\newcommand{\bieta}{\boldsymbol{\eta}}\newcommand{\bitheta}{\boldsymbol{\theta}}\newcommand{\biiota}{\boldsymbol{\iota}}\newcommand{\bikappa}{\boldsymbol{\kappa}}\newcommand{\bilambda}{\boldsymbol{\lambda}}\newcommand{\bimu}{\boldsymbol{\mu}}\newcommand{\binu}{\boldsymbol{\nu}}\newcommand{\bixi}{\boldsymbol{\xi}}\newcommand{\biomicron}{\boldsymbol{\micron}}\newcommand{\bipi}{\boldsymbol{\pi}}\newcommand{\birho}{\boldsymbol{\rho}}\newcommand{\bisigma}{\boldsymbol{\sigma}}\newcommand{\bitau}{\boldsymbol{\tau}}\newcommand{\biupsilon}{\boldsymbol{\upsilon}}\newcommand{\biphi}{\boldsymbol{\phi}}\newcommand{\bichi}{\boldsymbol{\chi}}\newcommand{\bipsi}{\boldsymbol{\psi}}\newcommand{\biomega}{\boldsymbol{\omega}}\left\| {{\bf{f}}\left( {\bf{x}} \right)} \right\| = \sqrt {\sum\limits_{\bf{x}} {{\bf{f}}{{\left( {\bf{x}} \right)}^2}} }$ equals 1.0.

The octave bandwidth of the preferred feature is given by the log-base-2 ratio of the high and low frequencies at half height:

\newcommand{\bialpha}{\boldsymbol{\alpha}}\newcommand{\bibeta}{\boldsymbol{\beta}}\newcommand{\bigamma}{\boldsymbol{\gamma}}\newcommand{\bidelta}{\boldsymbol{\delta}}\newcommand{\bivarepsilon}{\boldsymbol{\varepsilon}}\newcommand{\bizeta}{\boldsymbol{\zeta}}\newcommand{\bieta}{\boldsymbol{\eta}}\newcommand{\bitheta}{\boldsymbol{\theta}}\newcommand{\biiota}{\boldsymbol{\iota}}\newcommand{\bikappa}{\boldsymbol{\kappa}}\newcommand{\bilambda}{\boldsymbol{\lambda}}\newcommand{\bimu}{\boldsymbol{\mu}}\newcommand{\binu}{\boldsymbol{\nu}}\newcommand{\bixi}{\boldsymbol{\xi}}\newcommand{\biomicron}{\boldsymbol{\micron}}\newcommand{\bipi}{\boldsymbol{\pi}}\newcommand{\birho}{\boldsymbol{\rho}}\newcommand{\bisigma}{\boldsymbol{\sigma}}\newcommand{\bitau}{\boldsymbol{\tau}}\newcommand{\biupsilon}{\boldsymbol{\upsilon}}\newcommand{\biphi}{\boldsymbol{\phi}}\newcommand{\bichi}{\boldsymbol{\chi}}\newcommand{\bipsi}{\boldsymbol{\psi}}\newcommand{\biomega}{\boldsymbol{\omega}}\begin{equation}\tag{10}{{B}}{{{W}}_{{{oct}}}} = {\log _2}\left( {{{{f_H}} \over {{f_L}}}} \right) = {\log _2}\left( {{{{f_0} + {{\sqrt {\ln 4} } \mathord{\left/ {\vphantom {{\sqrt {\ln 4} } {2\pi {\sigma _b}}}} \right. \kern-1.2pt} {2\pi {\sigma _b}}}} \over {{f_0} - {{\sqrt {\ln 4} } \mathord{\left/ {\vphantom {{\sqrt {\ln 4} } {2\pi {\sigma _b}}}} \right. \kern-1.2pt} {2\pi {\sigma _b}}}}}} \right){\rm .}\end{equation}

The orientation bandwidth specifies the polar angle spanned by the Gaussian envelope at half height and is given by

\newcommand{\bialpha}{\boldsymbol{\alpha}}\newcommand{\bibeta}{\boldsymbol{\beta}}\newcommand{\bigamma}{\boldsymbol{\gamma}}\newcommand{\bidelta}{\boldsymbol{\delta}}\newcommand{\bivarepsilon}{\boldsymbol{\varepsilon}}\newcommand{\bizeta}{\boldsymbol{\zeta}}\newcommand{\bieta}{\boldsymbol{\eta}}\newcommand{\bitheta}{\boldsymbol{\theta}}\newcommand{\biiota}{\boldsymbol{\iota}}\newcommand{\bikappa}{\boldsymbol{\kappa}}\newcommand{\bilambda}{\boldsymbol{\lambda}}\newcommand{\bimu}{\boldsymbol{\mu}}\newcommand{\binu}{\boldsymbol{\nu}}\newcommand{\bixi}{\boldsymbol{\xi}}\newcommand{\biomicron}{\boldsymbol{\micron}}\newcommand{\bipi}{\boldsymbol{\pi}}\newcommand{\birho}{\boldsymbol{\rho}}\newcommand{\bisigma}{\boldsymbol{\sigma}}\newcommand{\bitau}{\boldsymbol{\tau}}\newcommand{\biupsilon}{\boldsymbol{\upsilon}}\newcommand{\biphi}{\boldsymbol{\phi}}\newcommand{\bichi}{\boldsymbol{\chi}}\newcommand{\bipsi}{\boldsymbol{\psi}}\newcommand{\biomega}{\boldsymbol{\omega}}\begin{equation}\tag{11}{{B}}{{{W}}_\theta } = 2{\tan ^{ - 1}}\left( {{{\sqrt {\ln 4} } \over {2\pi {\sigma _l}{f_0}}}} \right){\rm .}\end{equation}

When the visual angles spanned by the $\newcommand{\bialpha}{\boldsymbol{\alpha}}\newcommand{\bibeta}{\boldsymbol{\beta}}\newcommand{\bigamma}{\boldsymbol{\gamma}}\newcommand{\bidelta}{\boldsymbol{\delta}}\newcommand{\bivarepsilon}{\boldsymbol{\varepsilon}}\newcommand{\bizeta}{\boldsymbol{\zeta}}\newcommand{\bieta}{\boldsymbol{\eta}}\newcommand{\bitheta}{\boldsymbol{\theta}}\newcommand{\biiota}{\boldsymbol{\iota}}\newcommand{\bikappa}{\boldsymbol{\kappa}}\newcommand{\bilambda}{\boldsymbol{\lambda}}\newcommand{\bimu}{\boldsymbol{\mu}}\newcommand{\binu}{\boldsymbol{\nu}}\newcommand{\bixi}{\boldsymbol{\xi}}\newcommand{\biomicron}{\boldsymbol{\micron}}\newcommand{\bipi}{\boldsymbol{\pi}}\newcommand{\birho}{\boldsymbol{\rho}}\newcommand{\bisigma}{\boldsymbol{\sigma}}\newcommand{\bitau}{\boldsymbol{\tau}}\newcommand{\biupsilon}{\boldsymbol{\upsilon}}\newcommand{\biphi}{\boldsymbol{\phi}}\newcommand{\bichi}{\boldsymbol{\chi}}\newcommand{\bipsi}{\boldsymbol{\psi}}\newcommand{\biomega}{\boldsymbol{\omega}}x^{\prime}$ and $\newcommand{\bialpha}{\boldsymbol{\alpha}}\newcommand{\bibeta}{\boldsymbol{\beta}}\newcommand{\bigamma}{\boldsymbol{\gamma}}\newcommand{\bidelta}{\boldsymbol{\delta}}\newcommand{\bivarepsilon}{\boldsymbol{\varepsilon}}\newcommand{\bizeta}{\boldsymbol{\zeta}}\newcommand{\bieta}{\boldsymbol{\eta}}\newcommand{\bitheta}{\boldsymbol{\theta}}\newcommand{\biiota}{\boldsymbol{\iota}}\newcommand{\bikappa}{\boldsymbol{\kappa}}\newcommand{\bilambda}{\boldsymbol{\lambda}}\newcommand{\bimu}{\boldsymbol{\mu}}\newcommand{\binu}{\boldsymbol{\nu}}\newcommand{\bixi}{\boldsymbol{\xi}}\newcommand{\biomicron}{\boldsymbol{\micron}}\newcommand{\bipi}{\boldsymbol{\pi}}\newcommand{\birho}{\boldsymbol{\rho}}\newcommand{\bisigma}{\boldsymbol{\sigma}}\newcommand{\bitau}{\boldsymbol{\tau}}\newcommand{\biupsilon}{\boldsymbol{\upsilon}}\newcommand{\biphi}{\boldsymbol{\phi}}\newcommand{\bichi}{\boldsymbol{\chi}}\newcommand{\bipsi}{\boldsymbol{\psi}}\newcommand{\biomega}{\boldsymbol{\omega}}y^{\prime}$ values are 5 times the envelope standard deviations in the band-pass and low-pass directions, respectively, the weight matrix is matched to the preferred feature. When the spanned visual angles are greater than 5 times the standard deviation in either the band-pass or the low-pass direction, the weight matrix is mismatched to the receptive field.

We analyzed the response statistics of model neurons with vertically oriented Gabor receptive fields having 42° orientation bandwidths and 0.8-, 1.2-, 1.8-, and 2.4-octave bandwidths. Simple cells in early visual cortex have a median orientation bandwidth of 42° and a median octave bandwidth of 1.5 octaves. The distribution of cortical octave bandwidths spans approximately 0.8 to 2.4 octaves at half height (De Valois, Albrecht, & Thorell, 1982; De Valois, Yund, & Hepler, 1982; Ringach, 2002). We computed response statistics for mismatched receptive-field weight matrices spanning 5 times (e.g., 2 c/°, 72 pixels, 1.2°) to 20 times (e.g., 8 c/°, 72 pixels, 1.2°) the envelope standard deviations.

The aspect ratio of the Gaussian envelope in terms of the octave and orientation bandwidths is obtained by solving Equations 10 and 11 for $\newcommand{\bialpha}{\boldsymbol{\alpha}}\newcommand{\bibeta}{\boldsymbol{\beta}}\newcommand{\bigamma}{\boldsymbol{\gamma}}\newcommand{\bidelta}{\boldsymbol{\delta}}\newcommand{\bivarepsilon}{\boldsymbol{\varepsilon}}\newcommand{\bizeta}{\boldsymbol{\zeta}}\newcommand{\bieta}{\boldsymbol{\eta}}\newcommand{\bitheta}{\boldsymbol{\theta}}\newcommand{\biiota}{\boldsymbol{\iota}}\newcommand{\bikappa}{\boldsymbol{\kappa}}\newcommand{\bilambda}{\boldsymbol{\lambda}}\newcommand{\bimu}{\boldsymbol{\mu}}\newcommand{\binu}{\boldsymbol{\nu}}\newcommand{\bixi}{\boldsymbol{\xi}}\newcommand{\biomicron}{\boldsymbol{\micron}}\newcommand{\bipi}{\boldsymbol{\pi}}\newcommand{\birho}{\boldsymbol{\rho}}\newcommand{\bisigma}{\boldsymbol{\sigma}}\newcommand{\bitau}{\boldsymbol{\tau}}\newcommand{\biupsilon}{\boldsymbol{\upsilon}}\newcommand{\biphi}{\boldsymbol{\phi}}\newcommand{\bichi}{\boldsymbol{\chi}}\newcommand{\bipsi}{\boldsymbol{\psi}}\newcommand{\biomega}{\boldsymbol{\omega}}{\sigma _b}$ and $\newcommand{\bialpha}{\boldsymbol{\alpha}}\newcommand{\bibeta}{\boldsymbol{\beta}}\newcommand{\bigamma}{\boldsymbol{\gamma}}\newcommand{\bidelta}{\boldsymbol{\delta}}\newcommand{\bivarepsilon}{\boldsymbol{\varepsilon}}\newcommand{\bizeta}{\boldsymbol{\zeta}}\newcommand{\bieta}{\boldsymbol{\eta}}\newcommand{\bitheta}{\boldsymbol{\theta}}\newcommand{\biiota}{\boldsymbol{\iota}}\newcommand{\bikappa}{\boldsymbol{\kappa}}\newcommand{\bilambda}{\boldsymbol{\lambda}}\newcommand{\bimu}{\boldsymbol{\mu}}\newcommand{\binu}{\boldsymbol{\nu}}\newcommand{\bixi}{\boldsymbol{\xi}}\newcommand{\biomicron}{\boldsymbol{\micron}}\newcommand{\bipi}{\boldsymbol{\pi}}\newcommand{\birho}{\boldsymbol{\rho}}\newcommand{\bisigma}{\boldsymbol{\sigma}}\newcommand{\bitau}{\boldsymbol{\tau}}\newcommand{\biupsilon}{\boldsymbol{\upsilon}}\newcommand{\biphi}{\boldsymbol{\phi}}\newcommand{\bichi}{\boldsymbol{\chi}}\newcommand{\bipsi}{\boldsymbol{\psi}}\newcommand{\biomega}{\boldsymbol{\omega}}{\sigma _l}$, respectively, and then taking the ratio

\newcommand{\bialpha}{\boldsymbol{\alpha}}\newcommand{\bibeta}{\boldsymbol{\beta}}\newcommand{\bigamma}{\boldsymbol{\gamma}}\newcommand{\bidelta}{\boldsymbol{\delta}}\newcommand{\bivarepsilon}{\boldsymbol{\varepsilon}}\newcommand{\bizeta}{\boldsymbol{\zeta}}\newcommand{\bieta}{\boldsymbol{\eta}}\newcommand{\bitheta}{\boldsymbol{\theta}}\newcommand{\biiota}{\boldsymbol{\iota}}\newcommand{\bikappa}{\boldsymbol{\kappa}}\newcommand{\bilambda}{\boldsymbol{\lambda}}\newcommand{\bimu}{\boldsymbol{\mu}}\newcommand{\binu}{\boldsymbol{\nu}}\newcommand{\bixi}{\boldsymbol{\xi}}\newcommand{\biomicron}{\boldsymbol{\micron}}\newcommand{\bipi}{\boldsymbol{\pi}}\newcommand{\birho}{\boldsymbol{\rho}}\newcommand{\bisigma}{\boldsymbol{\sigma}}\newcommand{\bitau}{\boldsymbol{\tau}}\newcommand{\biupsilon}{\boldsymbol{\upsilon}}\newcommand{\biphi}{\boldsymbol{\phi}}\newcommand{\bichi}{\boldsymbol{\chi}}\newcommand{\bipsi}{\boldsymbol{\psi}}\newcommand{\biomega}{\boldsymbol{\omega}}\begin{equation}\tag{12}{{AR}} = {{{\sigma _l}} \over {{\sigma _b}}} = \cot \left( {{{{{B}}{{{W}}_\theta }} \over 2}} \right)\left( {{{{2^{{{B}}{{{W}}_{{{oct}}}}}} - 1} \over {{2^{{{B}}{{{W}}_{{{oct}}}}}} + 1}}} \right){\rm .}\end{equation}

The log-base-2 aspect ratios $\newcommand{\bialpha}{\boldsymbol{\alpha}}\newcommand{\bibeta}{\boldsymbol{\beta}}\newcommand{\bigamma}{\boldsymbol{\gamma}}\newcommand{\bidelta}{\boldsymbol{\delta}}\newcommand{\bivarepsilon}{\boldsymbol{\varepsilon}}\newcommand{\bizeta}{\boldsymbol{\zeta}}\newcommand{\bieta}{\boldsymbol{\eta}}\newcommand{\bitheta}{\boldsymbol{\theta}}\newcommand{\biiota}{\boldsymbol{\iota}}\newcommand{\bikappa}{\boldsymbol{\kappa}}\newcommand{\bilambda}{\boldsymbol{\lambda}}\newcommand{\bimu}{\boldsymbol{\mu}}\newcommand{\binu}{\boldsymbol{\nu}}\newcommand{\bixi}{\boldsymbol{\xi}}\newcommand{\biomicron}{\boldsymbol{\micron}}\newcommand{\bipi}{\boldsymbol{\pi}}\newcommand{\birho}{\boldsymbol{\rho}}\newcommand{\bisigma}{\boldsymbol{\sigma}}\newcommand{\bitau}{\boldsymbol{\tau}}\newcommand{\biupsilon}{\boldsymbol{\upsilon}}\newcommand{\biphi}{\boldsymbol{\phi}}\newcommand{\bichi}{\boldsymbol{\chi}}\newcommand{\bipsi}{\boldsymbol{\psi}}\newcommand{\biomega}{\boldsymbol{\omega}}{\log _2}\left( {{{AR}}} \right)$ of these receptive fields are −0.5, 0.0, 0.5, and 0.8, respectively, which correspond to envelopes that are, respectively, wider than they are high by a factor of $\newcommand{\bialpha}{\boldsymbol{\alpha}}\newcommand{\bibeta}{\boldsymbol{\beta}}\newcommand{\bigamma}{\boldsymbol{\gamma}}\newcommand{\bidelta}{\boldsymbol{\delta}}\newcommand{\bivarepsilon}{\boldsymbol{\varepsilon}}\newcommand{\bizeta}{\boldsymbol{\zeta}}\newcommand{\bieta}{\boldsymbol{\eta}}\newcommand{\bitheta}{\boldsymbol{\theta}}\newcommand{\biiota}{\boldsymbol{\iota}}\newcommand{\bikappa}{\boldsymbol{\kappa}}\newcommand{\bilambda}{\boldsymbol{\lambda}}\newcommand{\bimu}{\boldsymbol{\mu}}\newcommand{\binu}{\boldsymbol{\nu}}\newcommand{\bixi}{\boldsymbol{\xi}}\newcommand{\biomicron}{\boldsymbol{\micron}}\newcommand{\bipi}{\boldsymbol{\pi}}\newcommand{\birho}{\boldsymbol{\rho}}\newcommand{\bisigma}{\boldsymbol{\sigma}}\newcommand{\bitau}{\boldsymbol{\tau}}\newcommand{\biupsilon}{\boldsymbol{\upsilon}}\newcommand{\biphi}{\boldsymbol{\phi}}\newcommand{\bichi}{\boldsymbol{\chi}}\newcommand{\bipsi}{\boldsymbol{\psi}}\newcommand{\biomega}{\boldsymbol{\omega}}\sqrt 2$, circular, higher than they are wide by a factor of $\newcommand{\bialpha}{\boldsymbol{\alpha}}\newcommand{\bibeta}{\boldsymbol{\beta}}\newcommand{\bigamma}{\boldsymbol{\gamma}}\newcommand{\bidelta}{\boldsymbol{\delta}}\newcommand{\bivarepsilon}{\boldsymbol{\varepsilon}}\newcommand{\bizeta}{\boldsymbol{\zeta}}\newcommand{\bieta}{\boldsymbol{\eta}}\newcommand{\bitheta}{\boldsymbol{\theta}}\newcommand{\biiota}{\boldsymbol{\iota}}\newcommand{\bikappa}{\boldsymbol{\kappa}}\newcommand{\bilambda}{\boldsymbol{\lambda}}\newcommand{\bimu}{\boldsymbol{\mu}}\newcommand{\binu}{\boldsymbol{\nu}}\newcommand{\bixi}{\boldsymbol{\xi}}\newcommand{\biomicron}{\boldsymbol{\micron}}\newcommand{\bipi}{\boldsymbol{\pi}}\newcommand{\birho}{\boldsymbol{\rho}}\newcommand{\bisigma}{\boldsymbol{\sigma}}\newcommand{\bitau}{\boldsymbol{\tau}}\newcommand{\biupsilon}{\boldsymbol{\upsilon}}\newcommand{\biphi}{\boldsymbol{\phi}}\newcommand{\bichi}{\boldsymbol{\chi}}\newcommand{\bipsi}{\boldsymbol{\psi}}\newcommand{\biomega}{\boldsymbol{\omega}}\sqrt 2$, and higher than they are wide by a factor of $\newcommand{\bialpha}{\boldsymbol{\alpha}}\newcommand{\bibeta}{\boldsymbol{\beta}}\newcommand{\bigamma}{\boldsymbol{\gamma}}\newcommand{\bidelta}{\boldsymbol{\delta}}\newcommand{\bivarepsilon}{\boldsymbol{\varepsilon}}\newcommand{\bizeta}{\boldsymbol{\zeta}}\newcommand{\bieta}{\boldsymbol{\eta}}\newcommand{\bitheta}{\boldsymbol{\theta}}\newcommand{\biiota}{\boldsymbol{\iota}}\newcommand{\bikappa}{\boldsymbol{\kappa}}\newcommand{\bilambda}{\boldsymbol{\lambda}}\newcommand{\bimu}{\boldsymbol{\mu}}\newcommand{\binu}{\boldsymbol{\nu}}\newcommand{\bixi}{\boldsymbol{\xi}}\newcommand{\biomicron}{\boldsymbol{\micron}}\newcommand{\bipi}{\boldsymbol{\pi}}\newcommand{\birho}{\boldsymbol{\rho}}\newcommand{\bisigma}{\boldsymbol{\sigma}}\newcommand{\bitau}{\boldsymbol{\tau}}\newcommand{\biupsilon}{\boldsymbol{\upsilon}}\newcommand{\biphi}{\boldsymbol{\phi}}\newcommand{\bichi}{\boldsymbol{\chi}}\newcommand{\bipsi}{\boldsymbol{\psi}}\newcommand{\biomega}{\boldsymbol{\omega}}\sqrt 3$.

All data are presented for rectangular image patches and receptive-field weight matrices. However, there are practical disadvantages to working with matrices that are rectangular. It is more convenient to work with square patches and weight matrices. We examined how the response statistics differ between rectangular and square weight matrices. Note that nominally matched square matrices are actually slightly mismatched for octave bandwidths other than 1.2. We computed the response statistics with square image patches and weight matrices for all octave bandwidths. The differences were minor (Supplementary Figure S12).

### Normalization

To obtain the normalization factor for each stimulus, we converted each contrast image into its frequency-domain representation by performing a fast Fourier transform. Next, we normalized the transform such that its total power equaled the total energy of the contrast image, in accordance with Parseval's theorem. To prevent high-frequency artifacts that may be caused by the edge of the image patch, it is common to apply a cosine window before performing the fast Fourier transform. However, because of numerical issues, it is impossible to avoid occasionally exceeding the maximum response $\newcommand{\bialpha}{\boldsymbol{\alpha}}\newcommand{\bibeta}{\boldsymbol{\beta}}\newcommand{\bigamma}{\boldsymbol{\gamma}}\newcommand{\bidelta}{\boldsymbol{\delta}}\newcommand{\bivarepsilon}{\boldsymbol{\varepsilon}}\newcommand{\bizeta}{\boldsymbol{\zeta}}\newcommand{\bieta}{\boldsymbol{\eta}}\newcommand{\bitheta}{\boldsymbol{\theta}}\newcommand{\biiota}{\boldsymbol{\iota}}\newcommand{\bikappa}{\boldsymbol{\kappa}}\newcommand{\bilambda}{\boldsymbol{\lambda}}\newcommand{\bimu}{\boldsymbol{\mu}}\newcommand{\binu}{\boldsymbol{\nu}}\newcommand{\bixi}{\boldsymbol{\xi}}\newcommand{\biomicron}{\boldsymbol{\micron}}\newcommand{\bipi}{\boldsymbol{\pi}}\newcommand{\birho}{\boldsymbol{\rho}}\newcommand{\bisigma}{\boldsymbol{\sigma}}\newcommand{\bitau}{\boldsymbol{\tau}}\newcommand{\biupsilon}{\boldsymbol{\upsilon}}\newcommand{\biphi}{\boldsymbol{\phi}}\newcommand{\bichi}{\boldsymbol{\chi}}\newcommand{\bipsi}{\boldsymbol{\psi}}\newcommand{\biomega}{\boldsymbol{\omega}}{r_{\max }}$ when a window is applied. Stimulus contrast near the edge of the image patch that increases the linear response may be windowed out of the normalization factor. In this case, the normalization factor will be smaller than it should be. In some cases, it will cause the normalized response to exceed the maximum. Results were similar with and without windowing, but they were better behaved without it.

### Cross-orientation suppression

The normalization factor for real neurons in cortex often depends on image orientations at the location of the preferred feature inside the spatial-frequency passband but outside the orientation passband of the preferred feature. To model this, we created four colocalized receptive fields sharing the octave bandwidth and spatial frequency of the preferred feature but differing in orientation (0°, 45°, 90°, and 135°). These receptive fields effectively tile orientation space within the spatial-frequency passband of the preferred feature. Then we computed a narrowband normalization factor for each receptive field and performed a weighted sum. The normalization factor corresponding to the orientation preferred feature received a weight of 0.6, and those for the other three orientations received a total weight of 0.4. These weights result in a normalization index for cross-orientation suppression matching the average value in early visual cortex, as reported by Ruff, Alberts, and Cohen (2016).

### Surround suppression

The normalization factor for real neurons in cortex depends on stimulus contrast outside the classical receptive field. In the spirit of a recent report by Coen-Cagli, Kohn, and Schwartz (2015), we implemented a model that switches surround suppression on or off depending on whether the surround of an image is homogeneous or heterogeneous across space in the spatial-frequency band preferred by the classical receptive field. First we computed the narrowband normalization factor in each of the eight surround locations (Figure 9B). Then we computed the circular variance of the surround factors (Figure 9C, 9D). Last, if the variance of the surround factors was less than the median variance across the stimulus ensemble, the surround was labeled heterogeneous and surround suppression was turned off; if the variance was greater than the median variance, the surround was labeled homogeneous and surround suppression was turned on. When surround suppression was switched on, the surround contribution to the final normalization factor was obtained by weighting the normalization factor at each surround location according to its distance from the center of the image patch. The final normalization factor was obtained by averaging the surround contribution with the narrowband normalization factor computed over the image region spanned by the preferred feature of the receptive field.

### Encoding noise

The responses of neurons are noisy. If the same exact stimulus is presented multiple times, the neuron is likely to give a slightly different response to each presentation. We considered two types of encoding noise: constant additive noise and scaled additive noise. Both types were modeled as zero-mean Gaussian noise $\newcommand{\bialpha}{\boldsymbol{\alpha}}\newcommand{\bibeta}{\boldsymbol{\beta}}\newcommand{\bigamma}{\boldsymbol{\gamma}}\newcommand{\bidelta}{\boldsymbol{\delta}}\newcommand{\bivarepsilon}{\boldsymbol{\varepsilon}}\newcommand{\bizeta}{\boldsymbol{\zeta}}\newcommand{\bieta}{\boldsymbol{\eta}}\newcommand{\bitheta}{\boldsymbol{\theta}}\newcommand{\biiota}{\boldsymbol{\iota}}\newcommand{\bikappa}{\boldsymbol{\kappa}}\newcommand{\bilambda}{\boldsymbol{\lambda}}\newcommand{\bimu}{\boldsymbol{\mu}}\newcommand{\binu}{\boldsymbol{\nu}}\newcommand{\bixi}{\boldsymbol{\xi}}\newcommand{\biomicron}{\boldsymbol{\micron}}\newcommand{\bipi}{\boldsymbol{\pi}}\newcommand{\birho}{\boldsymbol{\rho}}\newcommand{\bisigma}{\boldsymbol{\sigma}}\newcommand{\bitau}{\boldsymbol{\tau}}\newcommand{\biupsilon}{\boldsymbol{\upsilon}}\newcommand{\biphi}{\boldsymbol{\phi}}\newcommand{\bichi}{\boldsymbol{\chi}}\newcommand{\bipsi}{\boldsymbol{\psi}}\newcommand{\biomega}{\boldsymbol{\omega}}\varepsilon \sim N\left( {0,\sigma _I^2} \right)$. With constant additive noise, the encoding noise variance $\newcommand{\bialpha}{\boldsymbol{\alpha}}\newcommand{\bibeta}{\boldsymbol{\beta}}\newcommand{\bigamma}{\boldsymbol{\gamma}}\newcommand{\bidelta}{\boldsymbol{\delta}}\newcommand{\bivarepsilon}{\boldsymbol{\varepsilon}}\newcommand{\bizeta}{\boldsymbol{\zeta}}\newcommand{\bieta}{\boldsymbol{\eta}}\newcommand{\bitheta}{\boldsymbol{\theta}}\newcommand{\biiota}{\boldsymbol{\iota}}\newcommand{\bikappa}{\boldsymbol{\kappa}}\newcommand{\bilambda}{\boldsymbol{\lambda}}\newcommand{\bimu}{\boldsymbol{\mu}}\newcommand{\binu}{\boldsymbol{\nu}}\newcommand{\bixi}{\boldsymbol{\xi}}\newcommand{\biomicron}{\boldsymbol{\micron}}\newcommand{\bipi}{\boldsymbol{\pi}}\newcommand{\birho}{\boldsymbol{\rho}}\newcommand{\bisigma}{\boldsymbol{\sigma}}\newcommand{\bitau}{\boldsymbol{\tau}}\newcommand{\biupsilon}{\boldsymbol{\upsilon}}\newcommand{\biphi}{\boldsymbol{\phi}}\newcommand{\bichi}{\boldsymbol{\chi}}\newcommand{\bipsi}{\boldsymbol{\psi}}\newcommand{\biomega}{\boldsymbol{\omega}}\sigma _I^2$ is constant regardless of the mean response. With scaled additive noise, the encoding noise variance $\newcommand{\bialpha}{\boldsymbol{\alpha}}\newcommand{\bibeta}{\boldsymbol{\beta}}\newcommand{\bigamma}{\boldsymbol{\gamma}}\newcommand{\bidelta}{\boldsymbol{\delta}}\newcommand{\bivarepsilon}{\boldsymbol{\varepsilon}}\newcommand{\bizeta}{\boldsymbol{\zeta}}\newcommand{\bieta}{\boldsymbol{\eta}}\newcommand{\bitheta}{\boldsymbol{\theta}}\newcommand{\biiota}{\boldsymbol{\iota}}\newcommand{\bikappa}{\boldsymbol{\kappa}}\newcommand{\bilambda}{\boldsymbol{\lambda}}\newcommand{\bimu}{\boldsymbol{\mu}}\newcommand{\binu}{\boldsymbol{\nu}}\newcommand{\bixi}{\boldsymbol{\xi}}\newcommand{\biomicron}{\boldsymbol{\micron}}\newcommand{\bipi}{\boldsymbol{\pi}}\newcommand{\birho}{\boldsymbol{\rho}}\newcommand{\bisigma}{\boldsymbol{\sigma}}\newcommand{\bitau}{\boldsymbol{\tau}}\newcommand{\biupsilon}{\boldsymbol{\upsilon}}\newcommand{\biphi}{\boldsymbol{\phi}}\newcommand{\bichi}{\boldsymbol{\chi}}\newcommand{\bipsi}{\boldsymbol{\psi}}\newcommand{\biomega}{\boldsymbol{\omega}}\sigma _I^2 = \alpha \left| r \right| + \sigma _0^2$ scales in rough proportion to the mean, where $\newcommand{\bialpha}{\boldsymbol{\alpha}}\newcommand{\bibeta}{\boldsymbol{\beta}}\newcommand{\bigamma}{\boldsymbol{\gamma}}\newcommand{\bidelta}{\boldsymbol{\delta}}\newcommand{\bivarepsilon}{\boldsymbol{\varepsilon}}\newcommand{\bizeta}{\boldsymbol{\zeta}}\newcommand{\bieta}{\boldsymbol{\eta}}\newcommand{\bitheta}{\boldsymbol{\theta}}\newcommand{\biiota}{\boldsymbol{\iota}}\newcommand{\bikappa}{\boldsymbol{\kappa}}\newcommand{\bilambda}{\boldsymbol{\lambda}}\newcommand{\bimu}{\boldsymbol{\mu}}\newcommand{\binu}{\boldsymbol{\nu}}\newcommand{\bixi}{\boldsymbol{\xi}}\newcommand{\biomicron}{\boldsymbol{\micron}}\newcommand{\bipi}{\boldsymbol{\pi}}\newcommand{\birho}{\boldsymbol{\rho}}\newcommand{\bisigma}{\boldsymbol{\sigma}}\newcommand{\bitau}{\boldsymbol{\tau}}\newcommand{\biupsilon}{\boldsymbol{\upsilon}}\newcommand{\biphi}{\boldsymbol{\phi}}\newcommand{\bichi}{\boldsymbol{\chi}}\newcommand{\bipsi}{\boldsymbol{\psi}}\newcommand{\biomega}{\boldsymbol{\omega}}\alpha$ is the Fano factor. All of the qualitative results are essentially invariant to whether constant or scaled additive noise is used. The article presents results for constant additive noise.

### Downsampling

Image patches were downsampled using MATLAB's imresize.m function with linear interpolation. Similar results are obtained using MATLAB's impyramid.m. However, with impyramid.m the downsampling factors are restricted to powers of 2; we favor imresize.m because of its increased flexibility. Other downsampling methods are likely to produce very similar results.

## Discussion

Model neurons employing narrowband response normalization yield scale-invariant, Gaussian-distributed response drives when stimulated with natural stimuli. The scale-invariant response-drive statistics nearly maximize the sensitivity for stimulus discrimination with natural stimuli, but the scale invariance depends on the normalization factor being determined from an image region that matches the size of the receptive field's preferred feature. In this section, we examine how these results are affected by receptive fields that are not oriented Gabors, normalization models that include cross-orientation and surround suppression, and stimulus types that are not natural (i.e., noise stimuli). We discuss how the results reported here can explain why subunit models fitted to neurons in cortex tend to poorly predict responses to natural stimuli, even when they beautifully predict responses to noise stimuli, and we show what our analyses of response-drive statistics predict about neural response to natural images.

### Generality of conclusions

Different subfields in vision and computational neuroscience have different methodological conventions for modeling neurons. Under many simplified circumstances, the different conventions have little or no practical impact. However, when the model neurons include the dominant features of real neurons in cortex—receptive field, response normalization, and encoding noise—the subtle differences in the modeling conventions can have a dramatic impact on response-drive statistics, especially when stimulated with natural images. How do modeling choices other than those considered in the body of this article impact the response-drive statistics?

First, we asked whether the response-drive statistics generalize to other receptive fields. So far, we have analyzed the response-drive statistics of vertically oriented even-symmetric (cosine-phase) Gabor receptive fields. Do odd-symmetric (sine-phase) receptive fields produce similar results? Yes. Gabor receptive fields with carriers of all phases and orientations produce equivalent results. Thus, model neurons with biologically plausible receptive fields and appropriate narrowband response normalization produce response statistics that are invariant to the preferred feature.

Next, we asked whether model neurons with other receptive-field shapes produce similar results. There is a long history of using Gabor functions to describe the receptive fields of simple cells in early visual cortex (Jones & Palmer, 1987a, 1987b), but empirical data suggest that log-Gabors may provide a better characterization of receptive fields in early visual cortex (De Valois, Albrecht, & Thorell, 1982; Geisler & Albrecht, 1997; Hawken & Parker, 1987). It has also been argued on theoretical grounds that log-Gabor receptive fields may be better than Gabor receptive fields for encoding natural images (D. J. Field, 1987). We reran our analyses with log-Gabor-shaped receptive fields; all results held (Supplementary Figure S7).

Then, we asked whether the main conclusions hold for the receptive fields like those of retinal ganglion cells or relay cells in the lateral geniculate nucleus, which are radially symmetric and do not select for orientation. We repeated our analyses with center–surround Gabors and difference-of-Gaussian preferred features embedded in matched weight matrices. With narrowband normalization, model neurons with oriented and unoriented receptive fields yield similar results. With broadband normalization, model neurons with unoriented receptive fields yield Gaussian response distributions (Supplementary Figure S8). (Recall that oriented receptive fields with broadband normalization yield Laplace-distributed responses; Figure 4C, 4D, Supplementary Figure S2.) This result implies that the differences in the shape of the response distributions that distinguish broadband and narrowband normalization with oriented Gabors (cf. Figure 4) are mostly due to the orientation selectivity of the receptive field.

### Cross-orientation suppression

**Figure 10:**
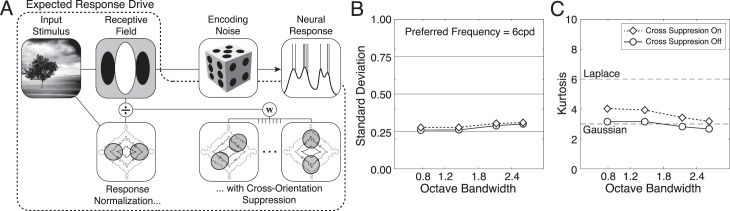
The effect of cross-orientation suppression on expected response drive. (A) Encoding model with cross-orientation suppression. The output of the linear filter is normalized by a factor computed from colocalized receptive fields differing in orientation but sharing the octave bandwidth and spatial-frequency preferences of the preferred feature. (B) Standard deviation of expected response drive. (C) Kurtosis of the expected response drive.

Real neurons in early visual cortex can be suppressed by stimulus contrast outside the orientation passband (but inside the spatial-frequency passband) of the receptive field (Carandini et al, 1997; Ruff et al., 2016). If the preferred feature is vertically oriented, for example, horizontal image orientations in the spatial region spanned by the preferred feature can contribute to the normalization factor. This is known as cross-orientation suppression. The strength of cross-orientation suppression varies substantially across individual cells (Ruff et al., 2016). We implemented a normalization model that matched the mean strength of cross-orientation suppression in early visual cortex (Ruff et al., 2016). In this model, the normalization factor was computed from a weighted sum of the image contrast inside and outside the orientation passband of the receptive field (Figure 10A); the weights were 0.6 and 0.4, respectively. With this form of cross-orientation suppression, the standard deviation of expected response drive decreases and the kurtosis increases (Figure 10B, 10C), but both of these effects are modest. For neurons exhibiting stronger cross-orientation suppression than the mean strength in cortex, the trend away from Gaussian response drives is likely to be more pronounced. For neurons having weaker cross-orientation suppression, the results will be more like those shown in Figure 7, in which the normalization factor did not include cross-orientation suppression.

### Surround suppression

**Figure 11:**
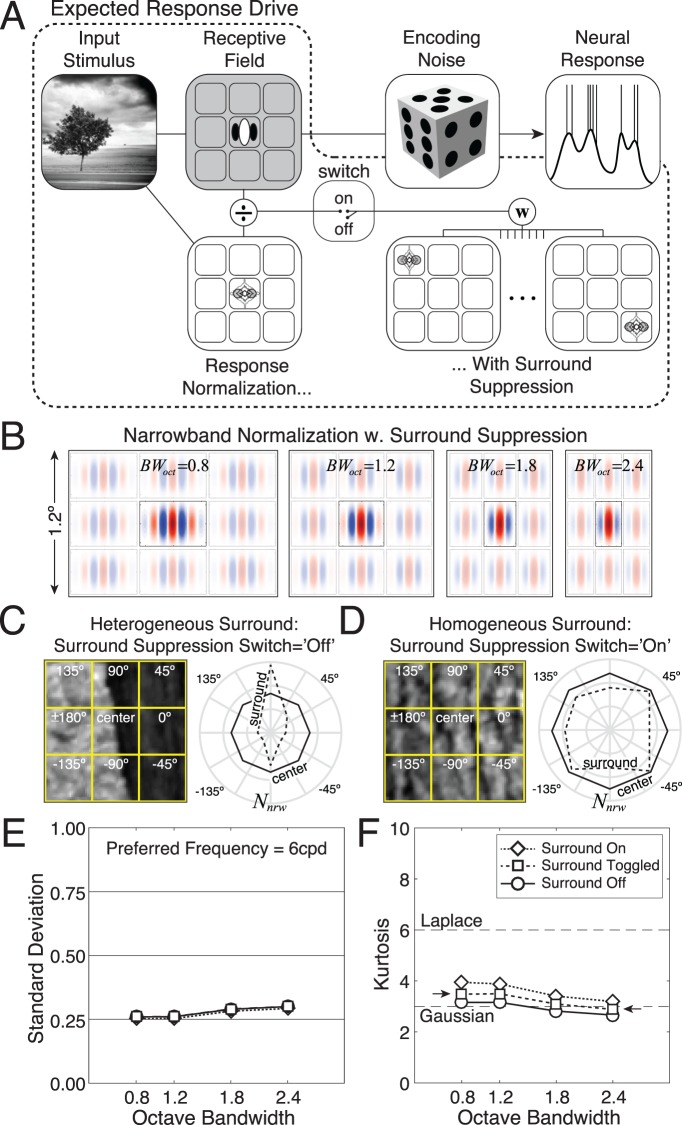
The effect of surround suppression on expected response drive. (A) Encoding model with surround suppression. The output of the linear filter is normalized by a normalization factor that is computed from the image region corresponding to the classical receptive field alone or in combination with surround suppression computed from the image region outside the classical receptive field. A narrowband normalization factor is computed in each of nine local image regions: eight from the surround and one from the center, corresponding to the preferred feature itself. Surround suppression is engaged (switch = on) if the surround is homogenous and disengaged (switch = off) if the surround is heterogeneous. (B) Spatial arrangement of classical receptive field (saturated colors) and surround (unsaturated colors). Examples with different octave bandwidths are shown. When surround suppression is engaged, the surround provides an equal contribution to the normalization factor as the image region spanned by the classical receptive field. (C) Example image with heterogeneous surround. The polar plot shows the narrowband normalization factors computed from each location in the surround (dashed curve) and from the center (solid curve). (D) Example image with homogeneous surround. (E) Standard deviation of expected response drive versus octave bandwidth with surround suppression on (diamonds), toggled (squares), and off (circles). (F) Kurtosis of expected response drive versus octave bandwidth with surround suppression on (diamonds), toggled (squares), and off (circles). Arrows mark the model most similar to that of Coen-Cagli, Kohn, and Schwartz (2015). Results are presented for a preferred spatial frequency of 6 c/°, and generalize to all spatial frequencies. Data with surround suppression off in (E–F) are identical to the data shown in Figure 8D–8E. Surround suppression causes a subtle but systematic increase on response kurtosis.

Just as real neurons in cortex can be suppressed by contrast at image orientations outside the orientation passband of the receptive field, real neurons can be suppressed by stimulus contrast outside the image region surrounding that spanned by the classical receptive field (Cavanaugh et al., 2002; Coen-Cagli et al., 2015). This is known as surround suppression. Earlier, we found that narrowband normalization fails to Gaussianize response drive when the normalization factor is determined from an image region much larger than the preferred feature (see Figure 7). But that form of surround suppression is broadband in both orientation and spatial frequency, whereas surround suppression in cortex is broadband in orientation but passband in spatial frequency. We modeled more realistic surround suppression to determine its impact on response-drive statistics. In this model, the normalization factor is derived from stimulus contrast inside the spatial-frequency passband of the preferred feature but outside the spatial region of the classical receptive field (Figure 11A). The model ensures that surround suppression is more pronounced when the stimulus contrast in the surround matches the preferred frequency and orientation of the preferred feature (Cavanaugh et al., 2002). Consistent with a recent proposal by Coen-Cagli et al. (2015), our model also uses a switch that toggles suppression on and off depending on the image properties of the surround region (Figure 11A through 11D; see Methods). When the surround was heterogeneous—that is, when the stimulus contrast in the spatial-frequency and orientation passband of the preferred feature varied substantially across the surround—surround suppression was turned off (Figure 11C). When the surround was homogeneous—that is, when the surround contrast in the passband was evenly distributed throughout the surround—surround suppression was turned on (Figure 11D).

Surround suppression of this form has a subtle but systematic effect on the statistics of response drive to the natural-image ensemble. The standard deviation of the expected response drive is largely unaffected (Figure 11E), but kurtosis increases modestly depending on whether surround suppression is off, toggled, or on (Figure 11F). It may be surprising that the surround suppression considered here does not have a more pronounced effect, given the dramatic changes caused by mismatched matrices in Figure 7E and 7F. In Figure 7, all stimulus contrast in the surround (i.e., the spatial region spanned by the weight matrix) contributed to the normalization factor, regardless of whether it was in the passband of the preferred feature. Here, only surround contrast in the spatial-frequency passband of the preferred feature contributes to the normalization factor. The fact that this form of surround suppression has only a modest effect on the response-drive statistics to the natural stimulus ensemble does not imply that surround suppression can be dispensed with in modeling the responses of real neurons in cortex. Indeed, to account for the diverse response properties of individual neurons, Coen-Cagli et al. (2015) had to adjust the strength of surround suppression on a neuron-by-neuron basis. The take-home point is that surround suppression that is broadly consistent with known neurophysiology is compatible with the statistics reported here.

### Natural versus noise stimuli

Model neurons with narrowband response normalization and matched weight matrices yield Gaussian-distributed responses to natural stimuli. However, noise stimuli are more often used in psychophysical and neurophysiological experiments. Noise stimuli have useful properties for methods designed to recover the stimulus features (i.e., receptive fields) that drive behavioral and neural response (Ahumada & Lovell, 1971; Schwartz, Pillow, Rust, & Simoncelli, 2006). 1/*f* noise has the amplitude spectrum but not the phase structure of natural images. White noise has neither the amplitude spectrum nor the phase structure of natural stimuli.

**Figure 12:**
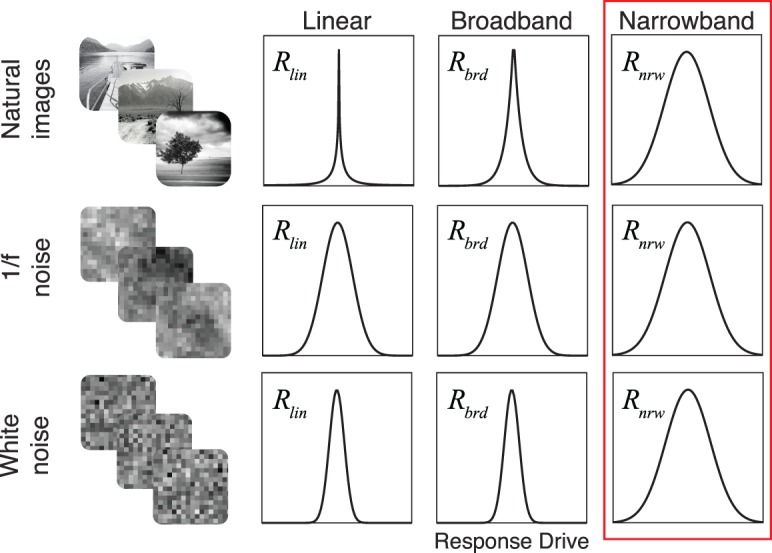
Distributions of linear, broadband-normalized, and narrowband-normalized response drives caused by natural stimuli, 1/f noise stimuli, and white-noise stimuli. Linear responses to natural stimuli are very heavy tailed (i.e., heavier than a Laplace distribution; see Supplementary Figure S1). Broadband responses to natural stimuli are Laplace distributed (see Supplementary Figure S2). Narrowband responses to natural stimuli are Gaussian (see Figures 5 and 8 and Supplementary Figure S3). Linear and broadband responses to 1/f and white noise are guaranteed to be Gaussian, if the number of pixels defining each stimulus (i.e., dimensionality) is sufficiently high. Narrowband responses to noise stimuli are all approximately Gaussian. Narrowband normalization produces responses (i.e., response drives) that are very nearly Gaussian with all three stimulus types. Narrowband normalization helps standardize the distributional form of the response statistics across stimulus types (box). Narrowband normalization should thus improve the ability of computational models of visual information processing to generalize across stimulus types.

We compared linear and normalized model-neuron response drives to natural stimuli, 1/*f* noise stimuli, and white-noise stimuli. As already shown, linear responses to natural stimuli are distributed with heavier tails than Laplace distributions. Broadband response drives to natural stimuli are Laplace distributed. Narrowband response drives to natural stimuli are approximately Gaussian (Supplementary Figures S1–S3). In contrast, linear, broadband, and narrowband response drives to 1/*f* and white-noise stimuli are all Gaussian distributed (Supplementary Figures S9 and S10). Thus, differences between linear, broadband, and narrowband response drives are negligible for noise stimuli but substantial for natural stimuli (Figure 12).

These findings may help explain why subunit models of neural response tend to generalize poorly when tested with natural stimuli. Subunit models are a popular method for performing neural-systems identification. Their aim is provide a concise computational-level description of the input-output relationship between stimulus and response. Subunit models are typically fitted and tested using noise stimuli. But while fitted subunit models nicely predict performance with noise stimuli, they tend to perform poorly when tested with natural stimuli (Heitman et al., 2016; Smyth, Willmore, Baker, Thompson, & Tolhurst, 2003; Talebi & Baker, 2012).

Many subunit models of neural response use quadratic pooling (McFarland, Cui, & Butts, 2013; I. M. Park, Archer, Priebe, & Pillow, 2013; Rust, Schwartz, Movshon, & Simoncelli, 2005; Schwartz et al., 2006; Vintch, Movshon, & Simoncelli, 2015; Wu, Park, & Pillow, 2015). Most do not incorporate response normalization (but see McFarland et al., 2013). If stimuli preferred by a neuron elicit jointly Gaussian response drives across subunits, subunit models with quadratic pooling will be able to make use of all of the information useful for predicting the neuron's membrane potential or spiking response. On the other hand, if the preferred stimuli elicit non-Gaussian response drives, subunit models with quadratic pooling will generally not be able to use all of the available information for predicting response.

Subunit models are most often fitted and tested with Gaussian noise stimuli. With Gaussian noise stimuli, linear (unnormalized) response models are guaranteed to yield Gaussian response drives. Broadband-normalized response drives are also guaranteed to be Gaussian, assuming that the number of pixels defining each stimulus—i.e., the dimensionality of each stimulus—is sufficiently high (Poincaré, 1912). The absence of narrowband response normalization thus has little practical effect with the stimuli with which subunit models are most often fitted and tested.

However, a natural-stimulus ensemble that would elicit Gaussian-distributed subunit response drives with narrowband normalization fails to do so without it. The absence of narrowband normalization causes response drives that are highly non-Gaussian (i.e., heavy tailed or sparse). This contributes to the poor generalization of subunit-model predictions to natural stimuli. Incorporating narrowband normalization into these methods for neural-systems identification will make the distributions of response drive more similar for both natural and noise stimuli, and may substantially improve the ability of these models to generalize across stimulus types.

### Nonparametric receptive-field learning: Model-neuron modeling conventions

Some areas of computational neuroscience aim to learn populations of receptive fields (i.e., preferred features) that optimize a particular goal. The preferred features of these receptive fields are typically learned by iteratively updating coefficients (i.e., pixel values) in pursuit of the goal. The receptive-field weight matrices typically have a fixed number of pixels and span a fixed visual angle. This convention is convenient for matrix-based programming languages (e.g., MATLAB; MathWorks, Natick, MA), but it often results in weight matrices that are mismatched to the preferred feature. Mismatched matrices are commonly reported by articles that fit subunit models to neural activity (see figure 2a in Rust et al., 2005; McFarland et al., 2013; I. M. Park et al., 2013; Samengo & Gollisch, 2013; Schwartz et al., 2006; Vintch et al., 2015; Wu et al., 2015), and by articles that seek receptive-field populations that efficiently encode natural stimuli (see figure 4a in Olshausen & Field, 1996; Bell & Sejnowski, 1997; Lewicki, 2002; Olshausen & Field, 1997; Rehn & Sommer, 2007). Why are mismatched weight matrices commonly reported if they carry the disadvantages detailed under Results (see Figure 8)? The literature mentioned here typically assumes that receptive-field (or subunit) responses are driven purely by linear operations and do not incorporate response normalization. (They also do not typically model encoding noise.) In the absence of normalization, matched and mismatched matrices yield identical responses (see Results).

To increase biological realism and to facilitate generalization to natural stimuli, normalization should be included in the response models for these feature-learning methods and others. If normalization is included without a matched weight matrix (i.e., the normalization factor is not computed from the same image region spanned by the preferred feature), receptive fields with preferred features smaller than the visual angle spanned by the weight matrix will have poor sensitivity (Figures 7 and 8). If small, high-frequency features are not learned, it will be unclear whether this is because of poor sensitivity caused by the modeling convention or because high-frequency filters are fundamentally not useful. If receptive fields within a parametric family are being learned (e.g., Gabor), the parameters can be used to determine the area spanned by the feature, but this is not possible with nonparametric approaches. Another way to address this issue would be to learn receptive fields with localized priors, but this technique poses significant technical challenges in many contexts (M. Park & Pillow, 2011). A simpler approach would be to learn receptive fields with response normalization at multiple scales simultaneously. At each scale, receptive fields with small mismatched preferred features will yield poor sensitivity, biasing the learning procedures against selecting those features relative to the scale. Across scale, however, large and small features would both be given a fair chance; large features would be matched to large matrices and small features to small matrices.

### Neural response in early visual cortex

The results presented here indicate that it is exceedingly rare for a neuron to be stimulated by its preferred feature in natural scenes. The standard deviation of stimulus-driven response equals approximately 25% of the maximum response, which means that less than one natural stimulus in 10,000 will cause a response within 5% of the neuron's maximum response. How should these results inform our thinking about neurophysiological processing of real-world signals? The majority of single-unit neurophysiology has focused on characterizing the stimuli to which individual neurons respond most strongly. We owe much of our knowledge about the response properties of V1 neurons to this approach. But real-world stimuli only very rarely drive neurons to response at maximum. Also, during large-scale simultaneous recordings of multiple neurons, it is impossible to simultaneously target each recorded neuron with its preferred stimulus. To understand the coding problems faced by nervous systems in natural viewing, it is critical to understand how neurons are driven by the stimulus ensemble encountered in the real world.

**Figure 13:**
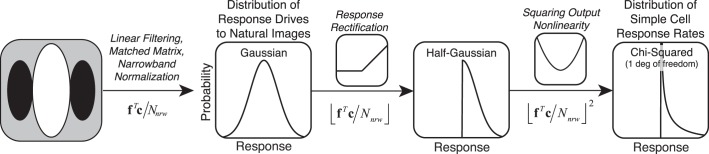
Relating Gaussian response-drive statistics to the statistics of spiking response in cortex. Response drives are first rectified, creating a half-Gaussian distribution. Rectified response drives are then squared, creating a chi-square distribution. The predicted distribution of simple-cell response rates in early visual cortex is the chi-square distribution with one degree of freedom. The predicted distribution of complex-cell response rates in early visual cortex is the chi-square distribution with two degree of freedom (see text).

Across many natural images, model-neuron response drive is zero-mean and Gaussian distributed. The response drive is equivalent to the neural response under the assumptions that no response rectification occurs and that the power of the static output nonlinearity is 1.0. In real neurons these assumptions do not typically hold. As a consequence, the responses of real neurons in early visual cortex to natural stimuli are typically not Gaussian (Felsen et al., 2005; Weliky et al., 2003). Rather, neural response is typically obtained by rectifying response drive and then squaring with a static output nonlinearity having a power of 2.0 (Priebe & Ferster, 2008). We examined how rectification and a squaring output nonlinearity change the model-neuron response statistics. Rectifying and squaring the response drive converts the Gaussian response distributions into chi-square response distributions with one degree of freedom, $\newcommand{\bialpha}{\boldsymbol{\alpha}}\newcommand{\bibeta}{\boldsymbol{\beta}}\newcommand{\bigamma}{\boldsymbol{\gamma}}\newcommand{\bidelta}{\boldsymbol{\delta}}\newcommand{\bivarepsilon}{\boldsymbol{\varepsilon}}\newcommand{\bizeta}{\boldsymbol{\zeta}}\newcommand{\bieta}{\boldsymbol{\eta}}\newcommand{\bitheta}{\boldsymbol{\theta}}\newcommand{\biiota}{\boldsymbol{\iota}}\newcommand{\bikappa}{\boldsymbol{\kappa}}\newcommand{\bilambda}{\boldsymbol{\lambda}}\newcommand{\bimu}{\boldsymbol{\mu}}\newcommand{\binu}{\boldsymbol{\nu}}\newcommand{\bixi}{\boldsymbol{\xi}}\newcommand{\biomicron}{\boldsymbol{\micron}}\newcommand{\bipi}{\boldsymbol{\pi}}\newcommand{\birho}{\boldsymbol{\rho}}\newcommand{\bisigma}{\boldsymbol{\sigma}}\newcommand{\bitau}{\boldsymbol{\tau}}\newcommand{\biupsilon}{\boldsymbol{\upsilon}}\newcommand{\biphi}{\boldsymbol{\phi}}\newcommand{\bichi}{\boldsymbol{\chi}}\newcommand{\bipsi}{\boldsymbol{\psi}}\newcommand{\biomega}{\boldsymbol{\omega}}\sigma _E^2\chi _1^2$, scaled by the stimulus-driven response variance (Figure 13). The scaled chi-square is more similar to the response distributions observed from spiking neurons in cortex. The scaled chi-square distribution is equivalently described by a gamma distribution with shape and scale parameters of 1/2 and $\newcommand{\bialpha}{\boldsymbol{\alpha}}\newcommand{\bibeta}{\boldsymbol{\beta}}\newcommand{\bigamma}{\boldsymbol{\gamma}}\newcommand{\bidelta}{\boldsymbol{\delta}}\newcommand{\bivarepsilon}{\boldsymbol{\varepsilon}}\newcommand{\bizeta}{\boldsymbol{\zeta}}\newcommand{\bieta}{\boldsymbol{\eta}}\newcommand{\bitheta}{\boldsymbol{\theta}}\newcommand{\biiota}{\boldsymbol{\iota}}\newcommand{\bikappa}{\boldsymbol{\kappa}}\newcommand{\bilambda}{\boldsymbol{\lambda}}\newcommand{\bimu}{\boldsymbol{\mu}}\newcommand{\binu}{\boldsymbol{\nu}}\newcommand{\bixi}{\boldsymbol{\xi}}\newcommand{\biomicron}{\boldsymbol{\micron}}\newcommand{\bipi}{\boldsymbol{\pi}}\newcommand{\birho}{\boldsymbol{\rho}}\newcommand{\bisigma}{\boldsymbol{\sigma}}\newcommand{\bitau}{\boldsymbol{\tau}}\newcommand{\biupsilon}{\boldsymbol{\upsilon}}\newcommand{\biphi}{\boldsymbol{\phi}}\newcommand{\bichi}{\boldsymbol{\chi}}\newcommand{\bipsi}{\boldsymbol{\psi}}\newcommand{\biomega}{\boldsymbol{\omega}}2\sigma _E^2$, respectively.

These considerations make several predictions. First, presenting natural stimuli to simple cells in early visual cortex should yield response-rate distributions that are nicely modeled by scaled chi-square distributions with one degree of freedom. Second, because complex cells are typically modeled as resulting from quadratic pooling of two (or more) subunit receptive-field responses, complex cells should yield response-rate distributions that are well modeled by scaled chi-square distributions with two (or more) degrees of freedom. Third, the distribution of response across natural stimuli for different neurons should be approximately invariant to the preferred feature of each neuron. It would be useful to compare the recorded distribution of neural spiking response for thousands of natural images to chi-square distributions with variable degrees of freedom.

Fortunately, some data relevant to these predictions are available. Baddeley et al. (1997) reported spike-response distributions to natural-image movies from cells in early visual cortex of the cat, although they did not report whether the cells were simple or complex. As predicted by the current modeling efforts, the spiking responses across natural images in their data are well described by chi-square distributions (Supplementary Figure S11). Some cells are best described by chi-square distributions with one degree of freedom, as should occur with simple cells. Others are best described by chi-square distributions with two degrees of freedom, as should occur with complex cells. Baddeley et al. these distributions as exponentials, partly because of considerations from the efficient-coding hypothesis. However, an exponential distribution is simply a chi-square distribution with two degrees of freedom. Thus, the results presented here are not inconsistent with observed neurophysiological data. In fact, our results may help explain observations of spiking response in cortex. Gaussian response-drive statistics, together with the nonlinearities employed by simple and complex cells in cortex, predict that spiking responses should be approximately chi-square distributed. The modeling efforts reported here show how and why those response patterns may emerge.

It is also important to ask how sensitivity for stimulus discrimination is impacted by rectifying and squaring the response drive. If the controlling source of encoding noise occurs after rectification and squaring, sensitivity will be altered (Equation 13A). On the other hand, if the controlling source of encoding noise is added at the level of the response drive, before the rectification and squaring (Equation 13B), sensitivity for stimulus discrimination will be unaffected. Transforming a noisy signal monotonically does not alter sensitivity (Pelli, 1985; Rieke & Rudd, 2009; Tanner, 1961).

\newcommand{\bialpha}{\boldsymbol{\alpha}}\newcommand{\bibeta}{\boldsymbol{\beta}}\newcommand{\bigamma}{\boldsymbol{\gamma}}\newcommand{\bidelta}{\boldsymbol{\delta}}\newcommand{\bivarepsilon}{\boldsymbol{\varepsilon}}\newcommand{\bizeta}{\boldsymbol{\zeta}}\newcommand{\bieta}{\boldsymbol{\eta}}\newcommand{\bitheta}{\boldsymbol{\theta}}\newcommand{\biiota}{\boldsymbol{\iota}}\newcommand{\bikappa}{\boldsymbol{\kappa}}\newcommand{\bilambda}{\boldsymbol{\lambda}}\newcommand{\bimu}{\boldsymbol{\mu}}\newcommand{\binu}{\boldsymbol{\nu}}\newcommand{\bixi}{\boldsymbol{\xi}}\newcommand{\biomicron}{\boldsymbol{\micron}}\newcommand{\bipi}{\boldsymbol{\pi}}\newcommand{\birho}{\boldsymbol{\rho}}\newcommand{\bisigma}{\boldsymbol{\sigma}}\newcommand{\bitau}{\boldsymbol{\tau}}\newcommand{\biupsilon}{\boldsymbol{\upsilon}}\newcommand{\biphi}{\boldsymbol{\phi}}\newcommand{\bichi}{\boldsymbol{\chi}}\newcommand{\bipsi}{\boldsymbol{\psi}}\newcommand{\biomega}{\boldsymbol{\omega}}\begin{equation}\tag{13A}r = {r_{\max }}{\left\lfloor {\overbrace {\left[ {{{{{\bf{f}}^{T}}{\bf{c}}} \over N}} \right]}^{{\rm{expected\ response\ drive}}}} \right\rfloor ^p} + \varepsilon \end{equation}

\newcommand{\bialpha}{\boldsymbol{\alpha}}\newcommand{\bibeta}{\boldsymbol{\beta}}\newcommand{\bigamma}{\boldsymbol{\gamma}}\newcommand{\bidelta}{\boldsymbol{\delta}}\newcommand{\bivarepsilon}{\boldsymbol{\varepsilon}}\newcommand{\bizeta}{\boldsymbol{\zeta}}\newcommand{\bieta}{\boldsymbol{\eta}}\newcommand{\bitheta}{\boldsymbol{\theta}}\newcommand{\biiota}{\boldsymbol{\iota}}\newcommand{\bikappa}{\boldsymbol{\kappa}}\newcommand{\bilambda}{\boldsymbol{\lambda}}\newcommand{\bimu}{\boldsymbol{\mu}}\newcommand{\binu}{\boldsymbol{\nu}}\newcommand{\bixi}{\boldsymbol{\xi}}\newcommand{\biomicron}{\boldsymbol{\micron}}\newcommand{\bipi}{\boldsymbol{\pi}}\newcommand{\birho}{\boldsymbol{\rho}}\newcommand{\bisigma}{\boldsymbol{\sigma}}\newcommand{\bitau}{\boldsymbol{\tau}}\newcommand{\biupsilon}{\boldsymbol{\upsilon}}\newcommand{\biphi}{\boldsymbol{\phi}}\newcommand{\bichi}{\boldsymbol{\chi}}\newcommand{\bipsi}{\boldsymbol{\psi}}\newcommand{\biomega}{\boldsymbol{\omega}}\begin{equation}\tag{13B}r = {r_{\max }}{\left\lfloor {\overbrace {\left[ {{{{{\bf{f}}^{T}}{\bf{c}}} \over N}} \right]}^{{\rm{expected\ response\ drive}}} + \varepsilon } \right\rfloor ^p} \end{equation}

A number of influential models propose that the controlling source of encoding noise is at the level of the membrane voltage, and should be modeled as constant, additive, and zero mean (Carandini, 2004; Mohanty, Scholl, & Priebe, 2012; Priebe & Ferster, 2008). After rectification and squaring, the noisy voltage signal predicts Poisson-like encoding noise (i.e., response variance that scales with the mean response to each stimulus), similar to typical observations in cortex (Tolhurst, Movshon, & Dean, 1983). If this is correct, the controlling encoding noise should be modeled as occurring at the level of the response drive (Equation 13B). Lastly, these considerations make one additional prediction. If the intracellular voltage reflects the response drive as it has been discussed in this article, the distribution of intracellular voltages across the natural-stimulus ensemble should be Gaussian distributed (Tan, Chen, Scholl, Seidemann, & Priebe, 2014).

### Task-specific analysis of natural images and scenes

Efficient coding is an influential theoretical framework for thinking about how neural systems encode natural stimuli (Attneave, 1954; Barlow, 2001). Many articles have focused on learning receptive fields that efficiently reconstruct proximal stimuli (Bell & Sejnowski, 1997; Lewicki, 2002; Olshausen & Field, 1996, 1997). However, the sensory-perceptual systems of humans and other animals must do more than efficiently encode and reconstruct sensory inputs. They must extract information from stimuli that is useful for the behavioral tasks that organisms must perform to survive and reproduce. Efficient coding does not directly address this problem.

In recent years, statistical techniques have been developed that learn small populations of receptive fields that encode the stimulus features most useful for specific tasks (Burge & Jaini, 2017; Geisler, Najemnik, & Ing, 2009; Jaini & Burge, 2017). These techniques have helped to find the optimal solutions to perceptual subproblems useful for estimating the three-dimensional structure of the environment—focus-error estimation (Burge & Geisler, 2011, 2012), disparity estimation (Burge & Geisler, 2014), and retinal-motion estimation (Burge & Geisler, 2015)—that have been the focus of intense study for decades in the vision and neuroscience communities. In this article, we examined only the response statistics of individual model neurons and considered only performance in a very simple task: discriminating one stimulus from another. The optimal solutions to these more sophisticated tasks require combining the responses from multiple receptive fields. With appropriate normalization, response drives to natural stimuli from multiple receptive fields are jointly Gaussian, a fact that should simplify computations for optimally combining those receptive-field responses. Thus, the results reported here should be thought of as the beginning of a more complete investigation of how visual systems process natural stimuli. Having an accurate picture of the response-drive statistics of model neurons and understanding how small differences in modeling conventions affect those statistics lay a foundation for building principled models in the future.

## Conclusion

The work presented in this article suggests that incorporating narrowband (i.e., stimulus- and feature-specific) response normalization will benefit computational models of natural-image processing. A simple expression for computing the narrowband normalization factor is provided that should facilitate the inclusion of narrowband normalization in nonparametric methods for learning receptive fields of model-neuron-like units. Narrowband normalization should also improve the ability of such models to generalize from noise stimuli to natural stimuli, because narrowband normalization yields scale-invariant Gaussian response drives with both stimulus types.

## Supplementary Material

Supplement 1
