# Supplementary material for: The statistics of how natural images drive the responses of neurons
Source: J Vis. 2019 Nov 5;19(13):4. doi: 10.1167/19.13.4 (PMC6833984; doi:10.1167/19.13.4)
Supplement: Supplement 1 [file jovi-19-11-09_s01.pdf]

Supplement

The statistics of how natural images drive the responses of neurons

Arvind Iyer[1.] & Johannes Burge[1.,2.,3.*]

[1.] Department of Psychology, University of Pennsylvania, Philadelphia PA, USA
[2.] Neuroscience Graduate Group, University of Pennsylvania, Philadelphia PA, USA
[3.] Bioengineering Graduate Group, University of Pennsylvania, Philadelphia PA, US

[*.] Corresponding author: Johannes Burge (jburge@sas.upenn.edu)

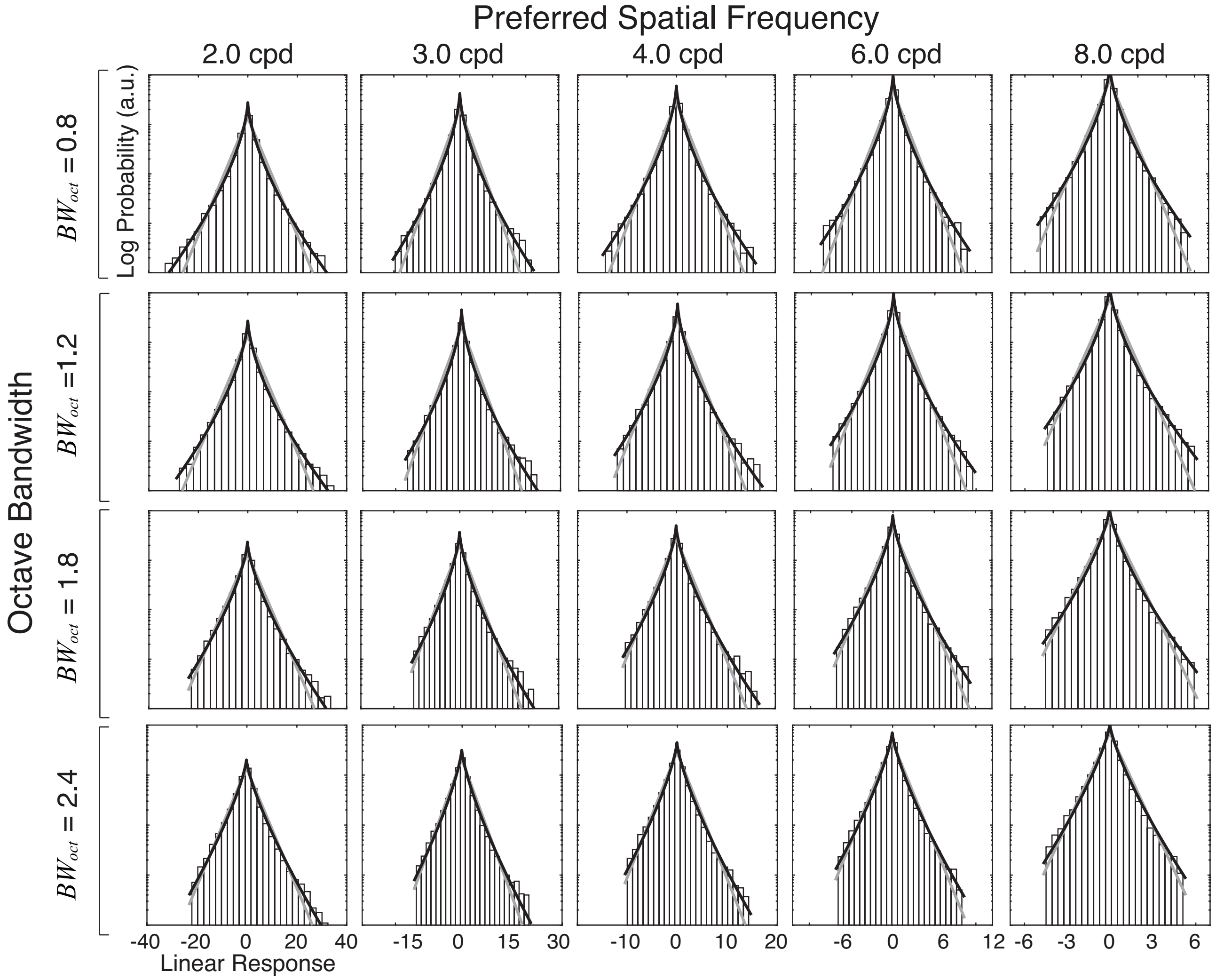

Figure S1. Linear response drives to natural images for all preferred spatial frequencies (columns) and octave bandwidths (rows). Note the dramatic difference in response magnitude (x-axis) as a function of preferred frequency. The responses are fit with both a Laplace and a generalized Gaussian via maximum likelihood (gray & black curves, respectively). The best-fit generalized Gaussian fit has significantly heavier tails than the best fit Laplace in all cases. The generalized Gaussian is given by $p(R) \propto \exp\left[-\left|\frac{R}{\sqrt{2}\sigma}\right|^p\right]$ where the Laplace distribution (p =1.0) and the Gaussian (p =2.0) are special cases. The linear responses are best fit with powers p of between 0.62 and 0.70, with a mean power of 0.65. The y-axis indicates log-probability over a four order-of-magnitude range. A Laplace distribution appears as straight lines on a log-probability plot.

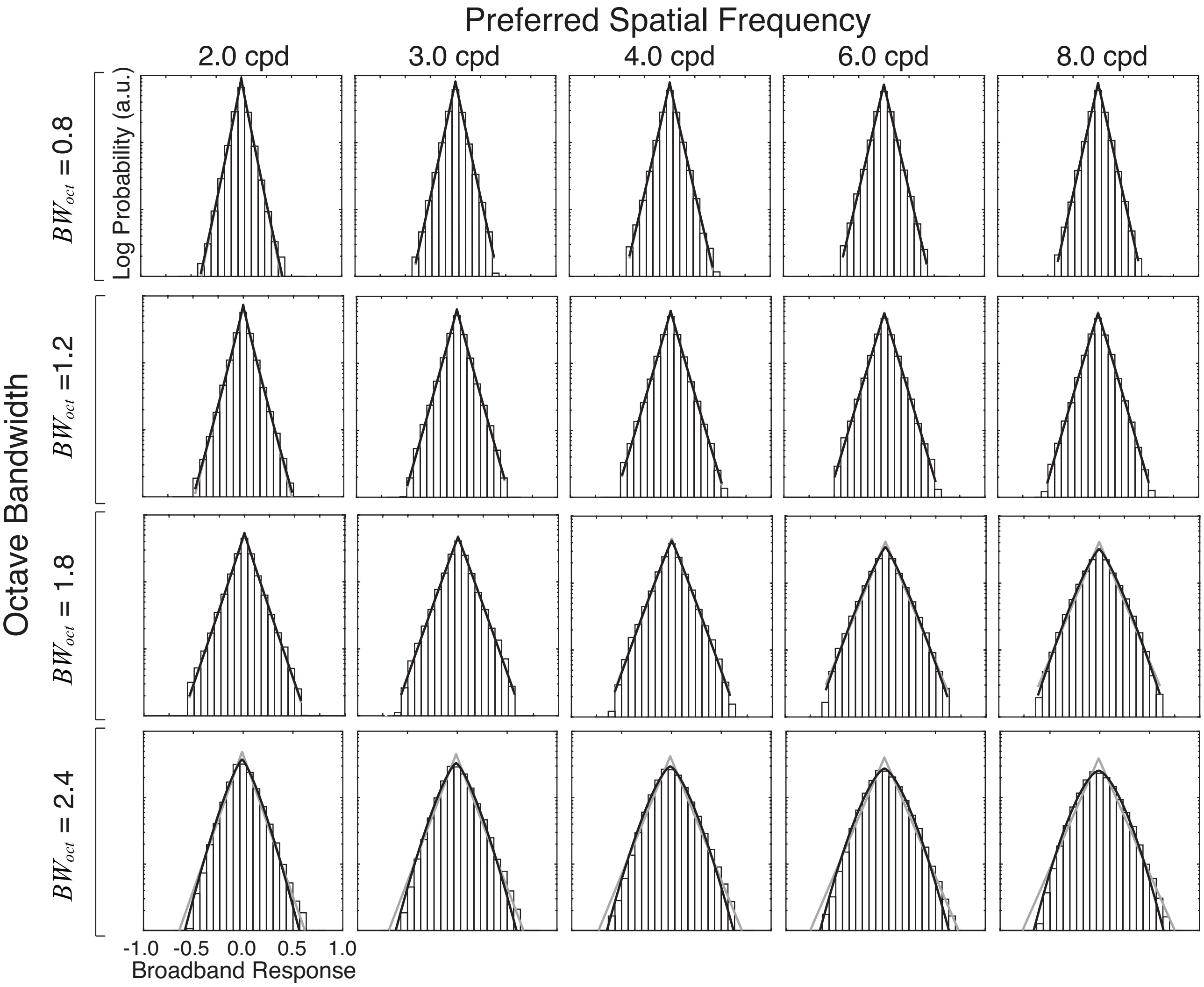

Figure S2. Broadband response drives to natural images are Laplace distributed. Broadband response drives with matched receptive field weight matrices for all preferred spatial frequencies (columns) and octave bandwidths (rows). The response drives are fit with both a Laplace and a generalized Gaussian via maximum likelihood (gray & black curves, respectively). The generalized Gaussian is given by $p(R) \propto \exp\left[-\left|\frac{R}{\sqrt{2}\sigma}\right|^{p}\right]$ where the Laplace distribution (p =1.0) and the Gaussian (p =2.0) are special cases. The generalized Gaussian fit is indistinguishable from the Laplace fit in almost all cases; when the gray lines are not visible, it is because they are behind the black curve. The y-axis indicates log-probability over a three order-of-magnitude range. The Laplace fit appears as straight lines on a log-probability plot.

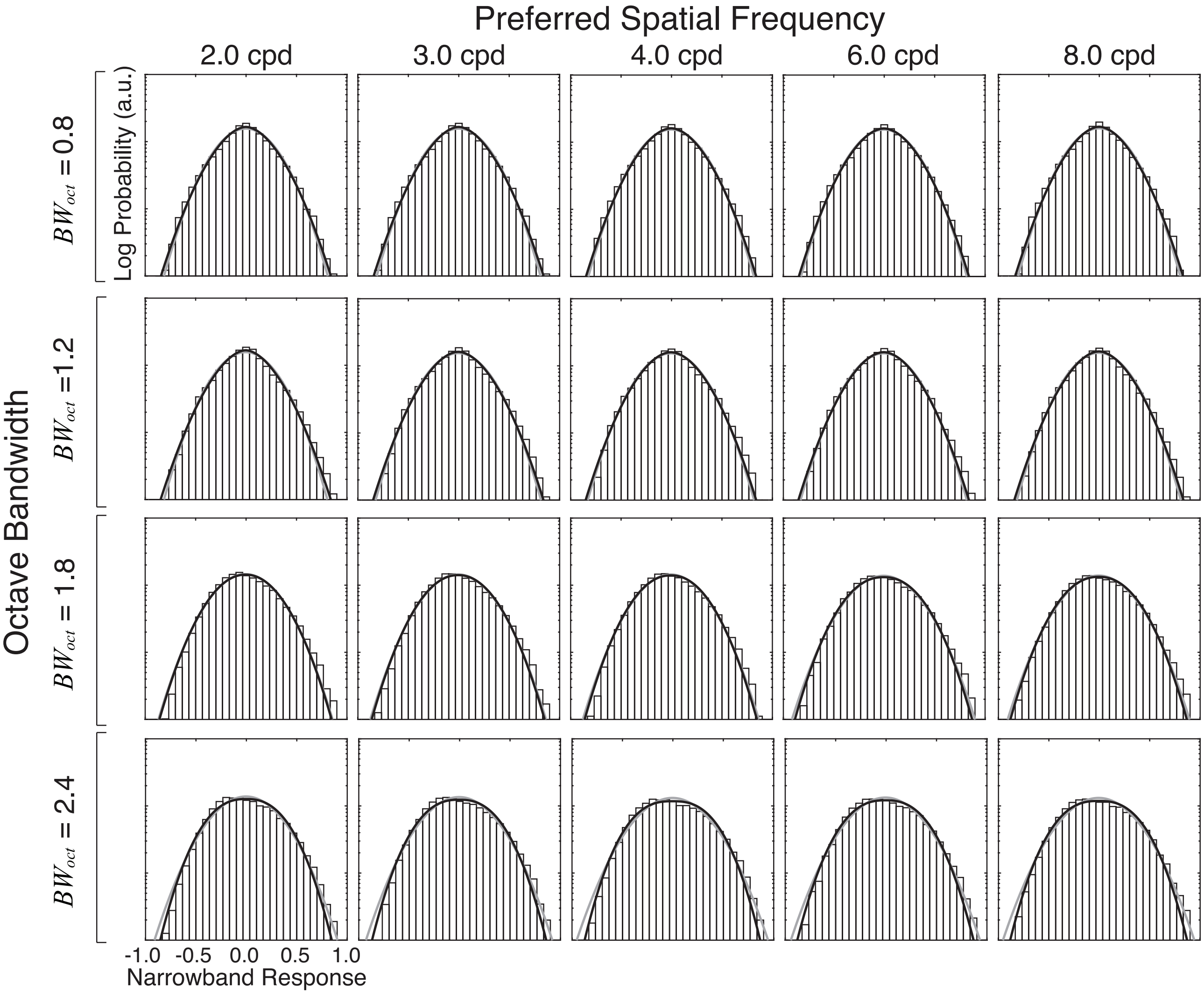

Figure S3. Narrowband response drives to natural images are Gaussian-distributed. Narrowband response drives with matched receptive field weight matrices for all preferred spatial frequencies (columns) and octave bandwidths (rows). The response drives are fit with both a Gaussian and a generalized Gaussian via maximum likelihood (gray & black curves, respectively). The generalized Gaussian is given by $p(R) \propto \exp\left[-\left|\frac{R}{\sqrt{2}\sigma}\right|^{p}\right]$ where the Laplace distribution (p =1.0) and the Gaussian (p =2.0) are special cases. The generalized Gaussian fit is indistinguishable from the Gaussian fit in almost all cases; when the gray lines are not visible, it is because they are behind the black curve. The y-axis indicates log-probability over a three order-of-magnitude range. The Gaussian distribution appears as an upside-down parabola on a log-probability plot.

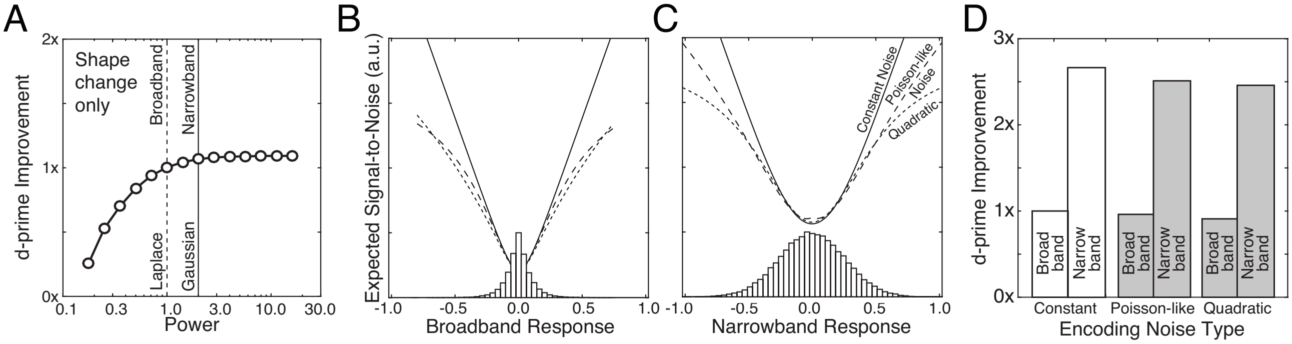

Figure S4. The impact of the shape of the response drive distribution and the encoding noise model on sensitivity (d'). **A** The impact of the shape of the response distribution on sensitivity for stimulus discriminability, assuming constant encoding noise. We computed expected sensitivity for a set of generalized Gaussian response drive distributions having identical means and variances but different powers. The generalized Gaussian is given by $p(R) \propto \exp\left[-\left|\frac{R}{\sqrt{2}\sigma}\right|^{p}\right]$. The Laplace distribution (p =1.0; kurtosis = 6.0) and the Gaussian (p =2.0; kurtosis = 3.0) are special cases. Powers larger than 2.0 approach the uniform distribution. **B** Expected sensitivity for broadband-normalized responses as a function of the response for constant (solid curve), Poisson-like (dashed curve), and quadratic (dotted curve) encoding noise. Quadratic encoding noise is predicted from a modulated-Poisson model of encoding noise{Goris:2018hp}. The histogram shows the distribution of stimulus-driven broadband response. The constant Gaussian encoding noise was set arbitrarily such that the expected sensitivity from the broadband responses across the stimulus ensemble was equal to 1.0 (i.e. $\sigma_I \cong \sigma_E/3$). The Poisson-like encoding noise is given by $\sigma_I = \sqrt{\alpha|R| + \sigma_0^2}$ where α is the fano factor and σ_0^2 are the fano factor and baseline variance, respectively. The values of the fano factor and the baseline variance were taken from the neurophysiological literature. The quadratic encoding noise is given by $\sigma_I = \sqrt{\beta|R|^2 + \sigma_0^2}$. To equate noise power across encoding noise types, the mean Poisson-like and quadratic encoding noise variance across the stimulus ensemble was matched to the constant encoding noise variance. **C** Expected sensitivity for narrowband-normalized responses as a function of the response. The mean encoding noise variance was matched to the mean encoding noise for the broadband responses. Sensitivity for stimulus discrimination is approximately 8% lower with Poisson-like encoding noise, and 6% lower with quadratic encoding noise. **D** Summary of expected sensitivity improvement for broadband and narrowband normalization, with constant, Poisson-like, and quadratic encoding noise.

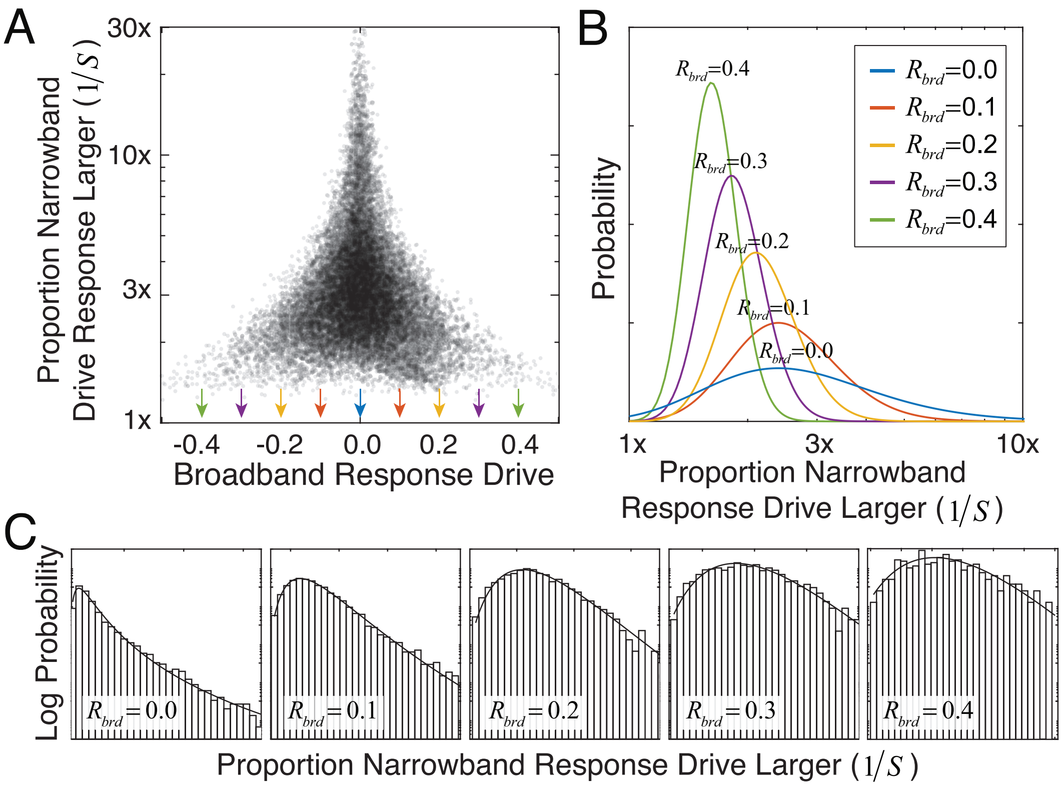

Figure S5. The impact of narrowband normalization. **A** Proportion that the narrowband response drive is larger than the broadband response drive R_{nrw}/R_{brd} as a function of the broadband response drive. Same data as Figure 5B in the main text. **B** Distribution of the proportional increase $p\left(R_{nrw}/R_{brd} \mid R_{brd}\right)$ in the narrowband response drive relative to the broadband response drive conditioned on different absolute values of the broadband response drive (colors), as fit by inverse gamma distributions. Arrows in A mark the absolute values of broadband response upon which the proportions are conditioned. Same data as Figure 5C in the main text. **C** Maximum likelihood fits of inverse gamma distributions to the distributions of proportional increase for five absolute values of the broadband response. Small broadband response drives tend to be amplified more than large broadband response drives. Note that the proportional increase on the x-axis in each of the five panels ranges, respectively, from 1.2x to 33x, 1.2x to 9x, 1.2x to 4.5x, 1.2x to 3.0x, and 1.2x to 2.4x, respectively.

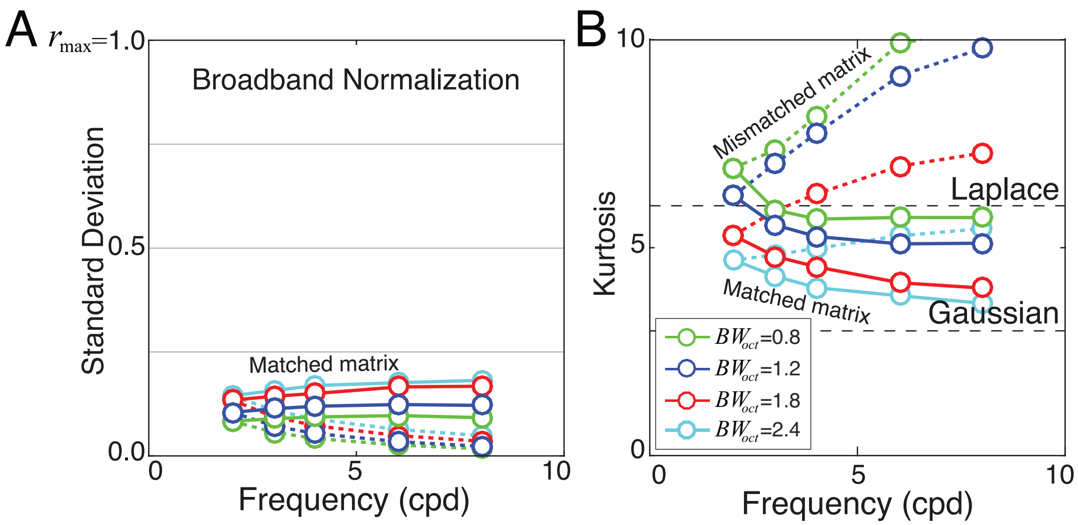

Figure S6. Response drive statistics with broadband normalization. **A** Stimulus-driven response drive standard deviation is invariant to preferred frequency when the receptive field's weight matrix is matched to the preferred feature (solid curves). Response drive standard deviation decreases systematically as the magnitude of the mismatch increases (dashed curves). **B** Stimulus-driven response kurtosis is consistent with a Gaussian when the weight matrix is matched to the preferred feature, but increases systematically as spatial frequency (and the magnitude of the mismatch) increases. Decreased stimulus-driven response drive variation (A) and increased response drive kurtosis (B) both decrease sensitivity for stimulus discrimination (Fig. S4).

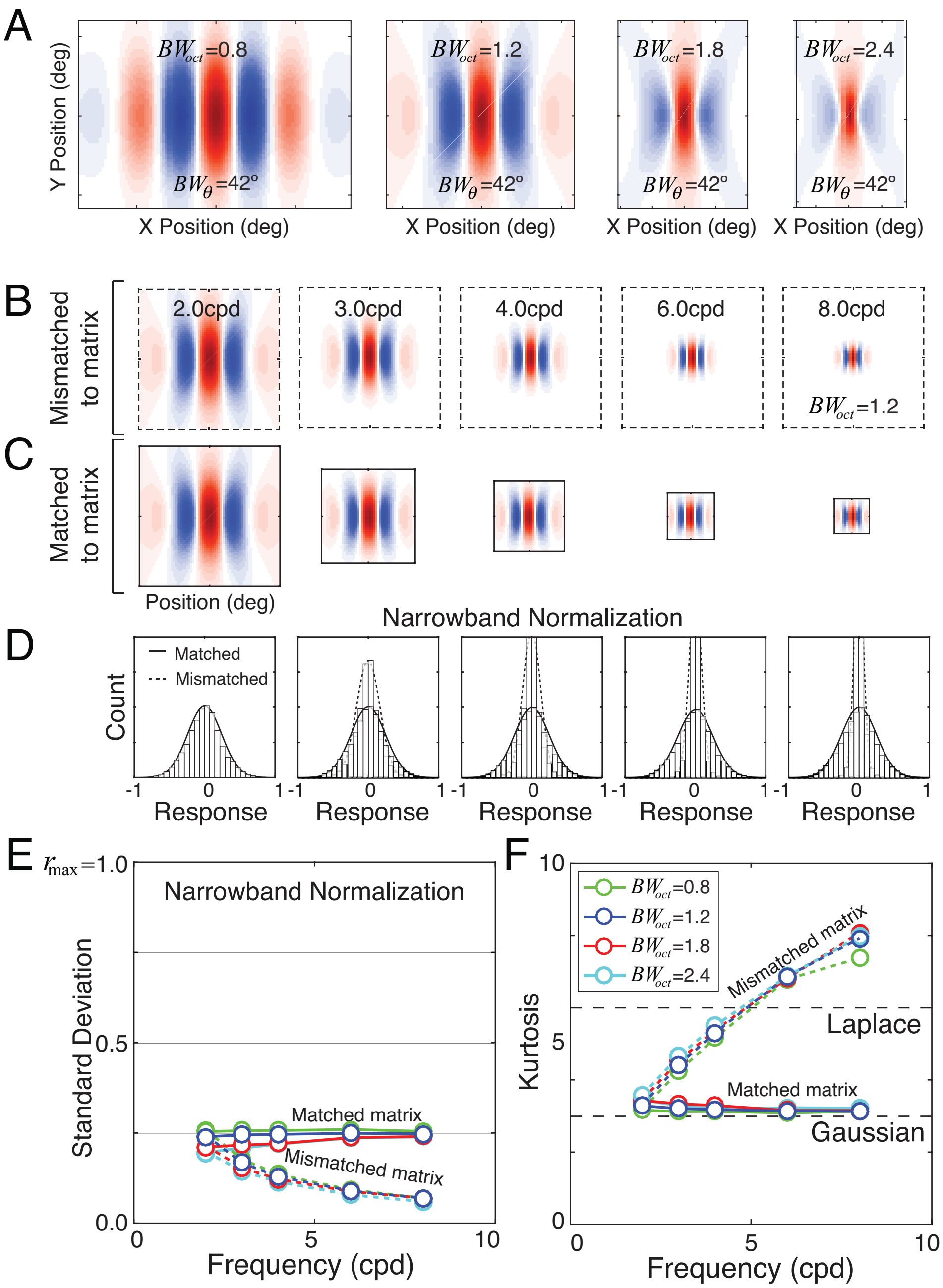

Figure S7. Log-Gabor receptive fields and response drive statistics. **A** Log-Gabor receptive fields with octave bandwidths of 0.8, 1.2, and 1.8, and orientation bandwidths of 42º. Different octave bandwidths correspond to preferred features with different aspect ratios (see Methods). The receptive fields **B** Mismatched and **C** matched receptive field weight matrices with 1.2 octave bandwidth for five preferred spatial frequencies. **D** Response drive distributions from matched and mismatched matrices. Matched weight matrices (solid curves) yield response drive distributions that are invariant to the scale of the preferred feature. Mismatched weight matrices (dashed curves) yield response drive distributions that change shape and variance with the magnitude of the mismatch. **E** Response drive standard deviation. Stimulus-driven response drive variance is constant with preferred frequency when the matrix is matched to the preferred feature. When mismatched, response drive standard deviation decreases with the magnitude of the mismatch. **F** Response drive kurtosis is the same as a Gaussian with matched weight matrices, but increases with the magnitude of the mismatch. Log-Gabor and Gabor response drive statistics are similar (see Fig. 7 in the main text).

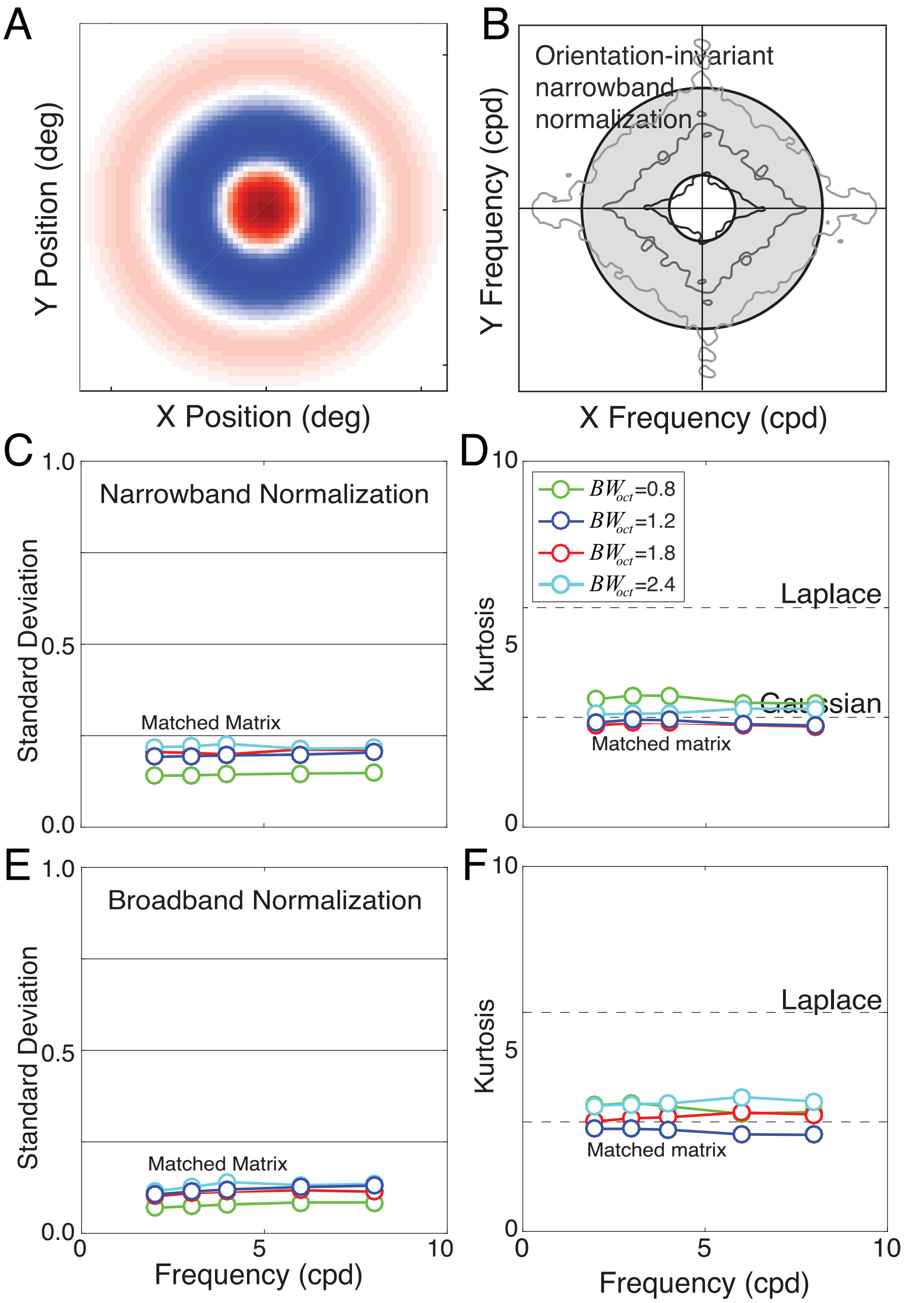

Figure S8. Response drive statistics for radial Gabor receptive fields with narrowband and broadband normalization. **A** Radially symmetric Gabor receptive field, similar to ganglion cell receptive fields in retina and relay cell receptive fields in lateral geniculate nucleus. **B** Schematic of pooling region in frequency space that determines the narrowband normalization factor. **C** Stimulus-driven response drive standard deviation with narrowband normalization for three different octave bandwidths (colors). **D** Stimulus-driven response drive kurtosis with narrowband normalization. **E** Response drive standard deviation with broadband normalization. **F** Response drive kurtosis with broadband normalization. Narrowband and broadband response statistics are more similar with radial Gabor receptive fields than with oriented Gabor receptive fields. Specifically, although broadband response drives still have lower stimulus-driven variance than narrowband response drives, both broadband and narrowband drives are approximately Gaussian.

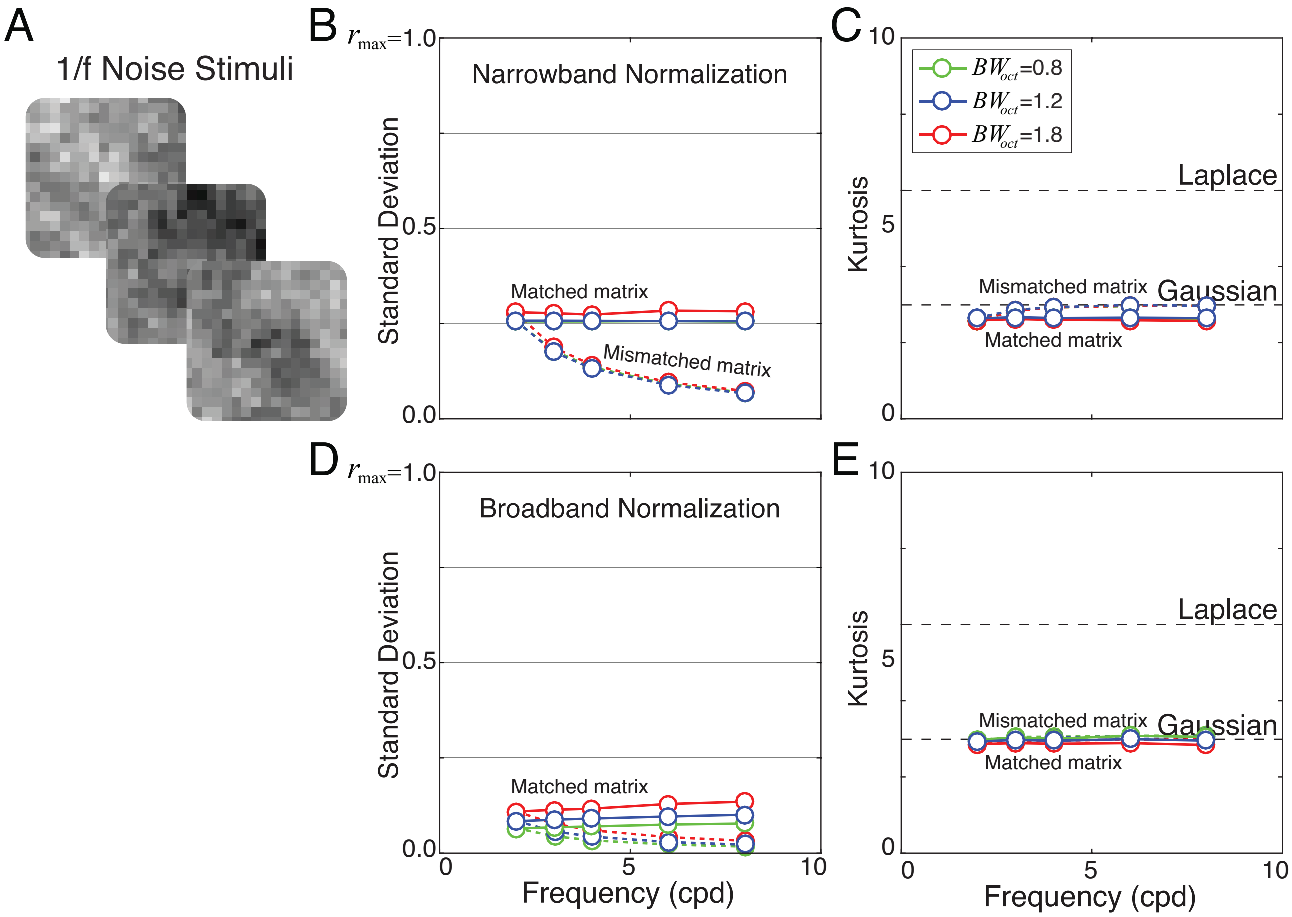

Figure S9. Response drive statistics caused by 1/f noise stimuli. Results are plotted for narrowband normalization with matched and mismatched weight matrices. **A** Example 1/f noise stimuli. 1/f noise stimuli were matched in contrast to the natural stimuli. **B** Stimulus-driven response drive standard deviation with narrowband normalization. **C** Stimulus-driven response drive kurtosis with narrowband normalization. Regardless of whether the matrices are matched or mismatched, the response drive kurtosis is consistent with a Gaussian. Note that it may appear surprising that with narrowband normalization, 1/f noise yields similar stimulus-driven response drive variance as the natural stimuli. (Broadband-normalized responses are substantially smaller with noise than with natural stimuli; see D). This is because our modeling assumes that the normalization factor is computed noiseless. This is not plausible for biological systems. Adding a small constant N_0 to the normalization term changes the response model to $R_{nrw} = \mathbf{f}^T\mathbf{c}/\left(N_{nrw} + N_0\right)$ and causes a substantial reduction in the stimulus-driven response drive variance to noise stimuli while leaving stimulus-driven response drive variance to natural stimuli relatively unaffected. The small constant has a larger effect on the response variance to noise stimuli because the (noiseless) narrowband normalization factor (i.e. similarity) tends to be much smaller for noise than for natural stimuli. Provided the constant is small enough, its addition to the response model does not appreciably change the distributional shape of the narrowband response drive distributions. **D** Response drive standard deviation with broadband normalization. The slight uptick in response drive standard deviation for the smaller matched matrices vanishes with downsampling. This increase is the only substantive difference we observed with downsampling. **E** Response drive kurtosis with broadband normalization.

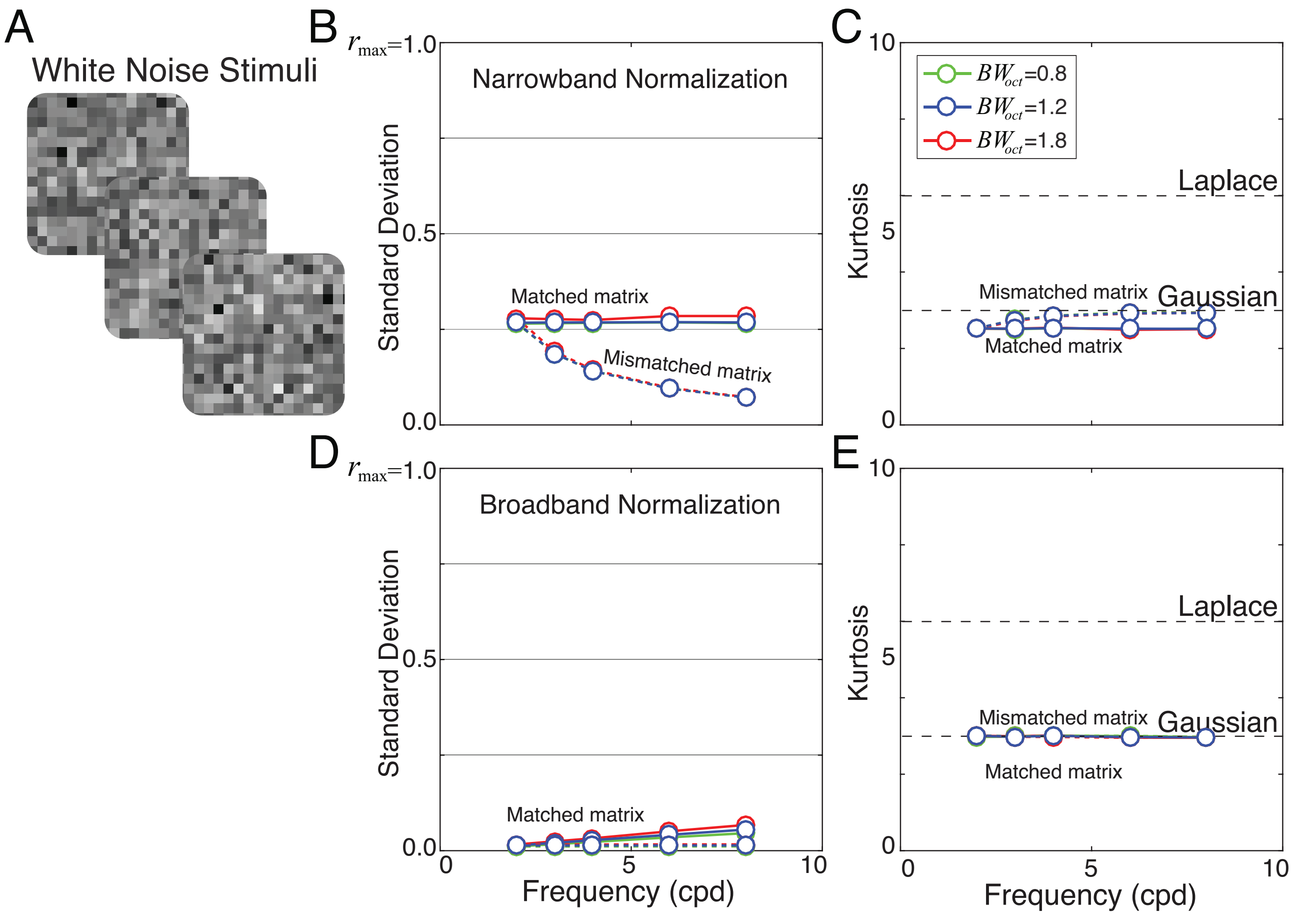

Figure S10. Response drive statistics to white noise stimuli. Results are plotted for narrowband normalization with matched and mismatched weight matrices. **A** Example 1/f noise stimuli. White noise stimuli were matched in contrast to the natural stimuli. **B** Stimulus-driven response standard deviation with narrowband normalization. **C** Stimulus-driven response kurtosis with narrowband normalization. Regardless of whether the matrices are matched or mismatched, the response kurtosis is consistent with a Gaussian. Note that it may appear surprising that with narrowband normalization, white noise yields similar stimulus-driven response variance as the natural stimuli. (Broadband-normalized responses are substantially smaller with noise than with natural stimuli; see D). This is because our modeling assumes that the normalization factor is computed without noise. This is not plausible for biological systems. Adding a small constant N_0 to the normalization term changes the response model to $R_{nrw} = \mathbf{f}^T\mathbf{c} / \left(N_{nrw} + N_0 \right)$ and causes a substantial reduction in the stimulus-driven response variance to noise stimuli while leaving stimulus-driven response variance to natural stimuli relatively unaffected. The small constant has a larger effect on the response variance to noise stimuli because the (noiseless) narrowband normalization factor (i.e. the similarities) tends to be much smaller for noise than for natural stimuli. Furthermore, because the phase-invariant similarity of white noise stimuli is lower than the phase-invariant similarity of 1/f noise stimuli to the receptive field, the constant drives down the stimulus-driven response variance to white noise stimuli more than to 1/f stimuli. Provided the constant is small enough, its addition to the response model does not appreciably change the distributional shape of the narrowband response distributions. **D** Response standard deviation with broadband normalization. The slight uptick in response standard deviation for the smaller matched matrices vanishes with downsampling. This increase is the only substantive difference we observed with downsampling. **E** Response kurtosis with broadband normalization.

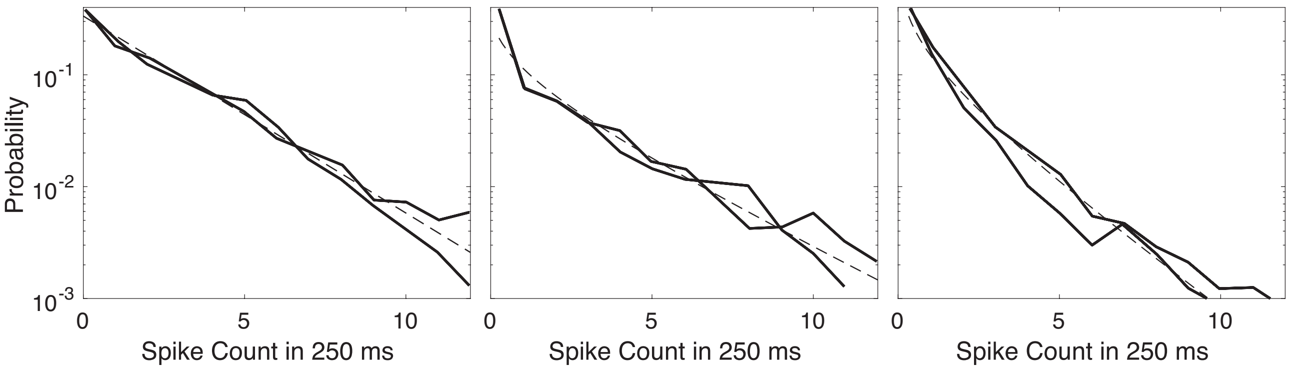

Figure S11. Spike response statistics elicited by natural stimuli from cat V1 neurons (adapted from Baddeley et al 1997). **A-C** Spike response distributions (thick lines; from Fig. 2a-c, Baddeley et al 1997), and scaled chi-squared distribution fits (dashed lines). Data in A are best fit with a scaled chi square distribution with 2 degrees of freedom (i.e. an exponential). Data in B, C are better fit by a chi-squared distribution with 1 degree of freedom (i.e. not an exponential). These chi-squared fits are the distributions predicted by Gaussian response drive statistics, given the standard models for complex and simple cells in area V1, respectively.

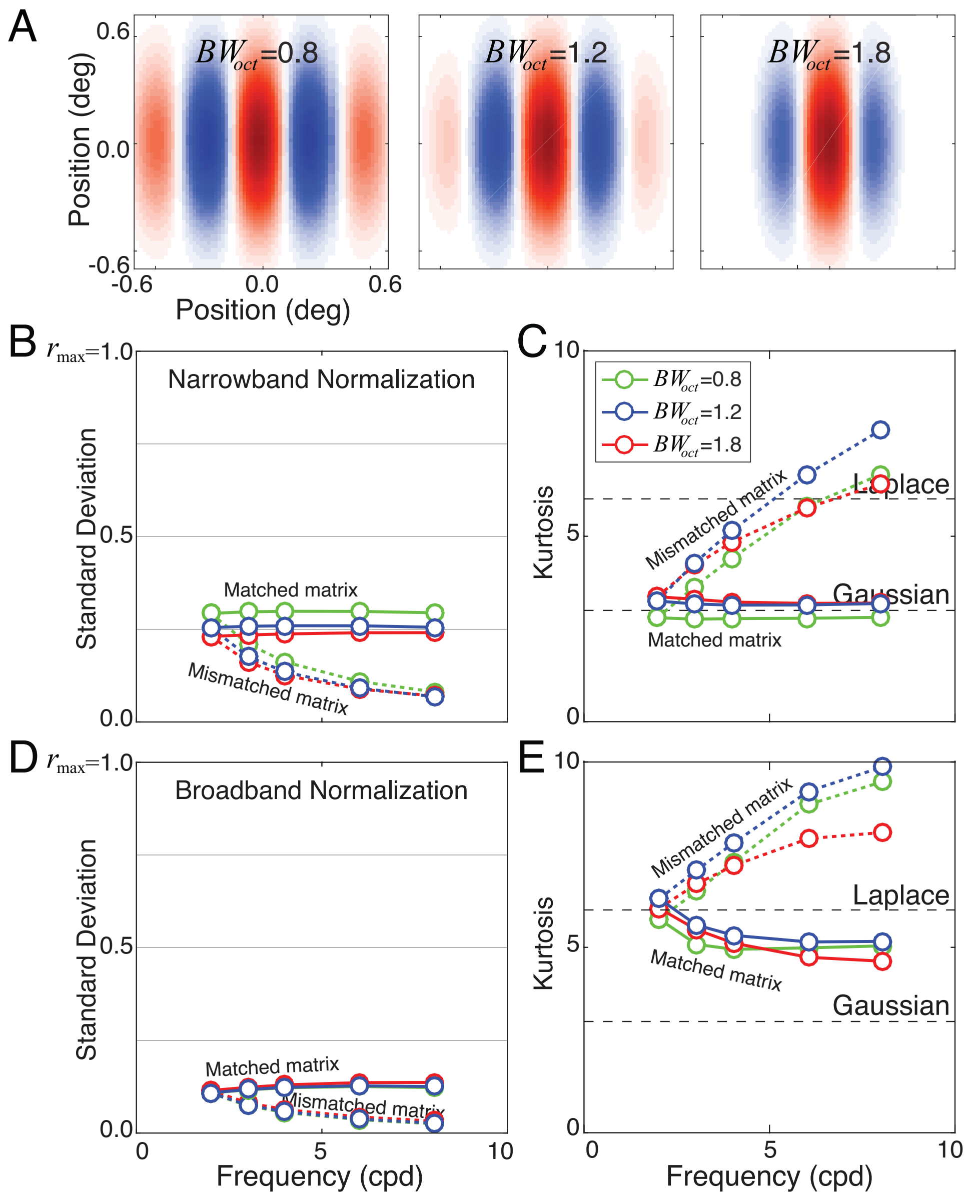

Figure S12. Response drive statistics with square weight matrices, which are often more convenient for modeling. Preferred features with different octave bandwidths have different aspect ratios. Thus, nominally matched square matrices are actually slightly mismatched for preferred features having octave bandwidths other than 1.2 (see Methods). **A** Preferred features in square weight matrices. **B** Stimulus-driven response drive standard deviation with narrowband normalization. Results for 'matched' square matrices are similar to the results with matched rectangular weight matrices presented in the main text. However, the 0.8 octave bandwidth preferred feature yields slightly higher stimulus-driven response variance and the 1.8 octave bandwidth feature yields slightly lower stimulus-driven variance than their rectangular counterparts. **C** Stimulus-driven response drive kurtosis with narrowband normalization. Results are very similar with square and rectangular weight matrices. **D** Response drive standard deviation with broadband normalization. **E** Response drive kurtosis with broadband normalization. Square weight matrices yield broadly similar results as rectangular weight matrices, and can probably be used for many applications interchangeably with rectangular matrices.

Supplement 1
Expected stimulus discriminability for Gaussian response distributions

Consider a neuron whose response can be modeled as a zero-mean Gaussian-distributed random variable r with standard deviation σ_E such that

$$r \sim N\left(0, \sigma_E^{\;2}\right) \qquad \text{(S1)}$$

Let r_1 and r_2 be two random response samples. The response difference $u = r_1 - r_2$ is also Gaussian distributed with a variance that is twice the variance of each of the i.i.d. responses

$$u \sim N\left(0, 2\sigma_E^{\;2}\right) \qquad \text{(S2)}$$

We are interested in the expected absolute difference $E\left[\left|u\right|\right] = E\left[\left|r_1 - r_2\right|\right]$ of two random responses. In general, the absolute value of a zero-mean Gaussian distributed random variable with variance σ^2 obeys a half-normal distribution with mean $\sqrt{\frac{2}{\pi}}\sigma$.

Given that $u = r_1 - r_2$ is a zero-mean Gaussian variable with variance $2\sigma_E^{\;2}$, we have

$$E\left[\left|r_1 - r_2\right|\right] = \sqrt{\frac{2}{\pi}}\sqrt{2}\sigma_E = \frac{2}{\sqrt{\pi}}\sigma_E \qquad \text{(S3)}$$

If the neuron's response is corrupted by encoding noise of variance $\sigma_I^{\;2}$, then the expected discriminability across stimuli for this neuron is given by

$$E\left[d'\right] = \frac{E\left[\left|r_1 - r_2\right|\right]}{\sigma_I} = \frac{2}{\sqrt{\pi}}\frac{\sigma_E}{\sigma_I} \qquad \text{(S4)}$$

Supplement 2
Expected stimulus discriminability for Laplace response distributions

Consider a neuron whose response can be modeled as a zero-mean Laplace-distributed random variable r with standard deviation σ_E such that

$$r \sim f(r) = \frac{1}{\sqrt{2}\sigma_E} e^{-\frac{|r|}{\sigma_E/\sqrt{2}}}, -\infty < r < \infty \qquad \text{(S5)}$$

Let r_1 and r_2 be two random response samples. The response difference $u = r_1 - r_2$ is the difference of two i.i.d. responses. Let $u \sim g(u)$

$$g(u) = \int_{-\infty}^{\infty} f(x) f(x-u)\, dx$$

$$g(u) = \int_{-\infty}^{\infty} \left(\frac{1}{\sqrt{2}\sigma_E} e^{-\frac{|x|}{\sigma_E/\sqrt{2}}} \right) \left(\frac{1}{\sqrt{2}\sigma_E} e^{-\frac{|x-u|}{\sigma_E/\sqrt{2}}} \right) dx$$

$$g(u) = \frac{1}{2\sigma_E^2} \int_{-\infty}^{\infty} e^{-\frac{|x|+|x-u|}{\sigma_E/\sqrt{2}}} dx \qquad \text{(S6)}$$

The integral cannot be simply evaluated with an absolute value in the integrand. To remove the absolute value from the integrand, we split the integral depending on the values that u and x take. We note that $g(u)$ is even symmetric. Thus, solving the integral for all values of $u > 0$ will provide the solution to the integral for all values of $u < 0$. Assuming that $u > 0$, then $|x| + |x-u| = u - 2x$ when $-\infty < x < 0$, $|x| + |x-u| = u$ when $0 < x < u$, and $|x| + |x-u| = 2x - u$ when $u < x < \infty$. Evaluating for cases when $u > 0$ yields

$$g(u) = \frac{1}{2\sigma_E^2} \left(\int_{-\infty}^{0} e^{\frac{2x-u}{\sigma_E/\sqrt{2}}} dx + \int_{0}^{u} e^{-\frac{u}{\sigma_E/\sqrt{2}}} dx + \int_{u}^{\infty} e^{\frac{-2x+u}{\sigma_E/\sqrt{2}}} dx \right), \qquad u > 0$$

$$= \frac{1}{2\sigma_E^2} \left(\left[\frac{\sigma_E}{2\sqrt{2}} e^{\frac{2x-u}{\sigma_E/\sqrt{2}}} \right]_{-\infty}^{0} + \left[x e^{\frac{-u}{\sigma_E/\sqrt{2}}} \right]_{0}^{u} + \left[\frac{-\sigma_E}{2\sqrt{2}} e^{\frac{-2x+u}{\sigma_E/\sqrt{2}}} \right]_{u}^{\infty} \right), \quad u > 0$$

$$= \frac{1}{2\sigma_E^2} \left(\frac{\sigma_E}{2\sqrt{2}} e^{\frac{-u}{\sigma_E/\sqrt{2}}} + u e^{\frac{-u}{\sigma_E/\sqrt{2}}} + \frac{\sigma_E}{2\sqrt{2}} e^{\frac{-u}{\sigma_E/\sqrt{2}}} \right), \qquad u > 0$$

$$= \frac{1}{2\sigma_E^2} \left(\frac{\sigma_E}{\sqrt{2}} e^{\frac{-u}{\sigma_E/\sqrt{2}}} + u e^{\frac{-u}{\sigma_E/\sqrt{2}}} \right), \qquad u > 0 \qquad \text{(S7)}$$

Given that $g(u)$ is even-symmetric, we can replace u with $|u|$ in Eq. S7

$$g(u) = \frac{1}{2\sigma_E^2} \left(\frac{\sigma_E}{\sqrt{2}} e^{\frac{-|u|}{\sigma_E/\sqrt{2}}} + |u| e^{\frac{-|u|}{\sigma_E/\sqrt{2}}} \right) \qquad \text{(S8)}$$

After distributing the leading scale factor $1/\sigma_E^2$ and rearranging terms, we obtain an function where the two terms in the sum are the expressions for a Laplace distribution and a bilateral Gamma distribution

$$g(u)=\frac{1}{2}\left(\overbrace{\frac{1}{\sqrt{2}\sigma_E}e^{\frac{-|u|}{\sigma_E/\sqrt{2}}}}^{\text{Laplace distribution}}+\overbrace{\frac{1}{\sqrt{2}\sigma_E}\frac{|u|}{\sigma_E/\sqrt{2}}e^{\frac{-|u|}{\sigma_E/\sqrt{2}}}}^{\text{Bilateral Gamma Distribution}}\right) \quad \text{(S9)}$$

Recall that we are interested in the expectation of the absolute value of $|u|=|r_1-r_2|$, not the expectation of $u=r_1-r_2$ itself. Computing the expectation $E\left[|u|\right]$ using $g(u)$ from the definition of expectation

$$E\left[|u|\right]=\int_{-\infty}^{\infty}|u|g(u)\,du$$

$$E\left[|u|\right]=\frac{1}{2}\int_{-\infty}^{\infty}|u|\left(\frac{1}{\sqrt{2}\sigma_E}e^{\frac{-|u|}{\sigma_E/\sqrt{2}}}+\frac{1}{\sqrt{2}\sigma_E}\frac{|u|}{\sigma_E/\sqrt{2}}e^{\frac{-|u|}{\sigma_E/\sqrt{2}}}\right)du$$

Noting that the integrand is an even function means that twice the integral from zero to infinity equals the integral from negative infinity to infinity

$$E\left[|u|\right]=\int_{0}^{\infty}\left(\frac{|u|}{\sqrt{2}\sigma_E}e^{\frac{-|u|}{\sigma_E/\sqrt{2}}}+\frac{1}{\sigma_E^2}|u|^2e^{\frac{-|u|}{\sigma_E/\sqrt{2}}}\right)du$$

On the positive real line, we can drop the absolute value symbols

$$E\left[|u|\right]=\int_{0}^{\infty}\left(\frac{u}{\sqrt{2}\sigma_E}e^{\frac{-u}{\sigma_E/\sqrt{2}}}+\frac{1}{\sigma_E^2}u^2e^{\frac{-u}{\sigma_E/\sqrt{2}}}\right)du$$

Splitting the integral

$$E\left[|u|\right]=\frac{1}{\sqrt{2}\sigma_E}\int_{0}^{\infty}ue^{\frac{-u}{\sigma_E/\sqrt{2}}}\,du+\frac{1}{\sigma_E^2}\int_{0}^{\infty}u^2e^{\frac{-u}{\sigma_E/\sqrt{2}}}\,du \quad \text{(S10)}$$

Each of the two definite integrals in Eq. S6 can be computed with the standard result

$$\int_{0}^{\infty}u^ne^{-au}\,du=\frac{n!}{a^{n+1}},n\in W,a>0$$

Plugging in

$$E\left[|u|\right]=\frac{1}{\sqrt{2}\sigma_E}\left[\frac{1}{\left(\frac{1}{\sigma_E/\sqrt{2}}\right)^2}\right]+\frac{1}{\sigma_E^2}\left[\frac{2}{\left(\frac{1}{\sigma_E/\sqrt{2}}\right)^3}\right]$$

Simplifying terms

$$\begin{aligned}E\left[|u|\right]&=\frac{1}{\sqrt{2}\sigma_E}\left[\frac{\sigma_E^2}{2}\right]+\frac{1}{\sigma_E^2}\left[\frac{2\sigma_E^3}{2\sqrt{2}}\right]\\&=\frac{\sigma_E}{2\sqrt{2}}+\frac{\sigma_E}{\sqrt{2}}\\&=\frac{3}{2\sqrt{2}}\sigma_E\end{aligned}$$

Therefore, the mean absolute difference between two i.i.d. mean-zero Laplace random variables of standard deviation σ_E is

$$E\left[|u|\right]=E\left[|r_1-r_2|\right]=\frac{3}{2\sqrt{2}}\sigma_E \tag{S11}$$

For internal noise of standard deviation σ_I, the expected stimulus discriminability across all stimuli is given by

$$E\left[d'\right]=\frac{E\left[|r_1-r_2|\right]}{\sigma_I}=\frac{3}{2\sqrt{2}}\frac{\sigma_E}{\sigma_I} \tag{S12}$$
